# An Alkaloid Biosynthetic Gene Bundle in Animals

**DOI:** 10.1021/jacs.5c18350

**Published:** 2026-01-08

**Authors:** Jun Gu Kim, Zhenjian Lin, Vinayak Agarwal, Eric W. Schmidt

**Affiliations:** † Department of Medicinal Chemistry, 7060 University of Utah, Salt Lake City, Utah 84112, United States; ‡ School of Chemistry and Biochemistry, Georgia Institute of Technology, Atlanta, Georgia 30332, United States; § School of Biological Sciences, Georgia Institute of Technology, Atlanta, Georgia 30332, United States

## Abstract

Alkaloids include some of the most impactful molecules used in science and medicine. While plant alkaloids are well explored, much less is known about the diverse bioactive alkaloids from the animal kingdom. To solve this problem, we developed a computational method to discover genes that are bundled in chromosomal regions, enabling agnostic discovery of noncanonical biosynthetic gene clusters (BGCs) without prior knowledge of what enzymes might be involved. The method was applied to marine sponges that produce oroidin and related pyrrole-imidazole alkaloids, uncovering 36 BGCs in oroidin-producing sponges, only one of which (*oro*) was found in all species. Many of these clusters defy current BGC dogma, leading us to suggest the name “bundles” for this phenomenon. Five *oro* proteins were validated in biochemical assays. *oro* consists of orthologs of common, animal-specific primary metabolic genes that have been collected in one chromosomal region and repurposed for alkaloid biosynthesis. This provides a roadmap to accelerate the development of oroidins and the countless other unique natural products found in the animal kingdom.

## Graphical abstract

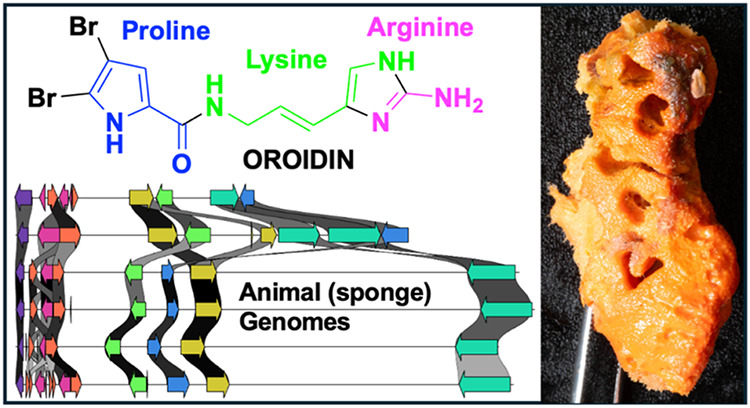

## Introduction

Animals produce thousands of different alkaloids.^1^ Unlike plants, which are mainstays of alkaloid-based biotechnology and medicine,^2^ the genes and proteins underlying animal alkaloid biosynthetic pathways are essentially unknown, making it difficult to develop promising compounds. For example, the animal phylum Porifera (sponges) is the source of numerous alkaloid structural classes, including many drug leads.^3^ Among these, the pyrrole-imidazole alkaloids (PIAs) represent a large class of more than 150 marine natural products that have been extensively studied because of their therapeutic potential, their complex and interesting chemistry, their abundant presence in several widespread sponge species, and their unique and poorly understood biosynthesis (Figure 1).^4^

**Figure 1:**
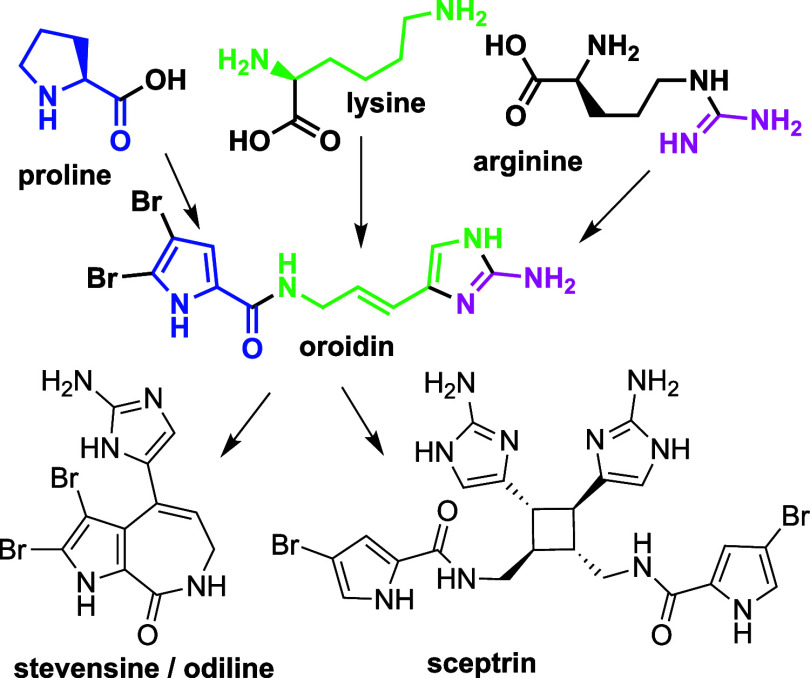
Pyrrole-imidazole alkaloids (PIAs) from marine sponges. Oroidin is synthesized from proline, lysine, and arginine. Downstream, single electron transfer and other reactions are thought to convert oroidin and its structural relatives into more complex PIAs, leading to a family of >150 bioactive animal-derived alkaloids.

The simplest PIAs are oroidin and its close structural relatives. Using metabolomics, Mohanty et al.^4^ proposed a pathway to oroidin involving the coupling of proline-derived dibromopyrrole with lysine-derived homoagmatine derivatives. Once oroidin is formed, it is converted to more complex metabolites,^5^ some of which are made via a single electron transfer pathway.^6^ Production of analogs in sponge cell culture^7^ and a lack of correlated bacteria in the microbiome^4^ suggested that, unlike most sponge pathways characterized to date, the PIAs might be made by the sponges themselves, and not by the symbiotic bacteria that make so many bioactive sponge compounds. However, the true producing organism—and what genes and enzymes might be involved in the production of PIAs—remained unknown.

Biosynthetic pathways are often found by analogy to expected genes. A major limitation we faced here is that very little is known about how animals make alkaloids, leaving us with a lack of gene targets on which to base a search. Even when we did suspect a specific gene type, hundreds of related genes were present in the animal genomes, with no obvious method of prioritization. In microbes, genes often co-occur as biosynthetic gene clusters (BGCs).^8^ These greatly simplify pathway discovery because finding one gene identifies the rest of the pathway. While some types of animal biosynthetic pathways are at least partly clustered,^9^ most of them appear not to be, at least not in the conventional sense. Thus, none of the modern tools used for microbial pathway discovery would apply. Moreover, little is known about animal biosynthesis in general, making it still very difficult to apply the revolutionary methods now commonly used in the plant biosynthesis field.

Here, we developed a method to perform untargeted searches for all potential biosynthetic genes that maintain spatial relationships in the genome and applied that method to discover how sponge animals make alkaloids. Our method uncovered an unexpected richness of conserved potential biosynthetic pathways without relying on a prior biosynthetic hypothesis. Among these, we found a bundle of genes, *oro*, that was conserved in PIA producers but was absent from closely related species that did not produce PIAs. *oro* is not a BGC in the classical sense, in that it spans a large sponge chromosomal region and is variably interspersed with nonbiosynthetic genes in different sponge species. The *oro* pathway was validated by functional expression and biochemical analysis of several proteins. Among these, decarboxylase (OroE) is highly selective for homoarginine over arginine and related amino acids, yielding a tool that is potentially useful in synthetic and chemical biology. Furthermore, OroF is the first example of an animal-derived flavin adenine dinucleotide (FAD)-dependent halogenase; its relationship to proteins normally involved in endoplasmic reticulum protein degradation^10^ revealing a unique fold and enzymatic motif supporting halogenation in the animals and opening new directions for biocatalyst discovery and natural product biosynthesis research.

## Results

### Targeting the Unknowns in Animal Genomes

Analysis of previously characterized animal biosynthetic pathways revealed that core^9^ biosynthetic genes are often syntenic in animals that produce similar compounds. However, unlike classical BGCs in which the biosynthetic genes are physically adjacent on the chromosome, animal biosynthetic genes are frequently interspersed with nonsyntenic genes that are unrelated to the biosynthesis. These interspersed unrelated genes, and the distances between biosynthetic genes on chromosomes, are highly variable, making it challenging to use traditional genome mining methods. Another issue is the paucity of known biosynthetic genes in animals. For example, in our initial attempts to uncover PIA pathways in the sponge *Axinella corrugata*, we found 188 ligases, 106 cytochromes P450 (CYPs), 104 monooxygenases, and 90 decarboxylases, which were not obviously clustered and could not be readily prioritized using available methods.

To address this problem, we designed the SynBGC bioinformatics pipeline (Figures S1 and S2). SynBGC employs a database of all biosynthetic gene motifs found in minimum information about a biosynthetic gene cluster (MIBiG),^11^ uses them to collect bundles of potential biosynthetic genes from animal genomes, and then compares those bundles across a set of animal genomes. SynBGC thus finds spatially related bundles of biosynthetic genes regardless of variability of intervening nonbiosynthetic genes and variation in biosynthetic gene order and content, and without the need for a prior hypothesis about which genes might be involved in a pathway. SynBGC has several advantages in comparison to available methods. It does not preselect potential candidate biosynthetic genes, but instead agnostically uses a broad set of gene families from the MIBiG database,^11^ treating any of them as potential cores. SynBGC groups genes into clusters based on being bundled into the same gene neighborhood, regardless of position, orientation, or colinearity. Subsequently, SynBGC reaches beyond initially discovered clusters, bundling broadly spaced genes together to produce large candidate bundles of clusters. Finally, SynBGC compares candidate clusters between compound producing and nonproducing organisms, ensuring that only functionally relevant BGCs are retained. It thus provides an efficient method to find spatial relationships between genes linked to natural products, whether or not those genes are canonically involved in making natural products.

### Abundant Biosynthetic Gene Bundles in Sponge Genomes

To validate the SynBGC pipeline, we first selected seven mollusc and four soft coral genome assemblies from the NCBI database (Table S1), aiming to determine whether we could rediscover previously validated bursatellin-oxazinin and terpene BGCs.^9,12^ The method rapidly rediscovered the known clusters (Figures S3–S5). Next, we applied SynBGC to oroidin biosynthesis. Genomes and transcriptomes were obtained or assembled from GenBank for five PIA-containing sponge species (*Agelas conifera*, *Agelas oroides*, *Agelas tubulata*, *Axinella corrugata*, and *Axinella damicornis*) and three related sponges that are not known to produce PIAs (*Axinella polypoides*, *Cymbastella concentrica*, and *Phakellia robusta*) (Table S2). In total, 355,972 protein-coding sequences were retrieved, of which 9,655 (average 2.7%) survived the first filtering step for biosynthetic genes. Application of SynBGC produced a gene cluster network (threshold of 0.5 raw distance) with 59 distinct clusters found in multiple sponge species and many singletons found only in a single species (Figure 2A).

**Figure 2:**
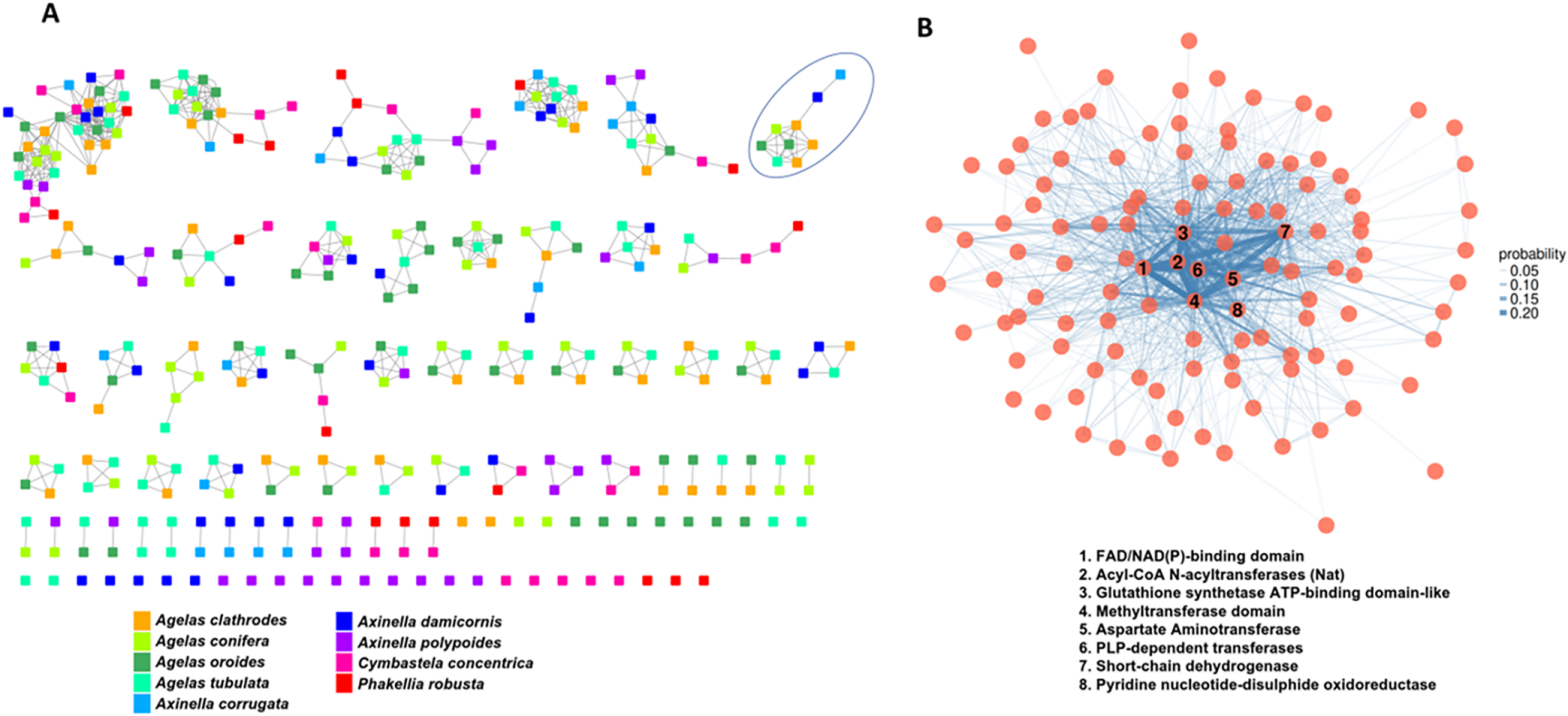
SynBGC uncovers biosynthetic gene bundles (BGCs). (A) BGCs found in PIA-producing sponges and their close relatives. The circle indicates the *oro* cluster. Colors indicate source sponge species. Network created with BiG-SCAPE.^14^ (B) Protein function co-occurrence in sponge BGCs created with ggraph (https://CRAN.R-project.org/package=ggraph). Orange nodes represent individual domains. Blue edges connect domain pairs that are found together in at least one BGC, with edge thickness proportional to the probability of co-occurrence (i.e., the fraction of BGCs in which a given domain pair is observed. probability = cooccurrence/total_clusters). Higher probabilities indicate that the two domains are found together in a greater number of BGCs. Only edges with probability >0.025 are shown. Labeled domains 1–8 are among the top 20 most abundant co-occurrences.

In comparison to the known BGCs in the MIBiG database, the identified BGCs showed no clear relationship to characterized microbial or plant biosynthetic pathways, and when viewed in their full genomic context, most were heavily interspersed with nonbiosynthetic genes that had been excluded during the filtering step. To gain a broad overview of the identified gene bundles, we plotted the co-occurrence probabilities between protein domains within the same bundle (Figure 2B). The most frequently co-occurring domains—such as FAD/nicotinamide adenine dinucleotide (phosphate) (NAD­(P))-binding, acyl-coenzyme A (CoA), *N*-acyltransferase, methyltransferase, iron 2-oxoglutarate dioxygenase, and cytochrome P450—are widely distributed in eukaryotic central metabolism and are not specific to the core biosynthetic enzymes of BGCs identified by current mining tools.

Finally, we applied SynBGC more widely to plant and vertebrate species. We successfully identified thalianol and baruol BGCs in two *Arabidopsis* species (*thaliana* and *lyrata*), but not in *Arabidopsis arenosa*, which does not produce the compounds (Table S1 and Figure S6). SynBGC was applied to vertebrate genomes (Table S1), where no specific natural product target is available. We detected multiple biosynthetic like conserved clusters across vertebrate classes. A striking example is that in 17 of 23 vertebrate genomes examined (Table S1 and Figure S7), creatine biosynthetic genes glycine amidinotransferase (GAMT), AMP-dependent synthetase/ligase gene, and histidine decarboxylase genes are clustered (example: KYO21137.1, XP_006259418.1, and KYO21140.1 from *Alligator mississippiensis*). Since each genome contains only a single GAMT gene, it is unlikely that GAMT participates in biosynthesis beyond creatine. We also observed a cluster including lanosterol synthase and sterol 3-β-glucosyltransferase genes, along with other genes, in at least four vertebrate genomes from birds (*Meleagris gallopavo*), amphibians (*Xenopus tropicalis*), coelacanths (*Latimeria chalumnae*), and lungfish (*Protopterus annectens*), but which is absent from most vertebrate genomes. The distance between lanosterol synthase and the glucosyltransferase varies, ranging from 4 to 19 intervening genes; speculatively, products may resemble echinoderm compounds.^13^ It is notable that these “clusters” do not meet the standard definition of a BGC, and therefore to avoid confusion we suggest the terminology “biosynthetic gene bundle” may be considered.

### Proposed *oro* Biosynthetic Pathway to Oroidin

Notably, 36 of 59 BGCs were found exclusively in PIA-producing species, and only one (dubbed “*oro*”) was present in all six PIA-producing species, making it a strong candidate for oroidin production. In all six species, the *oro* BGC (GenBank PX316405) encoded a set of enzymes consistent with the biosynthetic logic proposed for oroidin biosynthesis by Mohanty et al.,^4^ but with different gene arrangements and interspersed nonbiosynthetic genes in each sponge species (Figure 3). CoA ligase OroD and acyltransferase OroG would activate an acid such as proline or 4,5-dibromo-1H-pyrrole-2-carboxylic acid and couple it with an amine donor, homoagmatine or derivative thereof. In turn, homoagmatine would be synthesized by the actions of amidinotransferase OroA and decarboxylase OroE. A series of oxidases, including two cytochromes P450 (CYPs) OroB and OroC, would be involved in converting proline and/or homoagmatine into advanced derivatives en route to oroidin, while flavin-dependent monooxygenase (FMO) OroF was likely to act as a pyrrole halogenase. However, the order of transformations leading from these primary metabolites to oroidin was unclear, with many possible routes. For example, four possible proline derivatives and at least seven possible lysine derivatives might be substrates for acyltransferase coupling.

**Figure 3:**
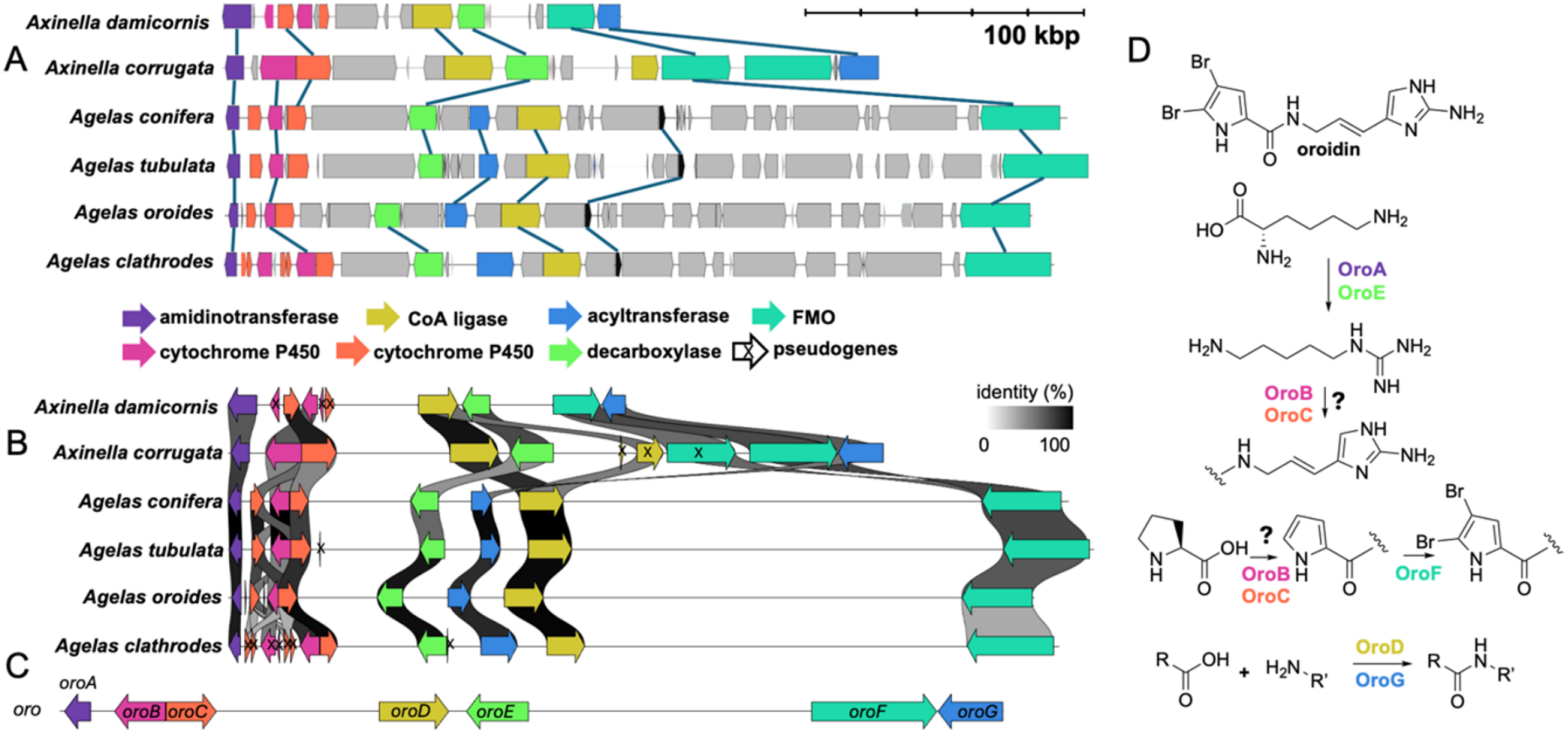
The *oro* BGC and proposed oroidin biosynthetic pathway. (A) Synteny plot of *oro* BGCs from sponges, made using clinker.^15^ Note the semiconserved gene order and the intervening, seemingly random nonbiosynthetic genes. (B) Same pathways as in panel A, but with noncoding genes removed for clarity, and showing percent identity between *oro* genes, prepared using clinker. (C) Focus on biosynthetic genes from the *A. corrugata* *oro* cluster. (D) Proposed biosynthesis of oroidin. The roles of OroA, D, E, F, and G are somewhat predictable from sequence, but reactions catalyzed by oxidases OroB, OroC, and their timing in the pathway, are not obvious. For example, the coupling partners for OroD and OroG could be any of the compounds shown below the oroidin molecule.

Since there is no genetic model for these sponges, we expressed in *Escherichia coli* (Figure S8) and characterized *oro*-derived proteins from *A. corrugata*, a PIA producer. By demonstrating the functions of the enzymes in vitro and exploring their substrate selectivities, we provide evidence supporting the assignment of *oro* as an animal alkaloid biosynthetic pathway to oroidin and define the probable biosynthetic order of key steps in the pathway.

### OroA and OroE Selectively Convert Lysine to homoagmatine

OroA was predicted to encode an amidinotransferase, a widespread biosynthetic enzyme class that donates the arginine amidino group to acceptor molecules. For example, some amidinotransferases add the amidino group to glycine or to polyamines.^16,17^ Others transfer the amidino group to amino acids, either at the α amine as found in saxitoxin biosynthesis^18^ or the side-chain amine.^19^ Notably, several amidinotransferases prefer lysine as a substrate and produce homoarginine.^20,21^ Thus, we anticipated that the same role might be played by OroA. Following synthesis of homoarginine, the biosynthetic logic requires decarboxylation to homoagmatine. Analysis of the OroE sequence suggested that the enzyme belonged to a family of eukaryotic pyridoxal-5′-phosphate-(PLP)-dependent group IV decarboxylases.^22–26^ Most of decarboxylase in this family use ornithine, lysine, or arginine as substrates. An enzyme from the plant *Lathyrus sativus* decarboxylates homoarginine, but with a 4-fold preference for lysine.^27^

Initial expression of intact OroA and OroE in *E. coli* BL21­(DE3) resulted in extensive inclusion body formation. Sequence analysis using BlastP, SignalP,^28^ and AlphaFold3 ^29^ indicated that the N-terminal region does not contain a signal peptide but instead lacks predicted structure in the AlphaFold model, which we have found is sometimes correlated with poor folding. Recombinant OroA, obtained after N-terminal truncation (Figure S9), was combined with arginine as the amidino donor and with varying substrates, including lysine, 1,5-diaminopentane, and glycine. Robust conversion of lysine to homoarginine was observed, while 1,5-diaminopentane was converted much more slowly, and glycine was not a substrate (Figures 4A and S10). The formation of homoarginine was validated by mass spectrometry of the reaction products, as well as chemical derivatization in comparison with a standard by MS^2^ (Figures S11 and S12). The kinetic parameters of OroA, estimated under initial velocity conditions (Figure S13), were *K*_m_ = 55.6 μM, *k*_cat_ = 1.06 min^–1^ for lysine and *K*_m_ = 1,960 μM, *k*_cat_ = 0.156 min^–1^ for 1,5-diaminopentane (Figure S14). These results suggested the preference for lysine as substrate, supporting that OroA is a selective homoarginine synthase.

**Figure 4:**
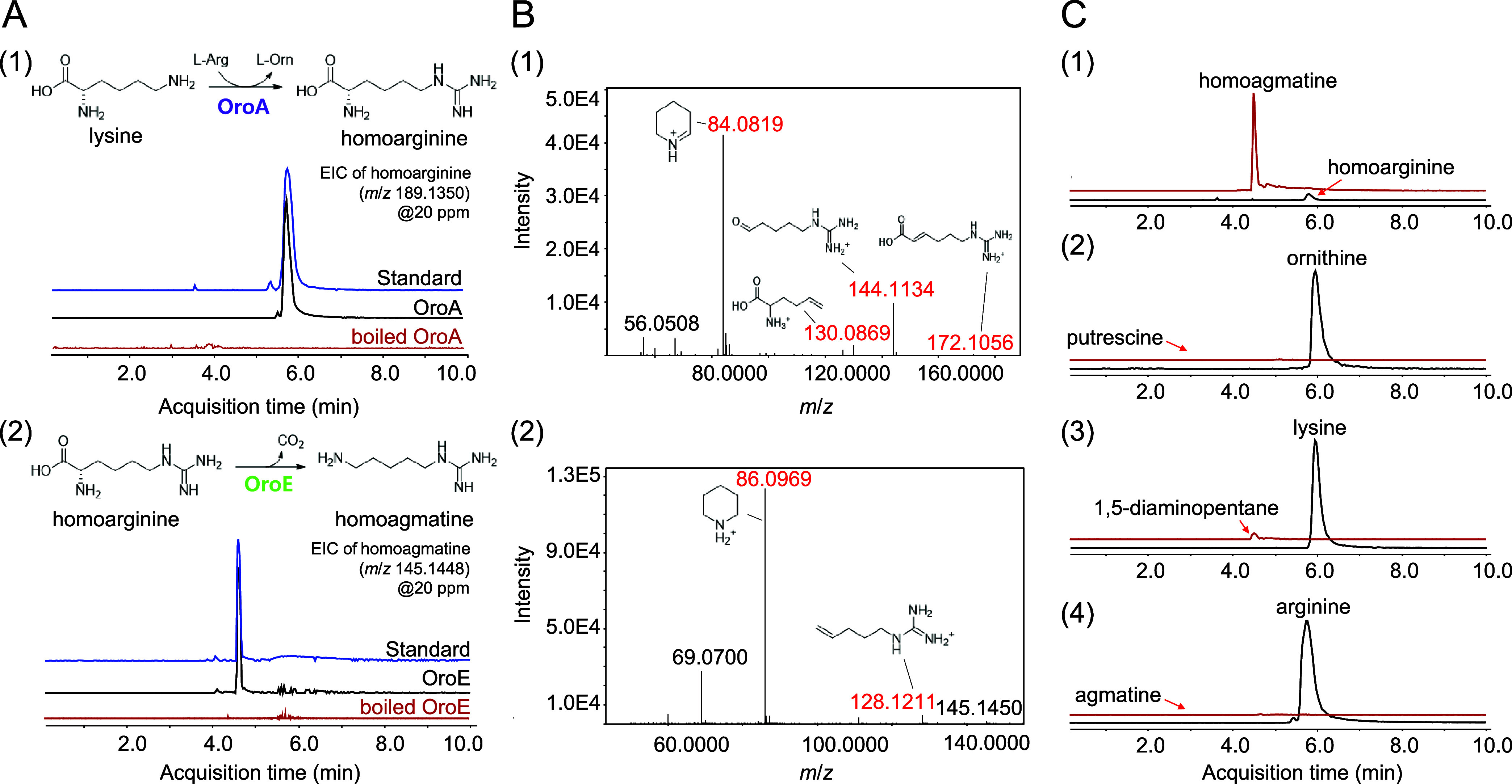
LC-MS analysis of OroA and OroE enzyme reactions. (A) Extracted ion chromatograms (EICs) of (1) OroA and (2) OroE in vitro assays. (B) MS/MS spectra of enzyme reaction products; (1) homoarginine, (2) homoagmatine. (C) Substrate specificity of OroE. The black traces represent the chromatogram of remaining substrate, while the red traces correspond to the product.

Incubation of recombinant purified OroE (N-terminal truncation, Figure S9) with homoarginine and PLP for 2 h resulted in the formation of homoagmatine. The identity of the product was confirmed by comparison of its retention time and MS^2^ spectrum with those of a synthetic standard (Figures 4A,4B, and S12). Intriguingly, the enzyme showed no activity toward ornithine or arginine, while lysine was converted to 1,5-diaminopentane only to a minimal extent after 20 h (Figure 4C). OroE kinetic parameters with homoarginine were estimated as *K*_m_ = 187.69 μM, *k*_cat_ = 2.10 min^–1^, and *k*_cat_/*K*_m_ = 1.12 × 10^–2^ μM^–1^min^–1^ (Figure S14). Related eukaryotic decarboxylases exhibit *K*_m_ values between 91 and 3,330 μM, using ornithine, lysine, or arginine as substrates.^22–25,27^ By comparison, the *L*. *sativus* enzyme prefers lysine (*K*_m_ = 880 μM) over homoarginine (*K*_m_ = 3,330 μM).^27^

As the most selective and specific homoagmatine biosynthesis proteins yet described, OroA and OroE provide strong support for the *oro* BGC and exactly match the biosynthetic proposal of Mohanty et al.^4^

### Amide Bond Formation by OroD and OroG

The presence of CoA synthetase OroD and acyltransferase OroG suggested a pathway in which proline, pyrrole-2-carboxylic acid, or 4,5-dibromo-1H-pyrrole-2-carboxylic acid is activated as a CoA thioester and then aminated by homoagmatine or a derivative thereof. Purified OroD was incubated with all three potential carboxylate substrates in the presence of ATP, CoASH, and MgCl_2_ for 2 h. However, no detectable CoA products were observed (Figure S15). Both prolyl-CoA and prolyl-*N*-acetyl cysteamine (SNAC) thioesters were previously reported to be highly unstable, and essentially invisible to direct analysis.^30,31^ Even when synthesized, they rapidly degrade prior to analysis. Therefore, we designed an indirect method to address the prolyl-CoA problem. LC-MS analysis revealed that proline caused the most significant decrease in CoASH peak area (AUC) in comparison with boiled OroD control (Figures 5A and S16). In contrast, incubation with pyrrole-2-carboxylic acid resulted in a smaller decrease of CoASH, and the corresponding pyrrolyl-CoA was not detected by LC-MS although the standard was easily identified. These results suggested that OroD preferentially processes proline and that pyrrolyl-CoA formation was below the detection limit.

**Figure 5:**
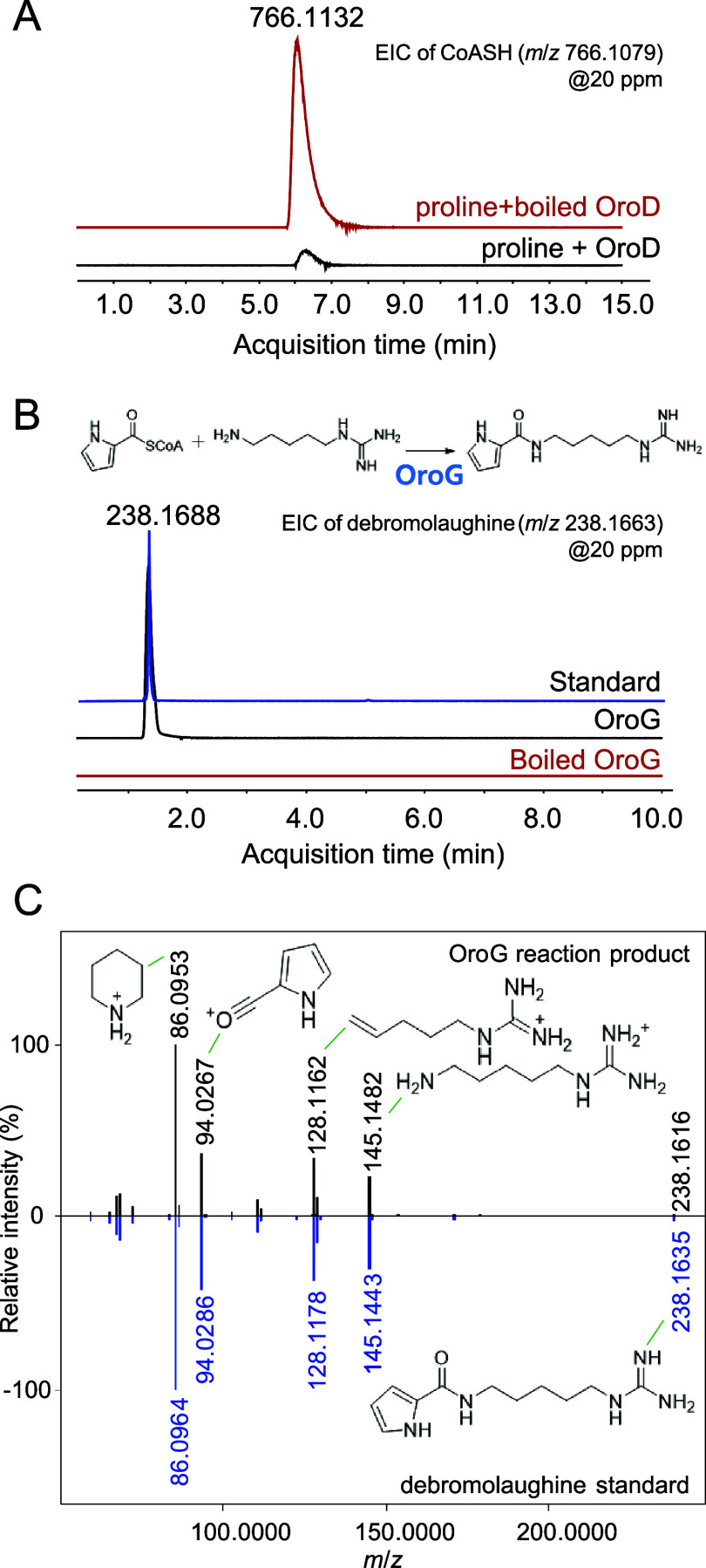
LC-MS analysis of OroD and OroG enzyme reactions. (A) EICs of remaining CoASH in OroD in vitro assay. Red trace is negative control using boiled OroD. (B) EICs from the OroG in vitro assay. The expected ligation product with *m*/*z* 238.1688 was observed (black trace). (C) MS^2^ mirror plot spectrum of OroG reaction product with debromolaughine standard.

Since prolyl-CoA is not stable, purified OroG was incubated with synthetic pyrrolyl-CoA (Figures S32–S39) and homoagmatine for 2 h. The observed product, debromolaughine, was identified by comparison of its LC-MS chromatogram with that of a synthetic standard (Figures 5B and S40–S45). In addition, MS^2^ spectral analysis revealed a pyrrole-derived oxonium ion (*m*/*z* 94.0267), which together with characteristic homoagmatine fragment ions (*m*/*z* 86.0953, 128.1162, and 145.1482), confirmed the covalent linkage (Figure 5C). We further aimed to determine whether other CoA thioesters could serve as carboxylate donors. Incubation with benzoyl-CoA produced the expected product, albeit in low yield, but acetyl-CoA was not a substrate (Figure S17A–C). To examine amine specificity, 1-(3-aminopropyl)­imidazole, agmatine, and lysine were incubated with OroG and pyrrolyl-CoA. The first two were substrates, while lysine was not (Figures S17D–F and S18).

To clarify the reaction order and substrate preferences, homoagmatine was incubated with both OroD and OroG, with either proline or pyrrole-2-carboxylic acid. Although both acid substrates decreased after incubation, the expected ligation products were not detected (Figure S19). Kinetic analysis of OroG under homoagmatine saturated conditions revealed a *K*_m_ of 200 μM for pyrrolyl-CoA (Figure S20). Since we estimated that total pyrrolyl-CoA formation was <20 μM over the time course of the OroD + OroG assay (based upon disappearance of the substrate), this explains the lack of observed product. In addition, given that pyrrolyl-CoA was ligated by OroG, we speculate that prolyl-CoA produced by OroD may require prior oxidation to become a suitable substrate for ligation. Overall, OroD and OroG support the identification of the *oro* bundle, although they do not exactly define the substrates that are ligated.

### OroF, an Unusual FMO Pyrrole Dibrominase

OroF lacks similarity with other FMO-like, FAD-dependent brominases^32^ based upon BLAST or AlphaFold3 ^29^ analyses. Instead, OroF belongs to the endoplasmic reticulum flavoprotein associated with degradation- (ERFAD)-like family of FMO proteins,^33^ distributed throughout the animal kingdom. Nonetheless, when OroF was incubated with pyrrole-2-carboxylic acid, FAD, NADPH, and KBr, with or without *E. coli* flavin reductase SsuE,^34^ both mono- and dibromopyrrole-2-carboxylic acid were detected (Figures 6A1 and S21).

**Figure 6:**
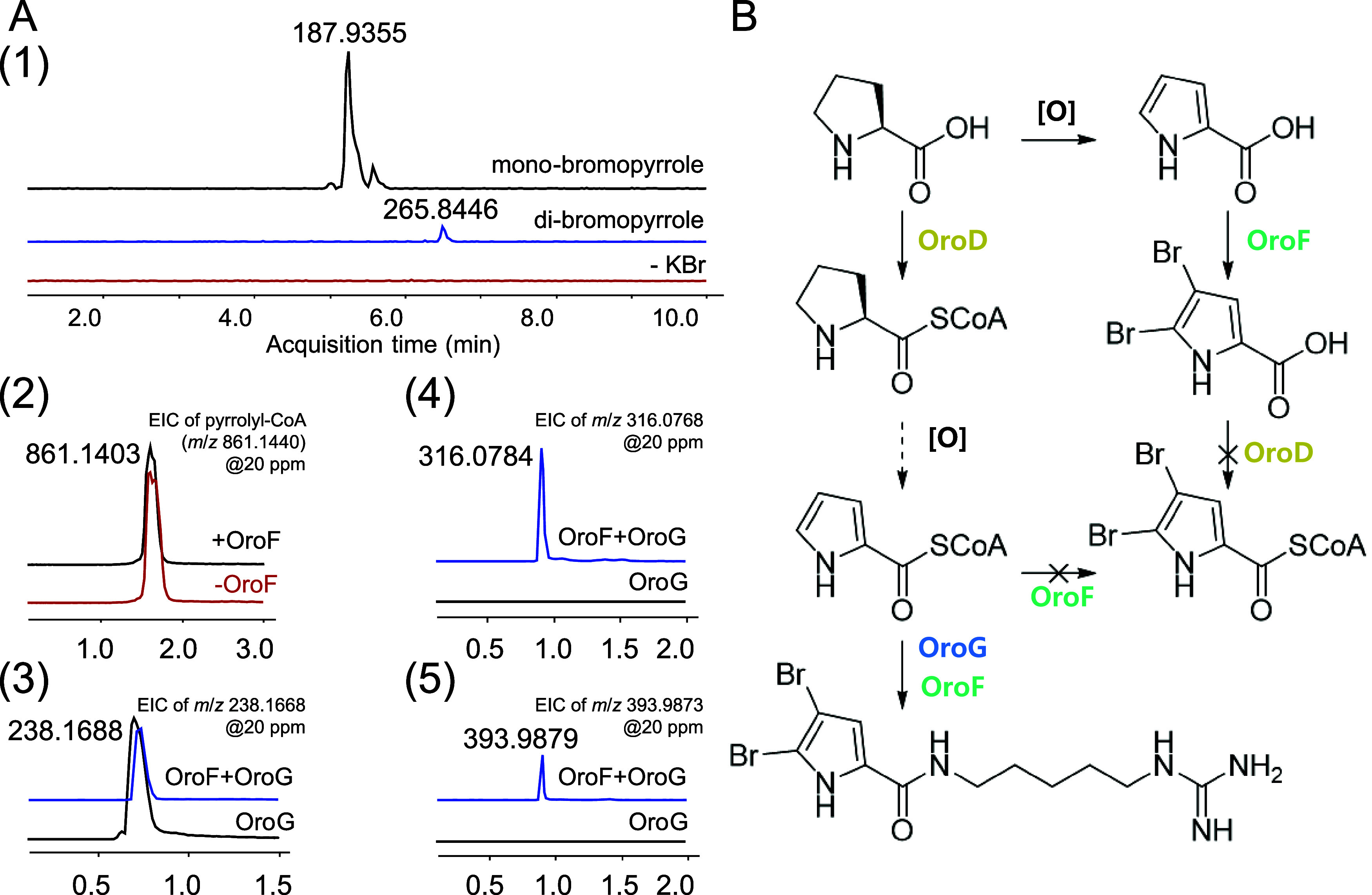
LC-MS analysis of OroF enzyme reaction and timing of bromination. (A) EICs of the OroF in vitro assay; (1) OroF with pyrrole-2-carboxylic acid. (2) OroF with pyrrolyl-CoA. (3) EICs of ligation product, (4) monobrominated, (5) dibrominated ligation product in OroF + OroG assay. (B) Timing of bromination. Based on biochemical results, bromination occurs after amide bond formation.

Our work with OroD and OroG demonstrated amide bond formation, but the timing of bromination was not clear. Since the brominated metabolite was not a substrate for OroD (Figure S16), we aimed to determine whether bromination might occur on the CoA ester or on an amide product of OroG. Synthetic pyrrolyl-CoA was incubated with OroF, but even after 18 h only the starting material was observed (Figures 6A2 and S22). Moreover, the AUC of pyrrolyl-CoA showed no significant difference compared to the boiled-enzyme control, confirming that OroF does not act on pyrrolyl-CoA.

Synthetic pyrrolyl-CoA and homoagmatine were incubated with OroF and OroG in the presence of SsuE, FAD, NADPH. In addition to the expected ligation product (*m*/*z* 238.1688), mono- and dibromo-ligation products were detected (Figure 6A3–5), both in relatively high conversion in comparison to what was previously observed for the free pyrrole acid. The structures were confirmed by diagnostic MS^2^ fragments (Figure S23). Notably, this one-pot enzyme reaction yielded a compound identical to the sponge natural product laughine or its bromine regioisomer.^35^ Furthermore, incubation of synthetic debromolaughine with OroF alone produced same mono- and dibrominated products, as detected by LC-MS. (Figure S24). OroF also brominated synthetic *N*-(2-phenylethyl)-1*H*-pyrrole-2-carboxamide, in which the imidazole moiety on the right side was replaced with a benzene ring (Figure S25). No chlorinated products were detected for either pyrrole-2-carboxylic acid and debromolaughine when KCl was used instead of KBr (Figures S21 and S24). Thus, it is likely that bromination occurs after OroG-catalyzed amide bond formation **(** Figure 6B).

OroF shares 43% amino acid sequence identity with human ERFAD (NP_001095841.1),^10^ which facilitates dislocation of certain endoplasmic reticulum-associated degradation (ERAD) substrates to the cytosol, potentially via a redox-based mechanism. Both OroF and human ERFAD have an N-terminal ER signal peptide, conserved FAD/NAD-binding domain, and a C-terminal domain of unknown function. However, OroF lacks the canonical KEEL/KDEL motif required for endoplasmic reticulum retention, suggesting possible secretion or divergent subcellular localization.^36^ At least two versions of ERFAD-like genes were detected in PIA-producing sponges, while only one was found non-PIA producers, which based upon the phylogenetic tree suggested that duplication and divergence within an oroidin-producing ancestor was responsible for the halogenase. Structural modeling using AlphaFold3 followed by comparative analysis yielded a TM-score of 0.85 between OroF and human ERFAD, indicating high overall fold similarity (Figure S52 ). In contrast, OroF shows no structural similarity with known FAD-dependent halogenases and lacks the conserved halide-binding motifs characteristic of this enzyme class, suggesting a novel catalytic mechanism that remains to be elucidated.

## Discussion

Our laboratories and others have collectively spent decades searching for the biosynthetic proteins that make alkaloids in animals. During that process, we have made and bioinformatically tested many different hypotheses about how such alkaloids might be formed. However, in previous cases we failed not because we did not find such candidate genes, but instead because we found far too many. The enzymes are classically animal in origin, falling into animal clades (i.e., they are not horizontally transferred from microbes) (Figure S53), and are thus presumed not to occur in BGCs. The relatively recent definition of several animal BGCs not originating from horizontal transfer caused us to rethink this problem.^9^ Ultimately, we designed SynBGC, revealing many different BGCs in animals. Not all animal pathways are found in clusters or bundles. There are many counterexamples, including the nemamide biosynthetic pathway from nematodes,^37^ which is distributed across chromosomes, and the animal fatty acid synthase-like polyketide synthases (AFPKs) in molluscs,^38^ which are so far not clustered with genes for modifying enzymes. Nonetheless, the identification of gene bundles in animals was transformative because candidate bundles of potential biosynthetic pathway genes were immediately apparent, leading to the identification of the oroidin pathway *oro* and thus solving a longstanding problem in the field.

*oro* proteins were expressed and characterized, demonstrating that the *oro* BGC was correctly identified and narrowing down possible biosynthetic pathways. We defined the first dedicated steps in the pathway: formation of prolyl-CoA on the carboxyl side, and synthesis of homoagmatine on the amino side. Ligation of prolyl-CoA (or possibly pyrrolyl-CoA) to homoagmatine or a derivative thereof is followed by bromination of the resulting pyrrole (Figure 7). Timing of other oxidation steps was not defined in this study, as the homologous CYP reductase has so far been difficult to express, and further work is needed to find a partner reductase.

**Figure 7:**
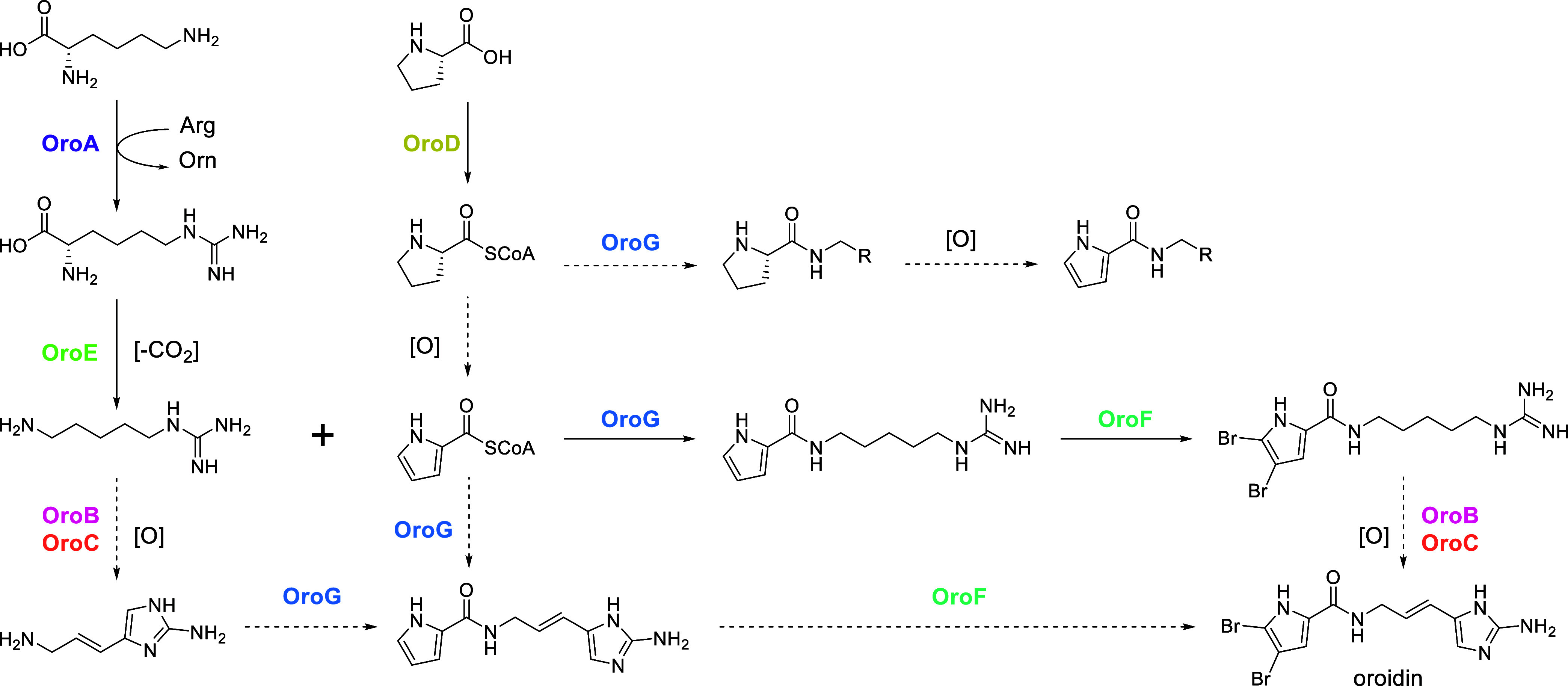
Putative biosynthetic pathway for oroidin. Solid lines indicate steps experimentally confirmed in this work, whereas dashed lines represent hypothetical steps inferred from genes within the *oro* BGC.

Several of these enzymes have immediate applicability to unmet biochemical needs or afford enzymological surprises worth pursuing. The high selectivity of OroE for homoagmatine production suggests that it will have utility in biotechnology and in biological studies to better understand the physiological roles of amidino compounds in nature.^39^ OroF provides an additional, unexpected class of halogenase that is related to ERFAD,^10^ an essential enzyme but for which no enzymatic reaction has been characterized. Understanding the novel enzymology of OroF is therefore a high priority for our further research program. The pathway itself is also of immediate biotechnological relevance.

The natural role of PIAs is to prevent predation of otherwise sedentary and defenseless sponges in the oceans.^40,41^ To do so, PIAs including oroidin block signaling through voltage gated ion channels, including sodium and calcium channels.^42,43^ Given that these channels have nine and 10 subtypes in humans, respectively, the pharmacological potential of the PIAs is not completely elucidated. Other potential applications of PIAs have also been reported.^44–48^ Synthetic methods have afforded analogs with enhanced bioactivity.^49^ Furthermore, PIAs display remarkable structural diversity, arising from various modes of dimerization as well as intra-, intermolecular cyclization. Currently, supply of PIAs is based upon chemical synthesis, and the addition of key enzymes that are immediately and practically useful will provide critical additional tools.

Finally, SynBGC reveals both classical BGCs and many unexpected bundles of biosynthetic genes across the animal kingdom. The existence of such unexpectedly clustered genes is curious and suggests a fundamental biological mechanism in animals that is unexplored. Further, the agnostic application of such a tool, which assumes no a priori biosynthetic steps, is useful in otherwise unexplored biosynthetic pathway types such as those involved in animal alkaloid biosynthesis and other animal natural products. Other methods sometimes uncover some of these pathways. Retrospectively, using plantiSMASH^50^ we could discover portions of *oro*, but *oroF* or *oroG* were not identified because these genes lie outside the densely colocalized core of the cluster, with the exact gene missing depending on the species. By contrast, SynBGC immediately identified the complete *oro* cluster in all genomes, derisking the expensive downstream gene synthesis and expression required for pathway validation. In addition, plantiSMASH could not find other animal pathways or features such as the newly observed glycosylated sterol or creatine pathways or the known NRPS or terpene pathways, showing the power of an agnostic, comparative tool to define metabolic relationships across the tree of life.

In summary, our current work unveils unexpected metabolic relationships in animal genomes and provides a new tool to rapidly and agnostically uncover those relationships. We describe biosynthesis of animal alkaloids and open the door to the diverse and novel alkaloid natural product families, which may ultimately rival those from the more historically used and better understood plants.

## Supplementary Material



## Data Availability

SynBGC was deposited in GitHub (https://github.com/linzhenjian/SynBGC)

## Notes

Materials and Methods, Gene banks numbers for sequences, full sequence of expressed proteins, supporting tables, figures, and spectroscopic data (PDF)

## Abbreviations

AFPK: animal fatty acid synthase-like polyketide synthases
ATP: adenosine triphosphate
AUC: area under curve
BGC: biosynthetic gene cluster
BLAST: basic local alignment search tool
CoA or CoASH: coenzyme A
CYP: cytochrome P450
EIC: extracted ion chromatogram
ER: endoplasmic reticulum
ERAD: endoplasmic reticulum-associated degradation
ERFAD: endoplasmic reticulum flavoprotein associated with degradation
FAD: flavin adenine dinucleotide
FMO: flavin-dependent oxygenase
*k*_cat_: catalytic rate constant (turnover number)
*K*_m_: Michaelis constant
LC: liquid chromatography
MS: mass spectrometry
MS^2^ (MS/MS): tandem mass spectrometry
MIBiG: minimum information about a biosynthetic gene cluster
NAD/NAD­(P): nicotinamide adenine dinucleotide/nicotinamide adenine dinucleotide phosphate
PIA: pyrrole-imidazole alkaloid
PLP: pyridoxal-5′-phosphate
SNAC: *N*-acetyl cysteamine
TIC: total ion chromatogram
TM: template modeling
*V*_max_: maximum rate of reaction
