# Supplementary material for: An Alkaloid Biosynthetic Gene Bundle in Animals
Source: J Am Chem Soc. 2026 Jan 8;148(2):2580–9. doi: 10.1021/jacs.5c18350 (PMC12833800; doi:10.1021/jacs.5c18350)
Supplement: Supplementary file 1 [file ja5c18350_si_001.pdf]

An alkaloid biosynthetic gene bundle in animals

*Jun Gu Kim[1], Zhenjian Lin[1], Vinayak Agarwal[2,3], and Eric W. Schmidt[*1]*

[1]Department of Medicinal Chemistry, University of Utah, Salt Lake City, Utah 84112, USA

[2]School of Chemistry and Biochemistry, Georgia Institute of Technology, Atlanta, GA 30332, USA

[3]School of Biological Sciences, Georgia Institute of Technology, Atlanta, GA 30332, USA

[*]ews1@utah.edu

Materials and Methods

Genomic and transcriptomic datasets employed. Genomes used in this study are listed in **Tables S1 and S2**.

SynBGC overview. The overall process for discovering biosynthetic gene bundles is shown in **Figure S1**, and the SynBGC package is available through https://github.com/linzhenjian/SynBGC. The algorithm has three steps. 1) Shown in **Figure S1A panel 1**, the MIBiG database is mined for all biosynthetic proteins, which are converted into a model using InterProScan. 2) Shown in **Figure S1A panel 2**, relevant genomes are gathered, searched, and filtered to make lists of biosynthetic genes from each genome. 3) The key steps take place in **Figure S1A panel 3**, in which MCScanX is used to find bundles of

biosynthetic gene blocks and to extend them to find relationships over longer portions of the genome. The steps from **panel 3** are further elaborated in **Figure S2**. Finally, BigSCAPE is used to cluster and visualize related bundles of biosynthetic genes.

Figure S1A panel 1: enriching biosynthetic genes. Selected functional classifications of biosynthetic-related proteins from the MIBiG[1] database were used as a reference to identify and enrich biosynthetic-related genes in animal genomes. We extracted 22,391 manually annotated biosynthetic protein sequences from the MIBiG database and then re-annotated them using InterProScan to assign functional classifications based on conserved protein domains and families. To ensure accuracy, the resulting functional annotations were manually examined to remove overly generic classifications not relevant to secondary metabolism. This resulted in a refined list of 2,169 distinct biosynthesis-related functional classifications, which were subsequently used as markers to filter out non-biosynthesis related genes and enrich putative biosynthetic genes in animal genomes.

Figure S1A panel 2: identifying lists of biosynthetic genes in selected genomes. Genome assemblies were annotated using online AUGUSTUS[2] pipeline (https://bioinf.uni-greifswald.de/webaugustus/). Protein sequences were annotated using InterProScan v5.72-103.0[3] against all available databases and assigned functional classifications. The enriched biosynthetic protein sequences obtained as shown in **panel 1** were then compared between genomes using the BLASTp[4] algorithm with the output format: "6 qseqid sseqid pident length mismatch gapopen qstart qend sstart send evalue bitscore". For each genome, GFF files were

extracted from the corresponding GenBank assembly files, containing only the putative biosynthetic genes.

Figure S1A panel 3: creating, comparing, and visualizing biosynthetic gene bundles. Syntenic gene blocks were identified using MCScanX[5] with the following parameters: -e 1 -a -s 3 -m 10. The blocks were further analyzed as described in the following sections on SynBGC, which are also illustrated in **Figure S2**.

SynBGC: segmentation of large syntenic gene blocks. In closely related species, very long syntenic blocks can be detected, sometimes spanning nearly a whole chromosome and containing over 40 putative biosynthetic genes. To obtain an accurate gene cluster comparison, we applied a two-tiered decision framework based on gene count and gap size metrics (**Figure S2**). Each syntenic gene list was first evaluated for total gene count. Lists containing four or more biosynthetic genes were further processed into colinear blocks (referred to as "segmentation"), while lists with fewer than four genes were excluded from segmentation and were instead diverted to a decision tree. In that decision tree, the total number of non-biosynthetic genes between biosynthetic genes had to be less than 14 in order for those genes to be kept. Lists with four or more genes were first mapped back to their original biosynthetic gene order. The number of segments for a series of tested gap sizes (1 to 10) was evaluated for each syntenic gene list. The median gap step size between the last maximum number of segments and the first minimum number of segments was chosen as a dynamic threshold to identify breakpoints in the syntenic gene list. We next applied an additional refinement step using genome-wide gap analysis. Adjacent gene pairs were evaluated according to their

original genomic positions, and the median genome gap was used as a threshold, with a minimum value of 20 and a maximum value of 50. Gaps exceeding this threshold were considered significant and used to identify further split positions. These genome-derived breakpoints were combined with biosynthetic gap positions to finalize segmentation.

Segmented syntenic gene block similarity assessment. While MCScanX[5] effectively detects conserved gene order, not all biosynthetic genes involved in a given pathway are perfectly colinear across species. To account for this, we extracted not only the syntenic biosynthetic genes identified by MCScanX, but also the neighboring putative biosynthetic genes within the surrounding syntenic regions. These expanded gene sets were used to generate GenBank files, which were subsequently analyzed with BiG-SCAPE-1.1.5[6] (--mix --no_classify --cutoffs 0.1 0.2 0.3 0.4 0.5 0.6 0.7 0.8 --mode global) to assess gene cluster similarity and identify conserved biosynthetic modules across species. The codes can be found in the SynBGC package. The input and output data for oroidin BGC mining using SynBGC was deposited in figshare (https://figshare.com/s/45eae1088a83d1ef38ad).

BGC boundary determination. BGC boundaries were determined by integrating synteny comparisons across multiple genomes. Because each genome was compared against all others in the dataset, the same genomic region could be identified multiple times, leading to redundant syntenic segments. These duplicates were removed using the BiG-SCAPE network output, retaining only one out of a group of segment pairs with a Raw Distance ≤ 0.1. For each resulting gene cluster family (GCF), all BGCs were aligned using clinker to identify the

maximal set of shared genes as well as genes uniquely present in individual BGCs. The final gene set representing each BGC was then mapped back onto the original, unsegmented syntenic gene list to verify boundary completeness and recover any genes that might have been missed in earlier segmentation steps.

SynBGC validation. SynBGC was used to find bursatellin-oxazinin clusters[7] in six out of seven bivalve genomes (**Table S1 and Figure S3**). These BGCs formed a distinct group in BiG-SCAPE network analysis and remain clearly separated from unrelated gene clusters, even at a stringent similarity threshold of 0.8, underscoring their coherence and specificity (**Figure S4**). The clusters are quite variable in organization and in the presence of biosynthetic genes, reflecting the flexibility of SynBGC. Similarly, two terpenoid gene clusters were detected from two soft coral species known to produce terpenes, but not from one from which terpenes have not been previously reported (**Figures S3 and S5**).

Gene cloning. All primers used in this study (**Table S3**) were synthesized by the DNA/Peptide Synthesis Core Facility at the University of Utah. A gene fragment encoding OroG was purchased from Twist Bioscience. All other target genes were amplified from complementary DNA (cDNA) synthesized from a previously reported RNA sample of *A. corrugata*[8] using the SuperScript™ IV First-Strand Synthesis System (Invitrogen, Thermo Fisher Scientific), following the manufacturer's instructions. Full-length, wild-type sequences were used in all cases, except that OroA, OroE, and OroF contained N-terminal regions that were truncated (**Table S4**). OroF was predicted by SignalP[9] to contain an N-terminal signal peptide and the same region was identified to be transmembrane by DeepTMHMM,[10] suggesting that it may hinder soluble protein expression. To remove this region, a forward primer downstream of the predicted domain was used

for fragment amplification. In a similar manner, the N-terminal unstructured domains of OroA and OroE, predicted by BlastP and AlphaFold3,[11] were also truncated using the same approach (**Figure S9**).

Both PCR-amplified and synthetic gene fragments were cloned into the pET28b(+) expression vector in-frame with an N-terminal His_6-tag, using an in-house Gibson assembly master mix. The resulting constructs were transformed into chemically competent *Escherichia coli* DH10β cells. Transformed colonies were grown overnight at 30°C, and plasmids were extracted using the QIAprep 2.0 Spin Miniprep Kit (Qiagen) according to the manufacturer's protocol. Construct integrity and sequence accuracy were verified by whole-plasmid sequencing (Plasmidsaurus).

Protein expression and purification. Plasmids were transformed into *E. coli* Bl21(DE3) and plated on LB-agar containing kanamycin (50 μg/ml). After overnight incubation at 30°C, two single colonies were picked and separately inoculated into LB medium (10 mL) supplemented with kanamycin (50 μg/ml). The seed cultures were grown overnight at 30 °C with shaking at 180 rpm and subsequently transferred into 1 liter of 2xYT medium containing 50 μg/ml kanamycin, in 2.8 L Fernbach flasks. Cultures were grown until the OD_{600} reached 0.4–0.8, then cooled to 16 °C for 1 hour prior to induction with 0.1 mM isopropyl β-D-1-thiogalactopyranoside (IPTG). Following induction, cells were incubated at 16 °C for 18 hours with shaking at 200 rpm. Cells were harvested by centrifugation at 3,470 x *g* for 15 minutes and resuspended in lysis buffer (50 mM Tris-HCl, 200 mM NaCl, 5% glycerol, 10 mM imidazole, pH = 7.5) at a ratio of 5 mL of buffer per gram of wet cell pellet. Lysozyme was

added to the suspension at a final concentration of 0.6 mg/mL and the cell suspension was stirred at 4 °C for 1 hour. Cells were sonicated on ice using five 2 minute cycles at 300 W, with 1 second pulses followed by 3 seconds of rest. The lysate was subsequently incubated for 15 minutes with deoxyribonuclease I (20 μg/ml) and 20 mM $MgCl_2$. The cell lysate was centrifuged at 21,728 × *g* for 45 minutes at 4 °C, and the supernatant was incubated with Ni-NTA resin (Qiagen) at 1:5 (resin:lysate, v/v) for 50 minutes at 4 °C. The resin was transferred to a gravity flow column and washed sequentially with 8 column volumes (CV) of lysis buffer, followed by 6 CV of wash buffer (1 M NaCl, 30 mM imidazole, pH 8.0). The His-tagged protein was eluted with 15 mL each of elution buffer 1 (1M NaCl, 200 mM imidazole, pH = 8.0) and elution buffer 2 (1M NaCl, 500 mM imidazole, pH = 8.0), and the fractions were analyzed by SDS-PAGE (**Figure S8**). Fractions containing OroD, OroF, and OroG were buffer-exchanged to storage buffer (20 mM Tris-HCl, 300 mM NaCl, 10% glycerol, pH 8.0) and concentrated to final volume of 1 mL. Both OroA and OroE co-eluted with proteins of approximately 70 kDa, which were assumed to be *E. coli*-derived chaperones. For further purification, Ni-NTA purified fractions were buffer-exchanged into the starting buffer for anion-exchange chromatography (buffer A, 20 mM Tris-HCl, pH 7.5), and concentrated to 500 μL using an Amicon Ultra-15 centrifugal filter unit with a 30 kDa molecular weight cutoff (EMD MilliporeSigma). Concentrated fractions were applied to a HiPrep QFF 16/10 column (GE Healthcare) and purified using an ÄKTA Start FPLC. After washing with 5 CV of buffer A to remove unbound proteins, bound proteins were eluted with linear gradient from buffer A to

buffer B (20 mM Tris-HCl, 1M NaCl, pH 7.5) over 20 CV, followed by an additional wash with 5 CV of buffer B. Fractions were analyzed by SDS-PAGE, and those containing target protein were buffer-exchanged into storage buffer, and concentrated to final volume of 500 μL.

OroA was successively purified by anion-exchange chromatography (AEX), whereas OroE was still impure. Therefore, OroE-containing fractions were pooled, buffer-exchanged to buffer C (10 mM sodium phosphate buffer with 140 mM NaCl, pH 7.4), and subjected to size-exclusion chromatography (SEC) on a Superose Increase 10/300 GL column (GE Healthcare). SEC was performed on ÄKTA Go FPLC system with isocratic elution in buffer C for 1.5 CV. Fractions were analyzed by SDS-PAGE, and those containing the desired protein were buffer-exchange into storage buffer and concentrated to 500 μL.

For the expression and purification of *E. coli* flavin reductase SsuE, an in-house glycerol stock was streaked onto an LB agar plate containing kanamycin (50 μg/mL), and a single colony was used to initiate protein production. The overall procedure was the same as that used for other proteins, except that the eluted fraction was dialyzed against buffer containing 25 mM HEPES and 500 mM NaCl.

Protein concentrations were estimated by NanoDrop spectrophotometry (Thermo Fisher Scientific) using extinction coefficients calculated by the ProtParam tool (ExPASy). The protein samples were aliquoted and flash-frozen in liquid nitrogen for storage in -80°C.

UPLC-HRMS and MS/MS analysis. For the analysis of enzyme reactions in this study, high-resolution UPLC-MS and MS/MS analysis were performed using a Waters Xevo G2-XS Q-Tof mass spectrometer coupled to Acquity UHPLC system. Unless otherwise noted,

chromatographic separation was performed using mobile phases consisting of water with 0.1% formic acid (solvent A) and acetonitrile with 0.1% formic acid (solvent B). The following UPLC methods were employed depending on the enzyme reaction analyzed:

Method 1:

Waters Acquity UPLC BEH HILIC column (1.7 µm, 2.1 × 100 mm); flow rate: 0.4 mL/min; gradient: 0–1.0 min, 95% B; 1.0–10.0 min, 95–50% B; 10.0–11.5 min, 50% B; 11.5–13.0 min, 95% B.; column temperature: 35 °C.

Method 2:

Waters Acquity UPLC CSH C18 column (1.7 µm, 2.1 × 50 mm); flow rate: 0.3 mL/min; gradient: 0–2.0 min, 5% B; 2.0–10.0 min, 5–100% B; 10.0–13.0 min, 100% B; 13.0–15.0 min, 5% B.; column temperature: 35 °C.

Method 3:

Waters Acquity UPLC CSH C18 column (1.7 µm, 2.1 × 100 mm); flow rate: 0.3 mL/min; gradient: 0–2.0 min, 20% B; 2.0–30.0 min, 20–65% B; 30.0–32.5 min, 100% B; 32.5–35.0 min, 20% B.; column temperature: 35 °C.

Method 4:

Waters Acquity UPLC CSH C18 column (1.7 µm, 2.1 x 50 mm); mobile phase: A–20 mM ammonium formate in water, B–acetonitrile; flow rate: 0.3 mL/min; gradient: 0–2.0 min, 5% B; 2.0–10.0 min, 5–50% B; 10.0–13.0 min, 100% B; 13.0–15.0 min, 5% B.; column temperature: 35 °C.

Negative ionization mode was used to detect mono- and di-bromopyrrole-2-carboxylic acid in OroF assay and CoASH in OroD assay, whereas positive ionization mode was used for all other reactions.

MS/MS spectra were acquired by using a data-dependent acquisition (DDA) mode, consisting of an MS^1 survey scan followed by MS/MS scan of three most intense precursor ions detected in the MS^1 scan. Collision-induced fragmentation was performed using a stepped collision energy ramp ranging from 20 to 80V. The raw data files were converted to mzXML format using MSConvert (ProteoWizard, version 3.0.22288) and subsequently processed and visualized with Mzmine 3.9.0 software (https://mzmine.github.io).

Enzyme assays.

OroA (amidinotransferase). The reaction was carried out in a total volume of 50 µL, containing 200 µM lysine or 1,5-diaminopentane, 2 mM arginine, 10 µM OroA, and 500 µM $MgCl_2$ in 50 mM Tris-HCl buffer (pH 8.0). The mixtures were incubated at 30 °C for 2 hours. Negative control reactions were prepared under identical conditions using heat-denatured enzyme. After incubation, reactions were quenched by the addition of 100 µL of acetonitrile, followed by centrifugation at 15,871× *g* for 10 minutes. The resulting supernatants were collected and analyzed by LC-MS (Method 1). For derivatization experiments,[12] 100 µL of L-FDLA (1% in acetone) and 30 µL of 1 M $NaHCO_3$ were added to a separate reaction mixture that had been quenched by heating at 98 °C for 5 minutes. The mixtures were then incubated at 45 °C for 1 hour. Following derivatization, the reaction was neutralized by the addition of 1 N HCl, centrifuged to remove precipitates, and the supernatant was analyzed by LC-MS using Method 3. Amino acid standards

involved in the reaction—arginine, ornithine, lysine, and homoarginine—were derivatized in the same manner and analyzed alongside the reaction samples for comparison.

OroE (decarboxylase). Reactions (50 μL) were conducted in 50 mM Tris-HCl buffer (pH 7.5), containing 200 μM of one of the following substrates: ornithine, lysine, arginine, or homoarginine; 10 μM OroE; 100 μM pyridoxal-5′-phosphate; and 2.5 mM dithiothreitol (DTT). Enzyme denatured by boiling was used in parallel as a negative control. Reactions were incubated at 37 °C for 2 hours and quenched by adding 100 μL of acetonitrile. After centrifugation at 15,871 × *g* for 10 minutes, the supernatants were analyzed by LC-MS using Method 1.

OroF (flavin monooxygenase). Reactions (50 μL) were performed in 25 mM HEPES buffer (pH 7.5) containing 200 μM pyrrole-2-carboxylic acid, 10 μM OroF, 100 μM FAD, 5 mM NADPH, and 50 mM KBr. Reactions were incubated at 37 °C for 20 hours, either in the presence or absence of 10 μM SsuE. Following incubation, 1 mL of ethyl acetate was added to extract the reaction products.[13] The mixture was centrifuged at 15,871× *g* for 10 minutes, and the organic (upper) layer was collected, evaporated to dryness, and reconstituted in 100 μL of acetonitrile. Samples were analyzed by LC-MS using Method 2 with negative ionization method. As negative controls, separate reactions either lacking KBr or using heat-denatured enzyme were performed to ensure product formation was enzyme- and bromide-dependent.

Reactions using 200 μM of *N*-(2-phenylethyl)-1H-pyrrole-2-carboxamide as the substrate were carried out under the same conditions as described above, but were incubated for 2 hours and analyzed using positive ionization mode.

For reactions containing 200 μM pyrrolyl-CoA or debromolaughine as the substrate, 50 μL of methanol was directly added to quench the reaction after 2 hours of incubation at 37 °C. The mixture was then centrifuged at 15,871 × *g* for 10 minutes, and the supernatants were analyzed by LC-MS using Method 1 (debromolaughine) or Method 4 (pyrrolyl-CoA) with positive ionization mode.

OroD (CoA ligase). Reactions (50 μL) were performed in 50 mM HEPES buffer (pH 8.0) containing 1 mM of proline, pyrrole-2-carboxylic acid, or 4,5-dibromo-1H-pyrrole-2-carboxylic acid, 1 mM ATP, 10 μM OroD, 1 mM CoASH, 2 mM TCEP, 10 mM $MgCl_2$. Reactions were incubated at 37 °C for 2 hours and then quenched with an equal volume of methanol containing Leu-enkephalin as an internal standard (final concentration: 0.4 μg/ml) and centrifuged at 15,871 × *g* for 10 minutes. The resulting supernatant was analyzed by LC-MS using Methods 2 and 4.

OroG (acyltransferase). 100 μM pyrrolyl-CoA, 100 μM homoagmatine, and 10 μM OroG were incubated in 50 mM sodium phosphate buffer (pH 7.0) containing 500 mM NaCl and 2 mM EDTA (total volume of 50 μL). Following incubation at 37 °C for 2 hours, the reactions were quenched by adding 50 μL of acetonitrile. After centrifugation at 15,871 × *g* for 10 minutes, the supernatants were analyzed by LC-MS using Method 2. Heat-denatured enzyme was used as a negative control.

Additionally, 100 μM benzoyl-CoA and acetyl-CoA were tested with 100 μM homoagmatine under the same conditions. Commercially available amine partner, 1-(3-aminopropyl)imidazole (TCI America, Portland, OR, USA), agmatine dihydrochloride (Fisher

Scientific, Waltham, MA, USA) and lysine (Sigma-Aldrich, St. Louis, MO, USA), were each tested at 100 μM with 100 μM pyrrolyl-CoA. The reaction with 1-(3-aminopropyl)imidazole was analyzed using Method 1.

One-pot reaction of OroD and OroG. Reactions were carried out with 100 μM of either proline or pyrrole-2-carboxylic acid, 100 μM homoagmatine, 1mM ATP, 1mM CoASH, 2mM TCEP, 10 mM $MgCl_2$, 10 μM OroD, and 10 μM OroG in 25 mM sodium phosphate buffer (pH 7.0) containing 100 mM NaCl. The 50 μL reaction mixtures were incubated at 37 °C for 2 hours and quenched with an equal volume of methanol. After centrifugation at 15,871 × *g* for 10 minutes, the supernatants were analyzed by LC-MS using Method 2.

One-pot reaction of OroF and OrG. Reaction (50 μL) were carried out in 25 mM HEPES buffer (pH 8.0) containing 100 μM pyrrolyl-CoA, 100 μM homoagmatine, 10 μM OroG, 10 μM OroF, 5 μM SsuE, 100 μM FAD, 5 mM NADPH, and 50 mM KBr. Parallel reactions containing OroG only or OroF only under the same condition were performed for the comparison. After incubation at 37 °C for 20 hours, reaction quenched with 50 μL of methanol and centrifuged at 15,871 × *g* for 10 minutes. The supernatants were analyzed by LC-MS using both Method 1 and 2.

Kinetic analysis. Standard calibration curves for homoarginine, homoagmatine, and debromolaughine were generated by triplicate analysis of serial dilutions of the standards using LC-MS Method 1 (**Figures S13A, B and S20A**). To determine the optimal enzyme concentration (initial velocity condition), a range of concentrations of OroA and OroE were incubated with 200 μM of substrate, respectively **(Figures S13C and S13D**). To find the saturation

concentration of homoagmatine for OroG reaction, 100 μM of pyrrolyl-CoA was incubated with a range of homoagmatine concentrations (100 μM – 8 mM) (**Figure S20B**). Reaction mixtures were quenched at designated time points and subsequently analyzed. Kinetic analyses were conducted as below:

OroA with lysine:

Substrate concentrations of lysine ranging from 1 to 800 μM in the presence of 0.25 μM OroA, 1 mM arginine, and 500 μM $MgCl_2$ in 50 mM Tris-Cl buffer (pH 8.0). Reaction time points were set at 5, 10, 15, 20, and 30 minutes and incubated at 30 °C.

OroA with 1,5-diaminopentane:

Substrate concentrations of 1,5-diaminopentane ranging from 250 to 6,400 μM in the presence of 0.25 μM OroA, 1 mM arginine, and 500 μM $MgCl_2$ in 50 mM Tris-Cl buffer (pH 8.0). Reaction time points were set at 10, 30, 60, 120 and 180 minutes and incubated at 30 °C.

OroE with homoarginine:

Substrate concentrations of homoarginine ranging from 1 to 800 μM in 0.5 μM OroE, 100 μM pyridoxal 5′-phosphate, and 2.5 mM DTT in 50 mM Tris-Cl buffer (pH 7.5). Reaction time points were set at 5, 10, 15, and 20 minutes and incubated at 30 °C.

OroG with pyrrolyl-CoA:

Substrate concentrations of pyrrolyl-CoA ranging from 20 to 800 μM in 0.1 μM OroG, 4 mM homoagmatine in 25 mM sodium phosphate buffer (pH 7.0) containing 100 mM NaCl. Reaction time points were set at 15 and 30 minutes and incubated at 30 °C.

At each time point, the reactions were quenched and analyzed by LC-MS. The obtained area under the curve (AUC) values were converted to product concentrations using the standard calibration curve. Michaelis–Menten kinetics were analyzed by nonlinear regression, and kinetic parameters were derived from triplicate AUC values using Prism 9.5 (GraphPad Software, San Diego, CA, USA).

Chemical synthesis.

General considerations. Chemical reagents used in the synthesis were purchased from Fisher Scientific (thiophenol, DIPEA, anhydrous $MgSO_4$), TCI America (1,5-diaminopentane), Chemipex (*N*,*N*'-bis-Boc-*S*-methylisothiourea and 1-hydroxybenzotriazole; HOBt), Sigma-Aldrich (*N*,*N*'-dicyclohexylcarbodiimide; DCC), CoALA bioscience (coenzyme A lithium salt), and Thermo Scientific (phenylethylamine). HPLC-grade solvents (Fisher Scientific) were used for synthesis and purification. Purification was performed using either a Teledyne CombiFlash NextGen 300+ system (Teledyne ISCO) or Ultimate 3000 HPLC system (Thermo Fisher Scientific). NMR spectra were recorded on a Varian Innova 500 MHz NMR spectrometer at 300 K, and chemical shifts were referenced to the residual solvent peaks (δ_H 7.26 and δ_C 77.16 for $CDCl_3$, δ_H 4.66 for D_2O).

Synthesis of homoagmatine. Synthesis of homoagmatine followed a previous protocol.[14] To a stirred solution of 1,5-diaminopentane (0.88 g, 10 equiv) in 4 mL DMF, *N*,*N*'-bis-Boc-*S*-methylisothiourea (0.25 g, 1 equiv) was added dropwise over the course of 1 hour. Upon completion of the addition, the reaction was quenched with 5 mL of water and extracted with a 1:1 mixture of hexane and diethyl ether. The organic layer was dried over anhydrous $MgSO_4$ and

concentrated under reduced pressure. *N,N′*-bis-Boc-homoagmatine was purified using a Teledyne CombiFlash system equipped with a High Performance Gold 15.5 g HP C18 column. The purified intermediate was dissolved in a 1:1 mixture of trifluoroacetic acid (TFA) and dichloromethane (DCM) and stirred at room temperature for 2 hours. The resulting oily product was washed with cold diethyl ether five times to yield homoagmatine. The identity, purity, and structure of the synthetic homoagmatine were confirmed by LC-MS and NMR analysis (**Figures S26-S31**).

homoagmatine: ^{1}H-NMR (500 MHz, D_2O): δ 3.08 (t, 2H), 2.89 (t, 2H), 1.59 (m, 2H), 1.52 (m, 2H) 1.32 (m. 2H); ^{13}C-NMR (125 MHz, D_2O): δ 159.4, 43.5, 41.9, 30.0, 29.0, 25.5; HRESIMS *m/z* 145.1449 $[M+H]^+$ (calcd for $C_6H_{17}N_4$, 145.1448).

Synthesis of pyrrolyl-CoA. Pyrrolyl-CoA was synthesized via thiophenolation following a previous method with modifications.[7, 15] Pyrrole-2-carboxylic acid (20.0 mg, 1 equiv), *N*,*N′*-dicyclohexylcarbodiimide (DCC, 37.1 mg, 1 equiv), and 1-hydroxybenzotriazole (HOBt, 27.6 mg, 1 equiv) were dissolved in 4 mL of anhydrous ethyl acetate and stirred at room temperature. Upon formation of a white precipitate (dicyclohexylurea), thiophenol (23.8 mg, 1.2 equiv) was added, and the reaction mixture was stirred for an additional 22 hours. After completion, the mixture was filtered through a 0.2 µm PTFE syringe filter, washed with 10% aqueous citric acid, and concentrated under reduced pressure. The crude product was reconstituted in acetonitrile and purified by reversed-phase HPLC using a Phenomenex Luna C18 column (10 × 250 mm, 5 µm) with 50:50 isocratic elution of water containing 0.01% TFA and acetonitrile (flow rate = 4.0 mL/min). Lyophilization yielded 7.0 mg of *S*-phenyl-pyrrole-2-carbothioate. The structure and purity were confirmed by LC-MS and NMR analysis (**Figures S32-S36**).[16] Pyrrolyl-CoA was subsequently synthesized via a thiol–thioester exchange reaction. *S*-phenyl-pyrrole-2-carbothioate (2.5 mg) was dissolved in 200 µL of anhydrous tetrahydrofuran (THF) and mixed with an equal

volume of coenzyme A (in 100 mM sodium phosphate buffer, pH 8.5). The reaction was stirred at room temperature for 24 hours, then directly injected into an RP-HPLC system equipped with a Phenomenex Luna C18 column (10 × 250 mm, 5 μm) for purification using a linear gradient from 95:5 (water containing 0.01% TFA : acetonitrile) to 0:100 over 35 min (flow rate = 4.0 mL/min, retention time of pyrrolyl-CoA =10.4 min). Pyrrolyl-CoA (1.7 mg) was obtained after lyophilization, and its structure was confirmed by comparison of the ^{1}H-NMR spectrum with previously reported data (**Figures S37-S39**).[15]

S-phenyl-pyrrole-2-carbothioate: ^{1}H-NMR (500 MHz, $CDCl_3$): δ 9.28 (br s, 1H), 7.53 (m, 2H), 7.45 (m, 3H) 7.15 (m, 1H) 7.04 (m, 1H), 6.34 (m, 1H); ^{13}C-NMR (125 MHz, $CDCl_3$): δ 180.0, 135.3, 129.5, 129.3, 127.0, 124.2, 115.8, 111.1; HRESIMS *m/z* 204.0494 $[M+H]^+$ (calcd for $C_{11}H_{10}NOS$, 204.0478).

pyrrolyl-CoA: ^{1}H-NMR (500 MHz, D_2O): δ 8.67 (s, 1H), 8.36 (s, 1H), 7.16 (s, 1H), 7.03 (d, 1H), 6.29 (m, 1H), 6.20 (d, 1H), 4.62 (s, 1H), 4.29 (m, 2H), 4.05 (s, 1H), 3.89 (m, 1H), 3.61 (m, 1H), 3.47 (t, 2H), 3.42 (t, 2H), 3.13 (t, 2H), 2.46 (t, 2H), 0.96 (s, 3H), 0.83 (s, 3H); HRESIMS *m/z* 861.1463 $[M+H]^+$ (calcd for $C_{26}H_{40}N_8O_{17}P_3S$, 861.1440).

Synthesis of debromolaughine. *N,N*-bis-Boc-homoagmatine, the amine donor, was prepared from the intermediate obtained during homoagmatine synthesis described above. Pyrrole-2-carboxylic acid (20.0 mg, 1 equiv), DCC (40.9 mg, 1.1 equiv), and HOBt (26.8 mg, 1.1 equiv) were dissolved in 4 mL of anhydrous ethyl acetate and stirred at room temperature until the formation of dicyclohexylurea was visibly observed. *N,N*-bis-Boc-homoagmatine (84.6 mg, 1.2 equiv) and DIPEA (46.5 mg, 2.0 equiv) were then added, and the reaction mixture was

stirred for 5 hours at room temperature. The mixture was centrifuged at 15,871 × *g* for 10 min, and the supernatant sequentially washed with 0.2 M citric acid, saturated $NaHCO_3$ solution. The resulting aqueous phase was dried over anhydrous $MgSO_4$. After evaporating the solvent under reduced pressure, the crude product was reconstituted with methanol and purified with RP-HPLC (Phenomenex Luna C18, 10 x 250 mm, 5 μm, 40:60 isocratic elution of water containing 0.01% TFA and acetonitrile, flow rate = 4.0 mL/min). The purified intermediate was then dissolved in the 1:1 mixture of DCM/TFA (4 mL) containing TIPS (50 μL) and stirred for 1 hour at 0 °C. The solution was evaporated under reduced pressure, and the resulting product was washed with cold diethyl ether and lyophilized. The structure and purity of product were analyzed by LC-MS and NMR (**Figures S40-S45**).

debromolaughine: ^{1}H-NMR (500 MHz, D_2O): δ 6.94 (m, 1H), 6.70 (dd, 1H), 6.18 (dd, 1H), 3.22 (t, 2H), 3.05 (t, 2H), 1.49 (m, 4H), 1.28 (m, 2H); ^{13}C NMR (125 MHz, D_2O) δ 165.8, 159.3, 127.6, 125.6, 113.6, 112.1, 43.6, 41.5, 30.8, 30.2, 25.7; HRESIMS *m/z* 238.1662 $[M+H]^+$ (calcd for $C_{11}H_{20}N_5O$, 238.1663).

Synthesis of *N*-(2-phenylethyl)-1*H*-pyrrole-2-carboxamide. Pyrrole-2-carboxylic acid (20.0 mg, 1 equiv), DCC (40.9 mg, 1.1 equiv), and HOBt (26.8 mg, 1.1 equiv) were dissolved in 4 mL of anhydrous ethyl acetate and stirred at room temperature. Upon visible formation of dicyclohexylurea, phenylethylamine (26.2 mg, 1.2 eq) and DIPEA (46.5 mg, 2.0 equiv) were added. After 6 hours, the reaction mixture was centrifuged at 15,871 × *g* for 10 min and the supernatant was collected. An equal volume of 1N HCl (4 mL) was added to the supernatant, vortexed, and the aqueous phase collected. This was then treated with saturated $NaHCO_3$,

vortexed, and resulting supernatant dried over anhydrous $MgSO_4$. The solvent was removed under reduced pressure, and the crude product was purified with RP-HPLC (Phenomenex Luna C18, 10 x 250 mm, 5 μm, 50:50 isocratic elution of water containing 0.01% TFA and acetonitrile, flow rate = 4.0 mL/min). The purified and lyophilized product (7.0 mg) was characterized by LC-MS and NMR to confirm its structure (**Figures S46**-**S51**).

N-(2-phenylethyl)-1*H*-pyrrole-2-carboxamide: ^{1}H-NMR (500 MHz, $CDCl_3$): δ 9.73 (br s, 1H), 7.34 (m, 2H), 7.25 (m, 3H), 6.93 (s, 1H), 6.44 (s, 1H), 6.22 (s, 1H), 5.93 (br s, 1H), 3.69 (q, 2H), 2.91 (t, 2H); ^{13}C-NMR (125 MHz, $CDCl_3$): δ 163.8, 141.5, 131.5, 131.4, 129.2, 124.2, 112.4, 111.2, 43.2, 38.7; HRESIMS *m*/*z* 215.1215 $[M+H]^+$ (calcd for $C_{13}H_{15}N_2O$, 215.1179).

Table S1. Genome assemblies used to test SynBGC.

group	Species	GenBank Number
mollusc	*Pecten maximus*	GCF_902652985.1
	Mytilus edulis	GCF_963676685.1
	Magallana gigas	GCF_963853765.1
	Crassostrea virginica	GCF_002022765.2
	Crassostrea angulata	GCF_025612915.1
	Argopecten irradians	GCF_041381155.1
	Tegillarca granosa	GCA_029721355.1
coral	*Eunicella cavolini*	GCA_965177985.1
	Eunicella verrucosa	GCA_964289765.2
	Muricea muricata	GCA_963855995.1
	Paramuricea clavata	GCA_902702795.2
plant	*Arabidopsis thaliana*	GCF_000001735.4
	Arabidopsis lyrata	GCF_000004255.2
	Arabidopsis arenosa	GCA_905216605.
vertebrate	*Alligator mississippiensis*	GCF_000281125.3
	Alligator sinensis	GCF_000455745.1
	Chiloscyllium plagiosum	GCF_004010195.1
	Chrysemys picta	GCF_000241765.5
	Erpetoichthys calabaricus	GCF_900747795.2
	Ficedula albicollis	GCF_000247815.1

	Homo sapiens	GCF_009914755.1
	Latimeria chalumnae	GCF_037176945.1
	Lethenteron reissneri	GCF_015708825.1
	Meleagris gallopavo	GCF_000146605.3
	Myxine glutinosa	GCF_040869285.1
	Nanorana parkeri	GCF_000935625.1
	Pan troglodytes	GCF_028858775.2
	Pelodiscus sinensis	GCF_000230535.1
	Petromyzon marinus	GCF_048934315.1
	Polypterus senegalus	GCF_016835505.1
	Pristis pectinata	GCF_009764475.1
	Protopterus annectens	GCF_019279795.1
	Python bivittatus	GCF_000186305.1
	Takifugu bimaculatus	GCA_004026145.2
	Tetraodon nigroviridis	GCA_000180735.1
	Thamnophis sirtalis	GCF_001077635.1
	Xenopus tropicalis	GCF_000004195.4

Table S2. GenBank accession numbers for sponge assemblies.

Species	Genome Assembly (GenBank)	Raw Data (SRA/Project)
Axinella damicornis	GCA_963931865.1	ERR12318589

Species	Genome Assembly (GenBank)	Raw Data (SRA/Project)
Axinella polypoides	GCA_964261215.2	ERR2930145
Agelas oroides	GCA_949130485.1	ERR10177763
Agelas tubulata	GCA_964245335.1	ERR14711436
Agelas conifera	GCA_965122385.1	ERR14693505
Cymbastela concentrica	GCA_965112925.1	ERR13947490
Phakellia robusta	GCA_964264905.1	ERR14379135
Axinella corrugata	—	PRJNA1001903

Table S3. Primers used in this study.

Primer name	Sequence	Role
Amidinotransferase_fwd	atggcttcatttcaaggaattgtcc	PCR amplification from cDNA
Amidinotransferase_rev	tcacaagtatgactcaagagtcc	
Amidinotransferase_frag_fwd	catatggctgaagcttgcccggtttcct	Gibson assembly to pET28b
Amidinotransferase_frag_rev	cggatcccgtcacaagtatgactcaagagtcccccct	
Amidinotransferase_vec_fwd	tcatacttgtgacgggatccgaattcgagc	
Amidinotransferase_vec_rev	gcaagcttcagccatatggctgccgc	
Decarboxylase_fwd	atgcttcaaacctggagat	PCR amplification from cDNA
Decarboxylase_rev	ttattgcatctctacttcattcc	
Decarboxylase_frag_fwd	ccatatggctacatctacaagagcattgag	Gibson assembly to pET28b
Decarboxylase_frag_rev	cggatctcattgcatctctacttcattcctg	
Decarboxylase_vec_fwd	gagatgcaatgacgggatccgaattcgagc	
Decarboxylase_vec_rev	cttgtagatgtagccatatggctgccgcg	
FMO_fwd	atgcagaccaccatgaaatcc	PCR amplification from cDNA
FMO_rev	gaccatgaaagcctctggaga	
FMO_frag_fwd	agccatatgggaggagacggaagcacag	Gibson assembly to pET28b
FMO_frag_rev	cggatcccgtcagaccatgaaagcctctggagatcc	
FMO_vec_fwd	ttcatggtctgacgggatccgaattcgagctc	
FMO_vec_rev	gtctcctcccatatggctgccgcgcg	
Acyltransferase_frag_fwd	ttccaaggaatggccggtcgtatgtgc	Gibson assembly to pET28b
Acyltransferase_frag_rev	gctagccatatgttataacttgaaattcttaccgttcag	
Acyltransferase_vec_fwd	tcaagttataacatatggctagcatgactggtgg	
Acyltransferase_vec_rev	accggccattccttggaagtagaggttttcgcc	
CoA synthetase_fwd	atgtttgcgatggcctgc	PCR amplification from cDNA
CoA synthetase_rev	tcattgtgattgcatttccttgtg	
CoA synthetase_frag_fwd	ttccaaggaatgtttgcgatggcctgc	Gibson assembly to pET28b
CoA synthetase_frag_rev	ctagccatatgtcattgtgattgcatttccttgtg	
CoA synthetase_vec_fwd	atcacaatgacatatggctagcatgactggtgg	
CoA synthetase_vec_rev	cgcaaacattccttggaagtagaggttttcgcc	

Table S4. Sequences of Oro proteins used in this study.

Protein	Sequence[1]
OroA	**MASFQGIVRGILRHRRPVIQLRSWSIFGERNASTAAATTSPATE**<br>**QQAQINPE**EACPVSSHNEWDPLEEVIVGRPEGACVPHFSHEVKAII<br>SKEHWDFFEKNSGKPFPAEHVKKAIEEMEEFCRILEHEGVTVRRPE<br>LVDYSKEYSTPDFTATGHISMPRDFIMVIGNEVFESPMAWRSRFFE<br>YRAYRPLVKEYFHRGAKWTTAPKPLMSDELFDYSYPVDNKEEQH<br>RLGLQQKYVTTEFEPCFDAADFLRAGKDIFVQKSQVTNRFGIEWM<br>RRHLGDQYNVHILSFNDSNPMHIDASYTFLEPGLLMENPERRCHQ<br>TNSIFVKAGWKVVDAPRPAIPDSHPLWMTSKWLSMNTLMLGPKR<br>VVVEAGEIPTQKAFEKLGIECIKVPLRFAQSLGGGFHCWTCDVRRR<br>GTLESYL
OroA_N_truncated	EACPVSSHNEWDPLEEVIVGRPEGACVPHFSHEVKAIISKEHWDFF<br>EKNSGKPFPAEHVKKAIEEMEEFCRILEHEGVTVRRPELVDYSKEY<br>STPDFTATGHISMPRDFIMVIGNEVFESPMAWRSRFFEYRAYRPLV<br>KEYFHRGAKWTTAPKPLMSDELFDYSYPVDNKEEQHRLGLQQKY<br>VTTEFEPCFDAADFLRAGKDIFVQKSQVTNRFGIEWMRRHLGDQY<br>NVHILSFNDSNPMHIDASYTFLEPGLLMENPERRCHQTNSIFVKAG<br>WKVVDAPRPAIPDSHPLWMTSKWLSMNTLMLGPKRVVVEAGEIP<br>TQKAFEKLGIECIKVPLRFAQSLGGGFHCWTCDVRRRGTLESYL
OroD	MFAMACRPFTQRLLREVAKHHLQLSKRAASALPNPIDVRSDVSYP<br>LELAFVTENYHALHEESLKNPERFWGDLGRRRLRWMKNFDEVMD<br>CDMRRGKISWFNGGVLNATENCLDRHVEEDPDRVALIWEKDEPN<br>QHENITYKELLEITCQLANALKRRGVKKGDRVIVYMPTSPYVVAS<br>MMACARIGAVHSVVYAGFSPEALRSRILDAEANTVITANEAVRDG<br>KIIPLKAMVDEAVEGCDCVKQVFVAQRTSANVPMYGRDIPMEEAL<br>ASESTTCPPEPMDSEDMLYILYTSGSTAKPKGIVHTHAGYLVFAST<br>TYQYTFDYHPGDIFCCAADIGWIAGHTCVVYGPLLNGGTSVCFEST<br>PIYPNPGRYWEVIERLRVNQFFTAPTAVRLLIKSGDEFVKKYDRSS<br>CRVLACGGEPLNENAYWWLHNVVGEGRCTVVDNCWQTETGAV<br>MLASRPAPDHTTPKPGFVMRSFFGVDPVIVNHQGKELVHPRASGL<br>YCVRRPWPGMARTIYGDHEKYLNTYFRPFPGYYFTGDGVYRDAD<br>GHLQITGRVDDVIKIKGRRIGTAELERALNSDPRVAESAVVGYPHE<br>MFGEGIYAFVILKDGVTESVEEMAQSLKQLVKKKIGSFAVPQLVM<br>VTTGLPKTRSGKIMRRILRQVAANKEDNLGDISTLAEPSVVEDIVK<br>KHKEMQSQ
OroE	**MLQTWRFCNCATRRYLTNGANTILRHCSRTSKSQTTPTIST**STR<br>ALSITAEQDPIAVETLRHGVSLREHIVSTIVAGGREEIDDPFYLVDV<br>GRLIDLYQNWVKVLPTVQPYYAIKANPDPALLKMLGALGAGFDC<br>ASKNEIKAVLDVGFPPDCVIYAQPCKQISHLQYAAHIGVRMMTFD<br>SELELYKVKAHLPTTRLLLRIRADDPNAVLGLGNKFGCTVAEGWD<br>LLQTAKNMGIEVAGVSFHIGSLSESPAAFEIALEMAAEVFEYSRKL<br>GYNFSLLDIGGGFPGTRGSEAIFERMAAAINNGVSRHFSAYPGVKV<br>VAEPGRYFATSTHTLVVNTHSKKMTVDGNPAEKQFTYYINDGVY

	GAFNGVLIEEMKPIPYTVQEKPLAPLYKTTIWGPTCDCTDKICETH<br>LPELETGDWLYFKDMGAYTESAASTFNGMTRPKSYYYIAEAHRER<br>LKWLNRRALLDRCNRNEVEMQ
OroE_N_truncated	STRALSITAEQDPIAVETLRHGVSLREHIVSTIVAGGREEIDDPFYLV<br>DVGRLIDLYQNWVKVLPTVQPYYAIKANPDPALLKMLGALGAGF<br>DCASKNEIKAVLDVGFPPDCVIYAQPCKQISHLQYAAHIGVRMMT<br>FDSELELYKVKAHLPTTRLLLRIRADDPNAVLGLGNKFGCTVAEG<br>WDLLQTAKNMGIEVAGVSFHIGSLSESPAAFEIALEMAAEVFEYSR<br>KLGYNFSLLDIGGGFPGTRGSEAIFERMAAAINNGVSRHFSAYPGV<br>KVVAEPGRYFATSTHTLVVNTHSKKMTVDGNPAEKQFTYYINDG<br>VYGAFNGVLIEEMKPIPYTVQEKPLAPLYKTTIWGPTCDCTDKICE<br>THLPELETGDWLYFKDMGAYTESAASTFNGMTRPKSYYYIAEAH<br>RERLKWLNRRALLDRCNRNEVEMQ
OroF	**MQTTMKSVFLTVLIVSGFAGRVFAGGDG**STDYCLIGAGPGGMQ<br>VGYFLERAGRDYIIFEKGPTAGTFYTLYPRHRTLISINKRFTGKANK<br>EFNFRHDWNSLISDDPSLRMSVYTEKYFPDADTIVQYFQDYANNL<br>GLKIKYRTEIVDVSREEGNKNFQLKDQNGTVYSCRTLVVSTGIWV<br>PNIPKDFIGLEYAEGYESMSIDPKDYENKAVLVLGRGNAGFETANH<br>LASHAAFVHLTSRSRVKNAYQTHYVGDLRAINNNLLDGYQLKSL<br>DGLFESNLAPELKTSFQLEKDENGKIFMTHPESAKLAYVESLGLDK<br>PYDVVLRCMGFKFDFSIFNETARPEAHPTAGENKYPDIHPNYESVN<br>VPGMYFAGTNTHARDFKKSAGAFIHGFRYTARSLFHHLQWRYEGI<br>PWPSVTLPATDLLNYMAKRINEASGNYQMFGMLVDVALIRDNYT<br>VVYLQEVPLHGMYMFKEMTGQEIGSFIIWNFEYGPNYSGPSADVL<br>RENRYVGNPVIADQSNFLHPVVYYWKNPPKPDSTWISGSLPTPDH<br>YHHIVEDFFTDWSARRIHLIPLRRFLEAVLQADLRDFFAETCFELIL<br>MQGDNVPLACKQHYLQGIGISSSDKILNFGSPEAFMV
OroF_signal<br>peptide_truncated	STDYCLIGAGPGGMQVGYFLERAGRDYIIFEKGPTAGTFYTLYPRH<br>RTLISINKRFTGKANKEFNFRHDWNSLISDDPSLRMSVYTEKYFPD<br>ADTIVQYFQDYANNLGLNIKYRTEIVDVSREEGNKNFQLKDQNGT<br>VYSCRTLVVSTGIWVPNIPKDFIGLEYAEGYESMSIDPKDYENKAV<br>LVLGRGNAGFETANHLASHAAFVHLTSRSRVKNAYQTHYVGDLR<br>AINNNLLDGYQLKSLDGLFESNLAPELKTSFQLEKDENGKIFMTHP<br>ESAKLAYVESLGLDKPYDVVLRCMGFKFDFSIFNETARPEAHPTA<br>GENKYPDIHPNYESVNVPGMYFAGTNTHARDFKKSAGAFIHGFRY<br>TARSLFHHLQWRYEGIPWPSVTLPATDLLNYTAKRINEASGNYQM<br>FGVLVDVALIRDNYTVVYLQEVPLHGMYMFKEMTGHEIGSFIIWN<br>FEYGPNYSGPSADVLRENRYVGNPVIADQSNFLHPVVYYWKNPPK<br>PDSTWISGSLPTPDHYHHIVEDFFTDWSARRIHLIPLRRFLEAVLQA<br>DLRDFFAETCFELILMQGDNVPLACKQHYLQGIGISSSDKILNFGSP<br>EAFMV
OroG	MAGRMCTLISPIRALRSSSSSQYRHLSIHSGLRSATGSSDWEERYIH<br>CSKVPTRHFQQSLPRVQFPTITATCQSYLDAVKPLLNKEEYERTEKI<br>VQEFAKKGGVGEALNQRLRVKELENKDTSYITRCAWPDMFLGWR<br>TTLVLDLNPCFAMRDDPRTNDQADRATNVVRSFVRFYNALMDGK

	LEPDVVHRRPHITDTTWFRNFIRFVPKSFSTPVAKMFGATPLEMNH<br>YHQFSTTRRPRKGMDEVLRFNDKRHIVVLCRGHLYMLDAMQSNG<br>LPTPPSVLHSQLLSILSDPRPPPTHPVSYLTTLPRDKWAVMRDELES<br>SGQNKEALEKIDSALFMLSLDDHHPQTLEDTAYTMLHNQGANRW<br>FDKPVTAVVTKGGVFGTNVEHSRWDGTTMSRIFHEVYEDTVKHP<br>YVPQGRLEPSAVGVEKLDFVLSDNVKSEIEVARREVEDKVRPFEY<br>RQLEYKKYGKKYLKTQQLSGDAIAQLAYQIAVYRCHKTCFSTFHL<br>LWMRPYMHGRLEALRPVTMASKACAEAFDPSHPADVAEMEEKL<br>REACHMHVNLIKRGLAGQGFDNHLLALKTLAIRGGTKLPIFEDPSY<br>GKFDKTLLLASTLSPPRVFQHITVVGLCPMFPPDGYGMNYFVKDD<br>RIVSEVLSYNTKKEFESQTFIDAIEKVFDDIYAVLNGKNFKL

[1] Sequence represented as bold in OroA, E, and F were predicted as unstructured or signal peptide.

Table S5. Top 50 co-occurring domains in the sponge bundles detected.

PROTEIN 1	PROTEIN 2	counts	probability[1]
FAD/NAD(P)-binding domain	Methyltransferase domain	121	0.231800766283525
Acyl-CoA N-acyltransferases (Nat)	Glutathione synthetase ATP-binding domain-like	116	0.222222222222222
Acyl-CoA N-acyltransferases (Nat)	Methyltransferase domain	116	0.222222222222222
Acyl-CoA N-acyltransferases (Nat)	FAD/NAD(P)-binding domain	110	0.210727969348659
Glutathione synthetase ATP-binding domain-like	Methyltransferase domain	106	0.203065134099617
Methyltransferase domain	Short-chain dehydrogenase/reductase (SDR) superfamily signature	103	0.197318007662835
Methyltransferase domain	PLP-dependent transferases	101	0.193486590038314
Acyl-CoA N-acyltransferases (Nat)	PLP-dependent transferases	99	0.189655172413793
Acyl-CoA N-acyltransferases (Nat)	Short-chain dehydrogenase/reductase (SDR) superfamily signature	97	0.185823754789272
Aspartate Aminotransferase, domain 1	Methyltransferase domain	97	0.185823754789272
FAD/NAD(P)-binding domain	PLP-dependent transferases	97	0.185823754789272
FAD/NAD(P)-binding domain	Glutathione synthetase ATP-binding domain-like	90	0.172413793103448
Aspartate Aminotransferase, domain 1	Glutathione synthetase ATP-binding domain-like	87	0.166666666666667
Acyl-CoA N-acyltransferases (Nat)	Aspartate Aminotransferase, domain 1	85	0.162835249042146
Aspartate Aminotransferase, domain 1	PLP-dependent transferases	85	0.162835249042146
PLP-dependent transferases	Short-chain dehydrogenase/reductase (SDR) superfamily signature	84	0.160919540229885
Glutathione synthetase ATP-binding domain-like	PLP-dependent transferases	80	0.153256704980843
Glutathione synthetase ATP-binding domain-like	Short-chain dehydrogenase/reductase (SDR) superfamily signature	79	0.151340996168582
FAD/NAD(P)-binding domain	Short-chain dehydrogenase/reductase (SDR) superfamily signature	76	0.145593869731801
Aspartate Aminotransferase, domain 1	FAD/NAD(P)-binding domain	73	0.139846743295019

Methyltransferase domain	Pyridine nucleotide-disulphide oxidoreductase	73	0.139846743295019
Fe(2+) 2-oxoglutarate dioxygenase domain profile.	Methyltransferase domain	69	0.132183908045977
Fe(2+) 2-oxoglutarate dioxygenase domain profile.	Short-chain dehydrogenase/reductase (SDR) superfamily signature	68	0.130268199233716
Acyl-CoA N-acyltransferases (Nat)	Fe(2+) 2-oxoglutarate dioxygenase domain profile.	66	0.126436781609195
Cytochrome P450	Methyltransferase domain	66	0.126436781609195
FMN-linked oxidoreductases	Glutathione synthetase ATP-binding domain-like	64	0.122605363984674
Acyl-CoA dehydrogenase NM domain-like	Glutathione synthetase ATP-binding domain-like	62	0.118773946360153
Aspartate Aminotransferase, domain 1	Short-chain dehydrogenase/reductase (SDR) superfamily signature	62	0.118773946360153
Cytochrome P450	FAD/NAD(P)-binding domain	62	0.118773946360153
Fe(2+) 2-oxoglutarate dioxygenase domain profile.	Glutathione synthetase ATP-binding domain-like	62	0.118773946360153
Glutathione synthetase ATP-binding domain-like	Glutathione Transferase (cytosolic)	61	0.116858237547893
Glutathione Transferase (cytosolic)	Methyltransferase domain	60	0.114942528735632
Acyl-CoA N-acyltransferases (Nat)	Peptidase S8/S53 domain	59	0.113026819923372
Aspartate Aminotransferase, domain 1	Glutathione Transferase (cytosolic)	56	0.10727969348659
FAD/NAD(P)-binding domain	Peptidase S8/S53 domain	56	0.10727969348659
Carbon-nitrogen hydrolase	Methyltransferase domain	55	0.10536398467433
Fe(2+) 2-oxoglutarate dioxygenase domain profile.	PLP-dependent transferases	55	0.10536398467433
Acyl-CoA N-acyltransferases (Nat)	Cytochrome P450	54	0.103448275862069
Acyltransferase	Glutathione synthetase ATP-binding domain-like	54	0.103448275862069
FAD/NAD(P)-binding domain	Fe(2+) 2-oxoglutarate dioxygenase domain profile.	53	0.101532567049808
Glyceraldehyde-3-phosphate dehydrogenase-like, C-terminal domain	Methyltransferase domain	52	0.0996168582375479
LONG-CHAIN-FATTY-ACID--COA LIGASE	Methyltransferase domain	52	0.0996168582375479

Acyl-CoA binding protein	Methyltransferase domain	51	0.0977011494252874
Aspartate Aminotransferase, domain 1	Fe(2+) 2-oxoglutarate dioxygenase domain profile.	50	0.0957854406130268
Glutathione synthetase ATP-binding domain-like	Peptidase S8/S53 domain	50	0.0957854406130268
Glyceraldehyde-3-phosphate dehydrogenase-like, C-terminal domain	Short-chain dehydrogenase/reductase (SDR) superfamily signature	50	0.0957854406130268
Peptidase S8/S53 domain	Short-chain dehydrogenase/reductase (SDR) superfamily signature	50	0.0957854406130268
Acetyl-CoA synthetase-like	Methyltransferase domain	49	0.0938697318007663
Carbon-nitrogen hydrolase	Glutathione synthetase ATP-binding domain-like	49	0.0938697318007663
Cytochrome P450	Glutathione synthetase ATP-binding domain-like	49	0.0938697318007663

[1] probability = counts / total_clusters

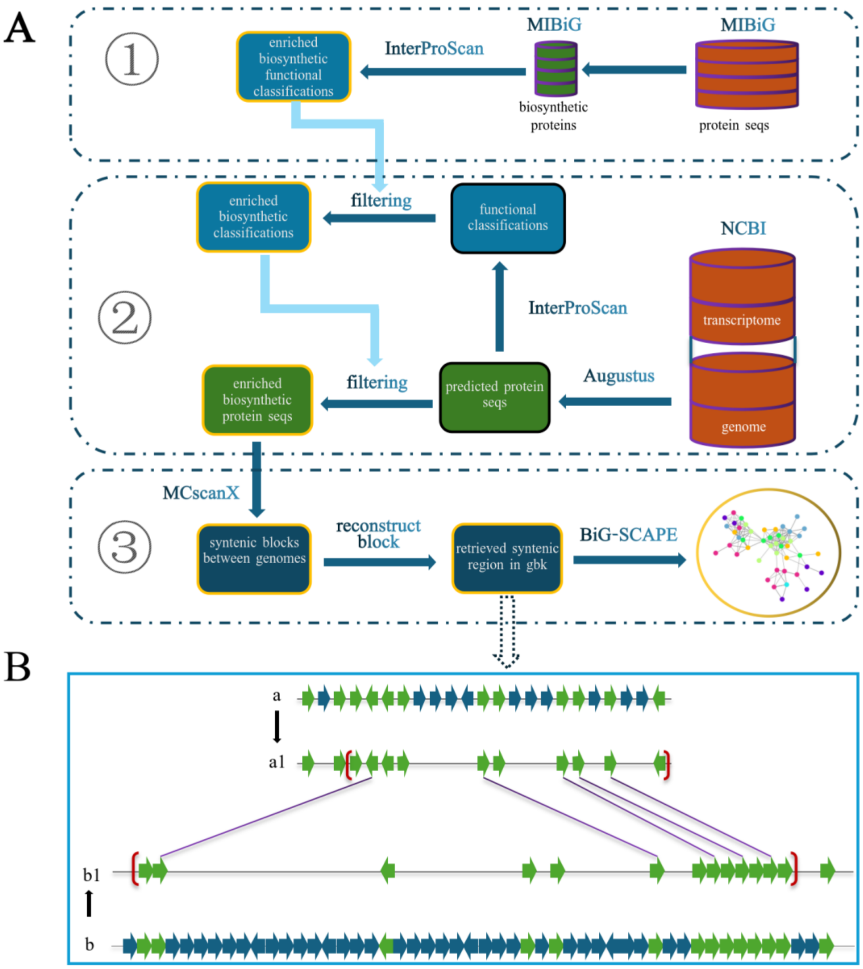

Figure S1. SynBGC strategy to identify noncanonical BGCs in animal genomes. A. Enrichment of biosynthetic functional classification. Biosynthetic protein sequences (n = 22,391) were extracted from the MIBiG database and re-annotated using InterProScan. Functional terms frequently associated with known biosynthetic enzymes were curated to generate a refined classification set, enabling targeted filtering of genome annotations to enrich biosynthesis relevant genes. **B.** An example is shown illustrating how non-biosynthetic genes were removed from two genomic regions (a and b), resulting in simplified annotations (a1 and b1). Syntenic

genes (linked by lines between a1 and b1) were then identified, and conserved blocks were extracted as candidate BGCs (indicated by brackets in a1 and b1).

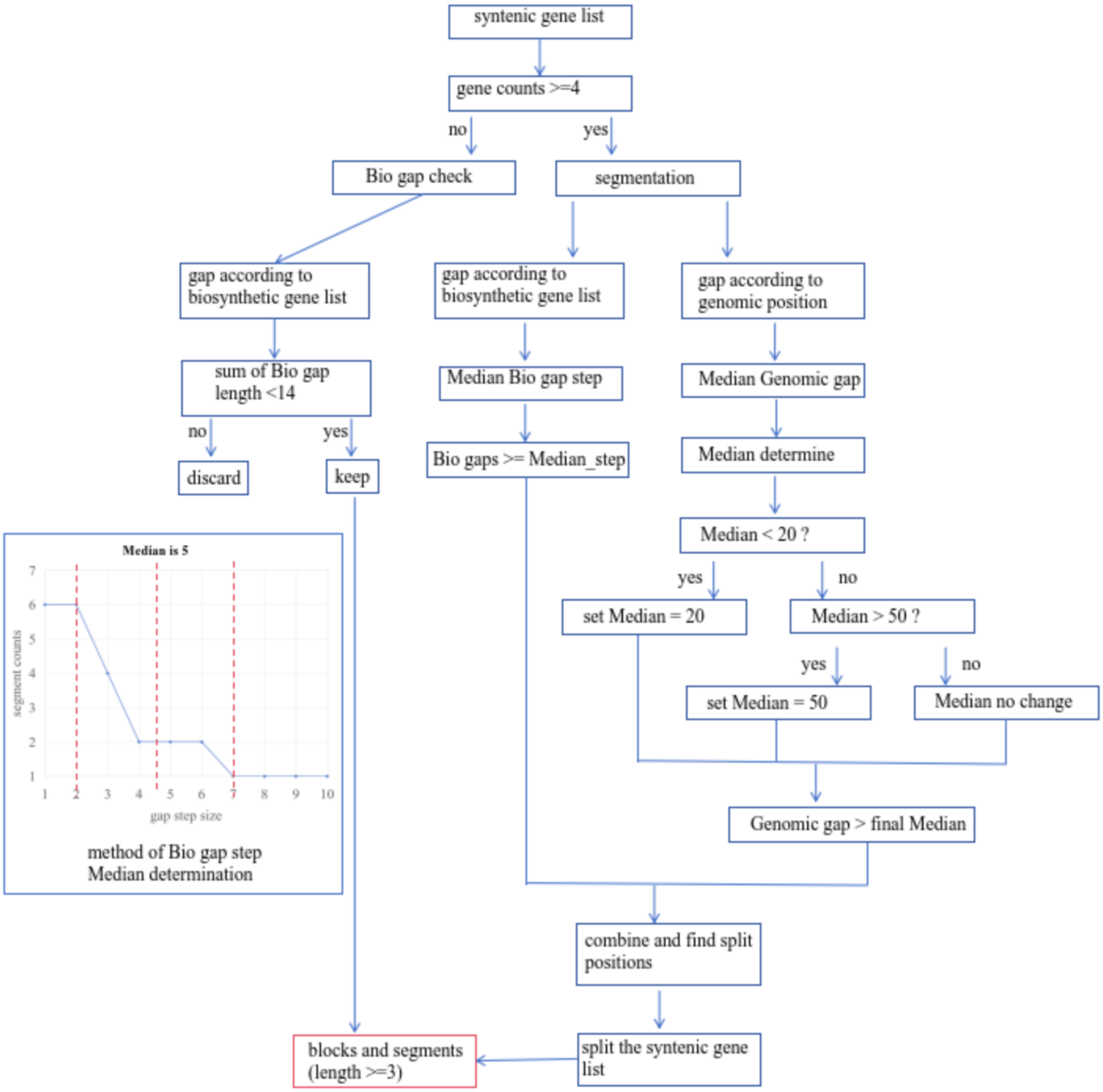

Figure S2. Diagram of segmentation of detected syntenic blocks by MCScanX. The box on the left describes how the threshold for splitting the gene list was determined.

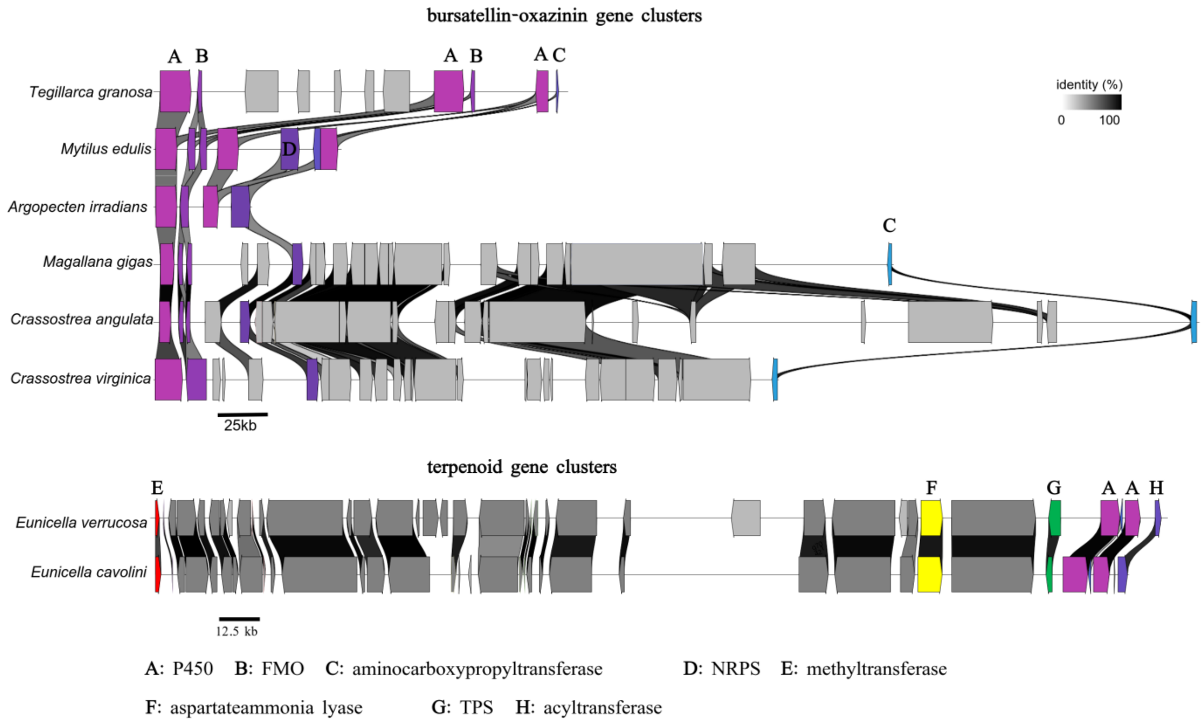

Figure S3. Gene organization of biosynthetic regions surrounding the bursatellin–oxazinin and terpenoid gene clusters detected by SynBGC. Open reading frames (ORFs) shown in gray represent non-synthetic genes that were excluded by the SynBGC pipeline. Gene cluster similarity was analyzed using clinker, highlighting conserved synteny and functional organization across species.

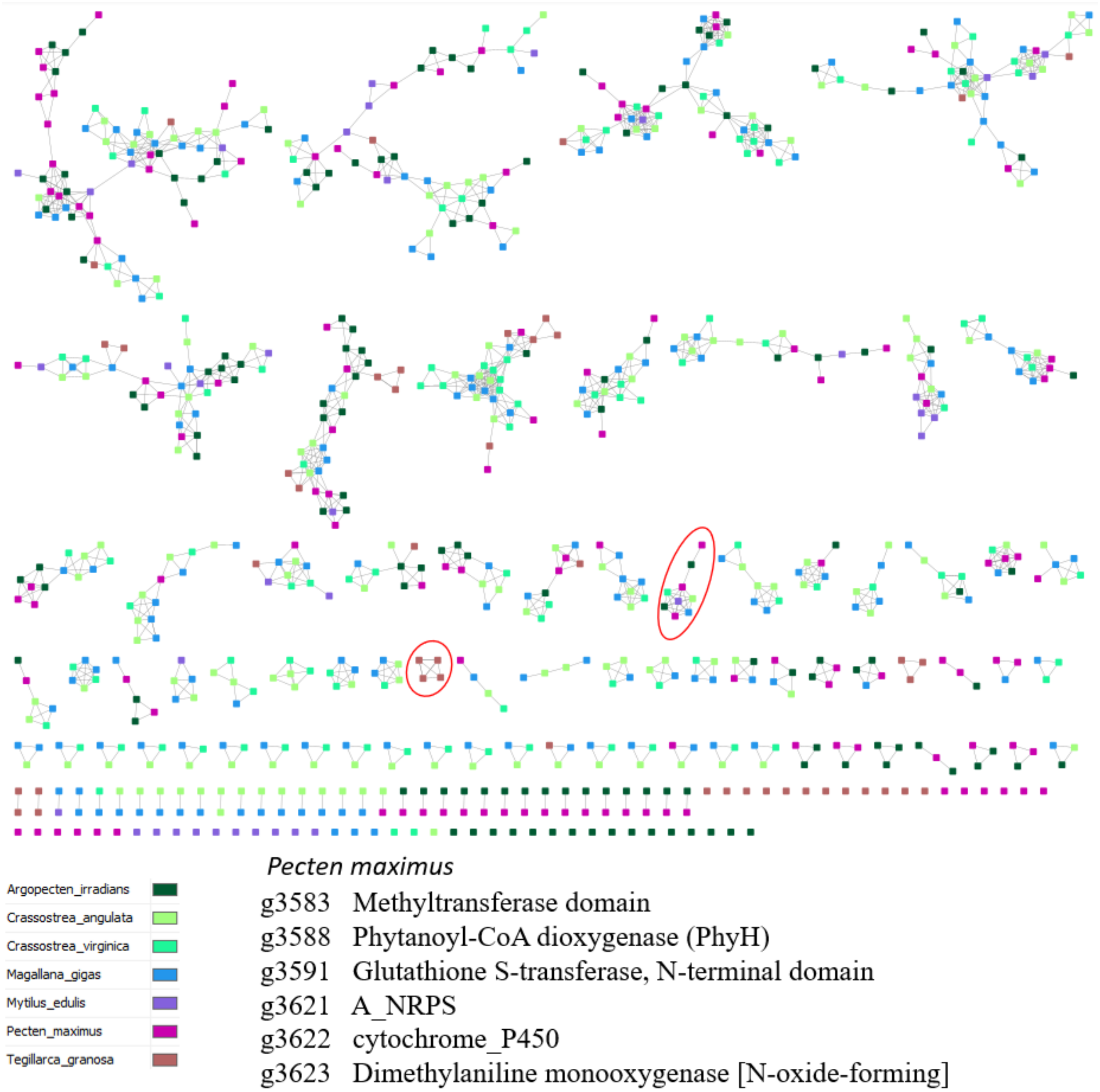

Figure S4. BGC similarity network of mollusc genomes. The GCF in red circle is for bursatellin-oxazinin BGCs. As an example, the bursatellin-oxazinin BGC from *Pecten maximus* is described at bottom.

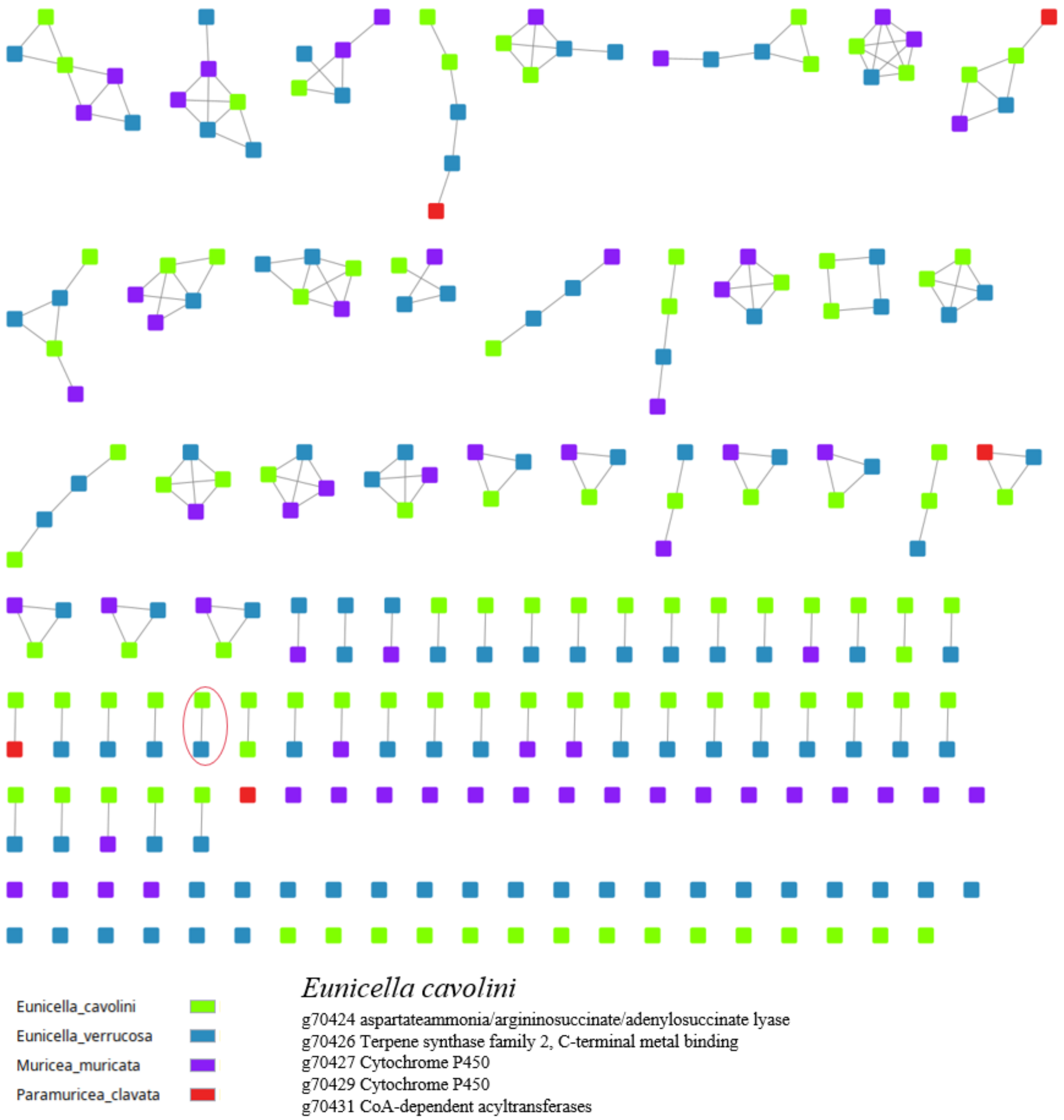

Figure S5. BGC similarity network of soft coral genomes. The GCF in red circle is for terpenoid BGCs, which only include terpenoid-producing species (*E. cavolini* and *E. verrucosa*), but not two species (*M. muricata* or *P. clavata*). An example of gene content in a terpene BGC is shown at bottom.

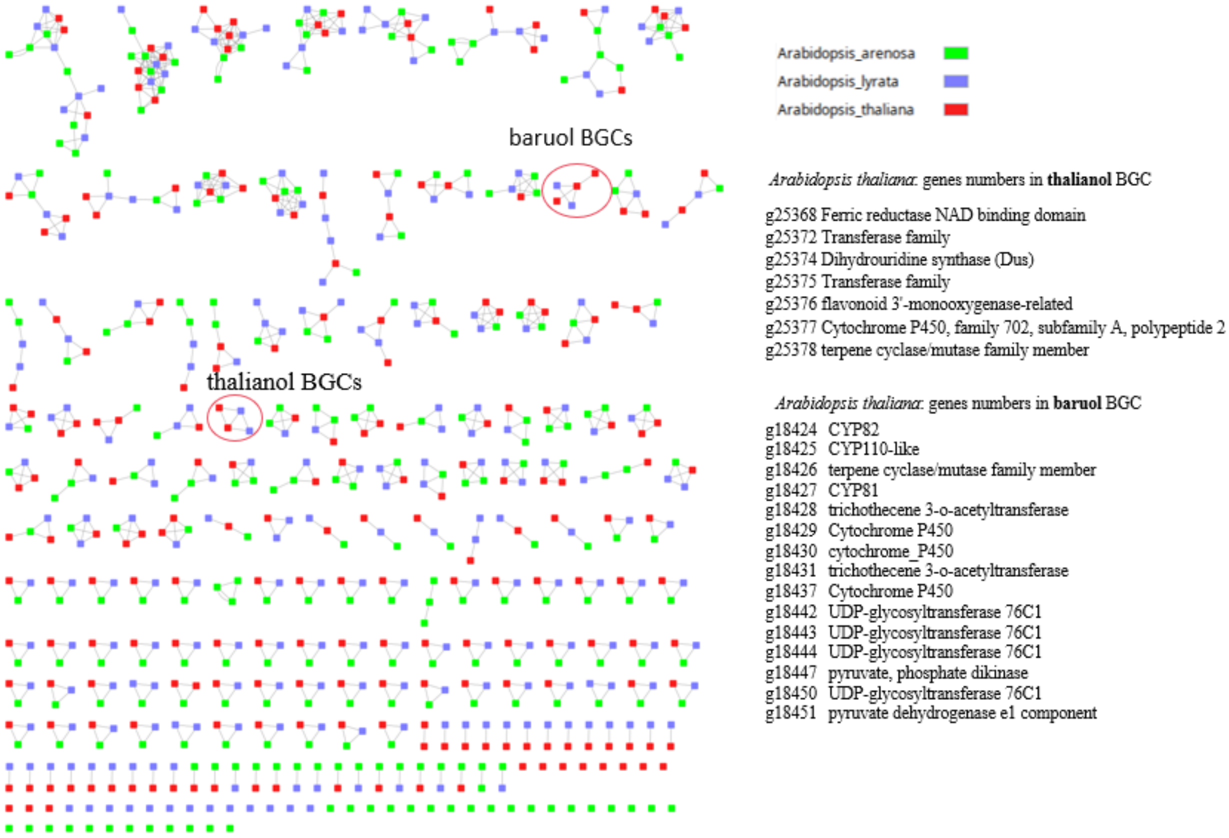

Figure S6. Identification of thalianol and baruol BGC using SynBGC.

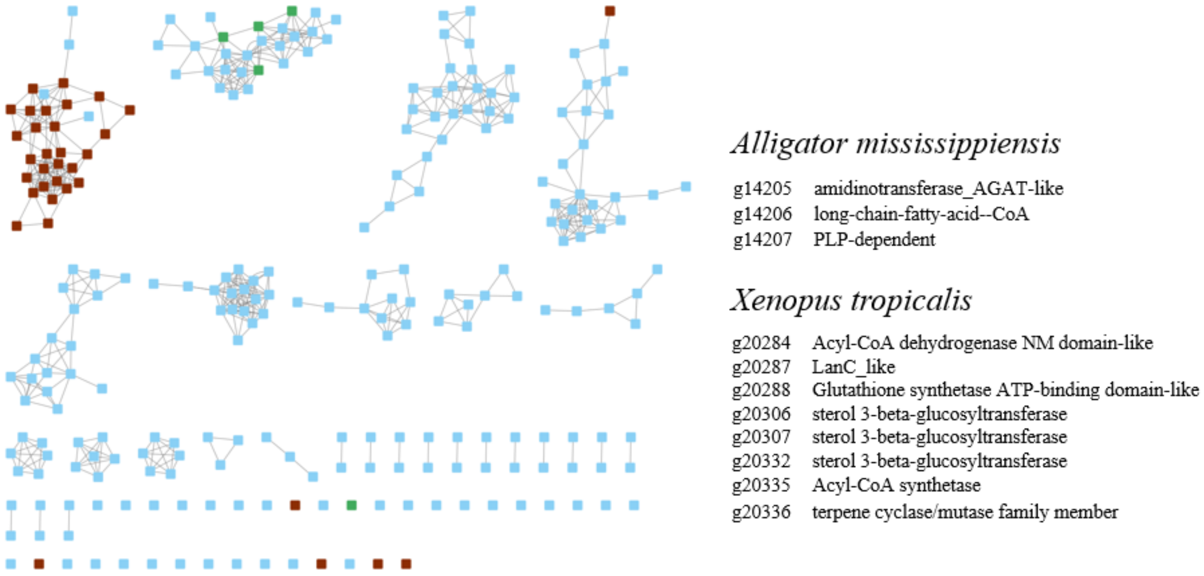

Figure S7. Conserved BGCs in vertebrate genomes (Table S1). As examples, potential creatine BGCs are marked in red. The BGCs containing terpene cyclase and glucosyltransferase are marked in green. Examples from individual animal species are shown at right.

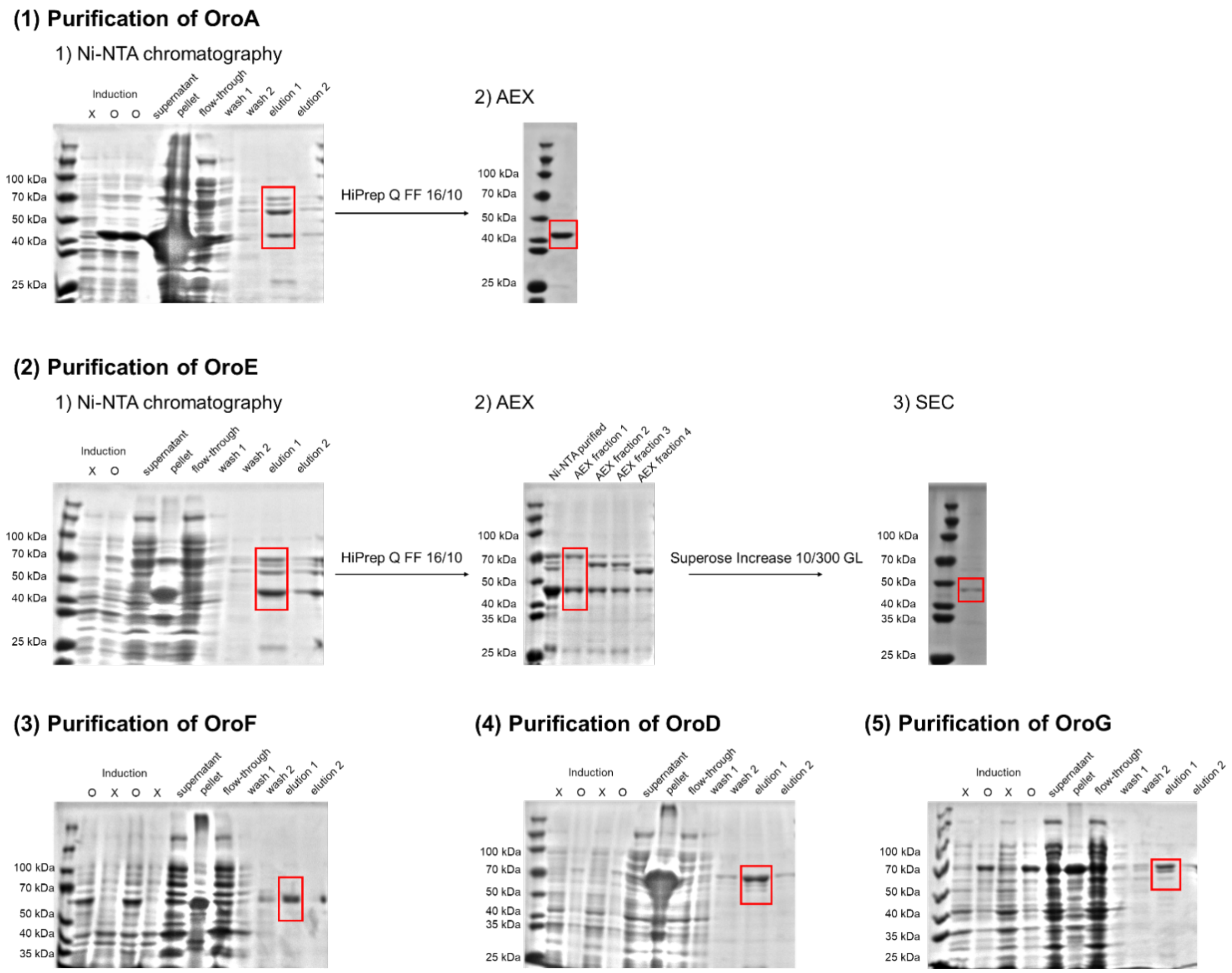

Figure S8. 10% Tris-glycine SDS-PAGE gel of purified enzymes. OroA and OroE were further purified after Ni-NTA purification due to co-purified *E. coli* chaperone proteins migrating around 70 kDa. The estimated molecular weights of the His_6-tagged proteins were calculated using Expasy ProtParam as 44,261.33 (OroA), 50,424.55 (OroE), 73,533.78 (OroD), 70,354.18 (OroF), and 79,447.75 (OroG) Da, respectively.

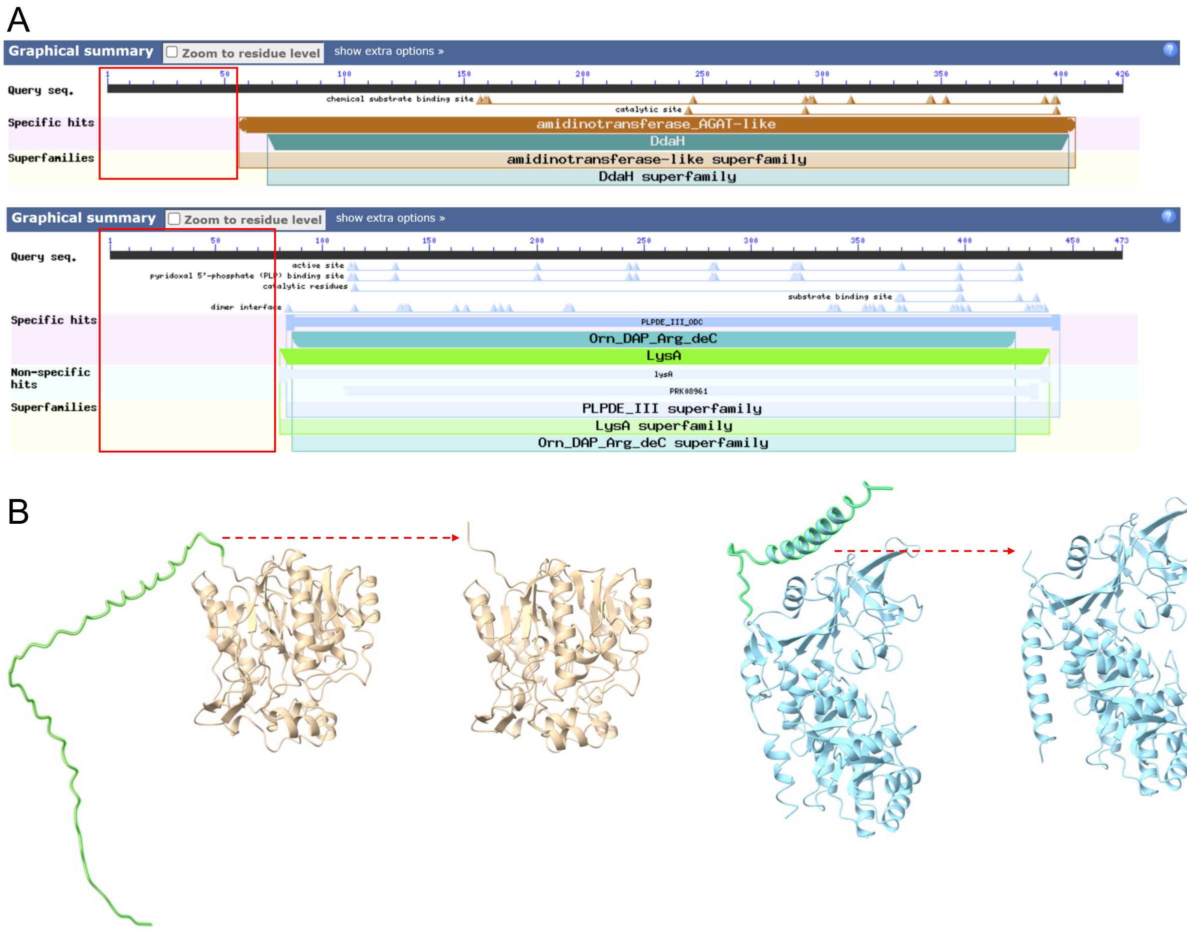

Figure S9. Conserved Domain Database (CDD) analysis and structure-guided truncation of OroA and OroE. A. CDD analysis revealed that the N-terminal regions of both OroA and OroE are located outside their respective conserved domains. **B.** AlphaFold 3 structural predictions indicated that these regions are intrinsically disordered and likely interfere with proper protein folding during heterologous expression (left: OroA, right: OroE). Guided by these predictions, the N-terminal segments were truncated, resulting in successful production of soluble protein (**Figure S8**).

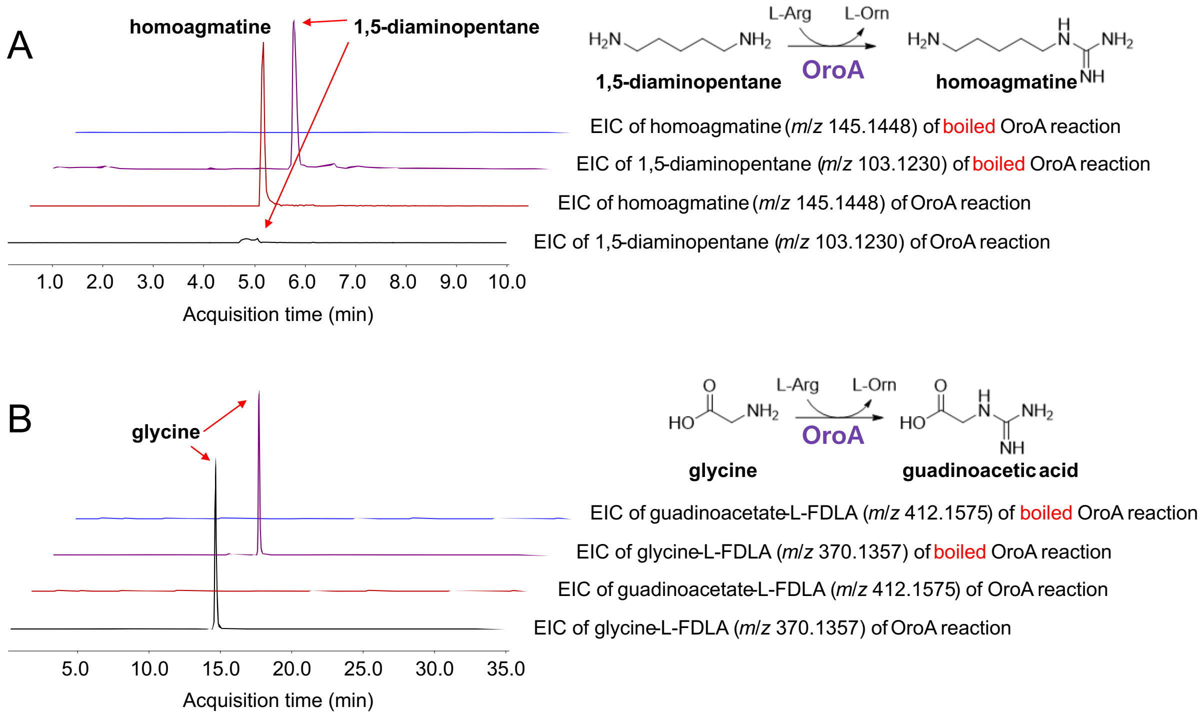

Figure S10. EICs from LC-MS analysis of the OroA reaction using other substrates. A. 1,5-diaminopentane (Method 1) and **B.** glycine (Method 3) as substrate with 24 hours incubation. Glycine was analyzed after derivatization with L-FDLA.

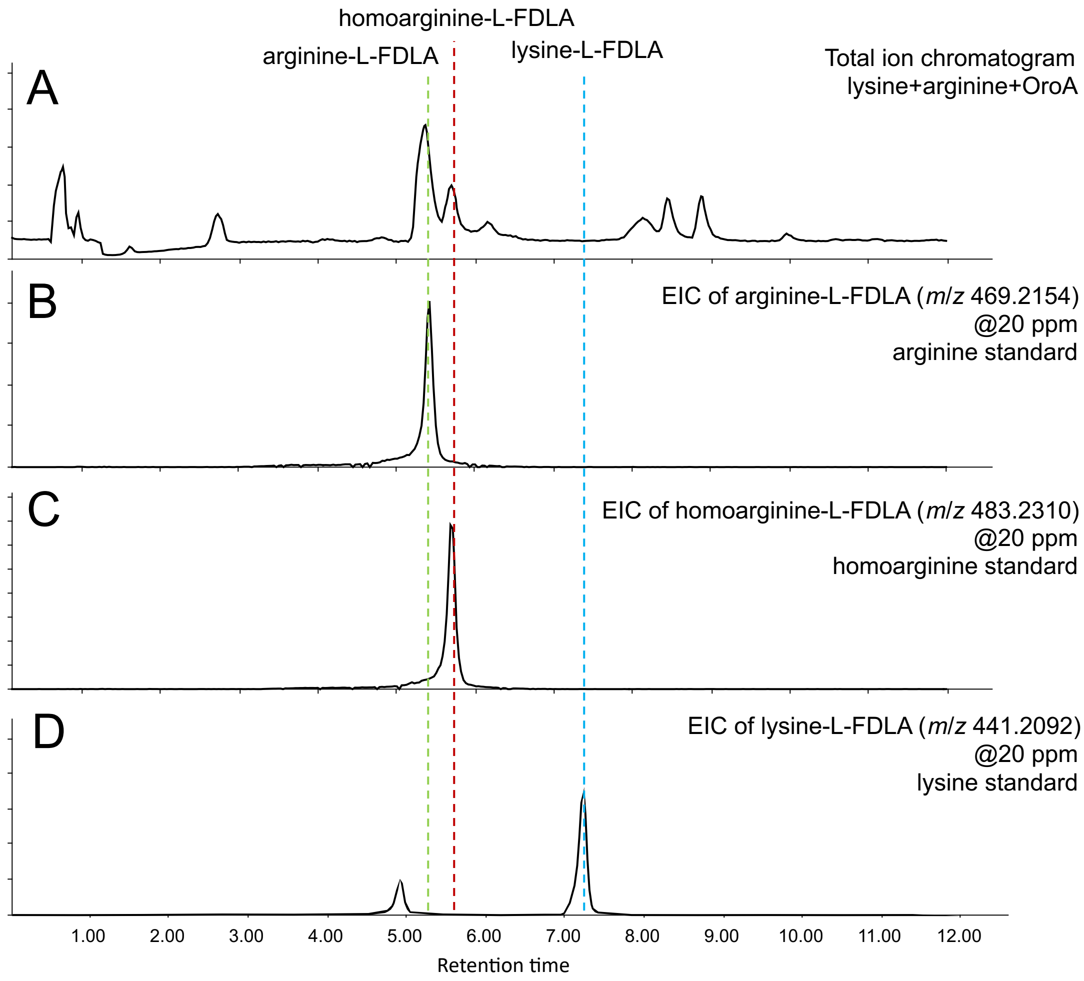

Figure S11. L-FDLA derivatization following the OroA reaction. A. Total ion chromatogram (TIC) of the reaction mixture (Method 3). **B.** Extracted chromatogram (EIC) of derivatized arginine-L-FDLA (*m/z* 469.2154). **C.** EIC of derivatized homoarginine-L-FDLA (*m/z* 483.2310). **D.** EIC of derivatized lysine-L-FDLA (*m/z* 441.2092).

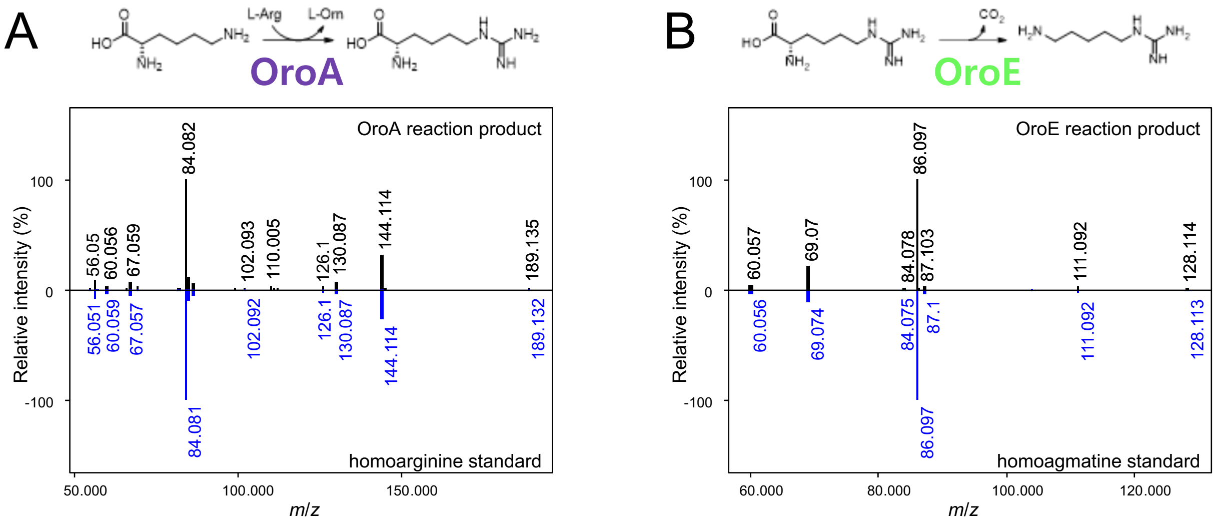

Figure S12. MS/MS mirror plot of OroA and OroE reaction product with authentic standard.

A. Mirror comparison of the MS/MS spectrum of homoarginine produced by OroA (black) with that of the standard (blue). **B**. Mirror comparison of the MS/MS spectrum of homoagmatine produced by OroE (black) with the standard (blue).

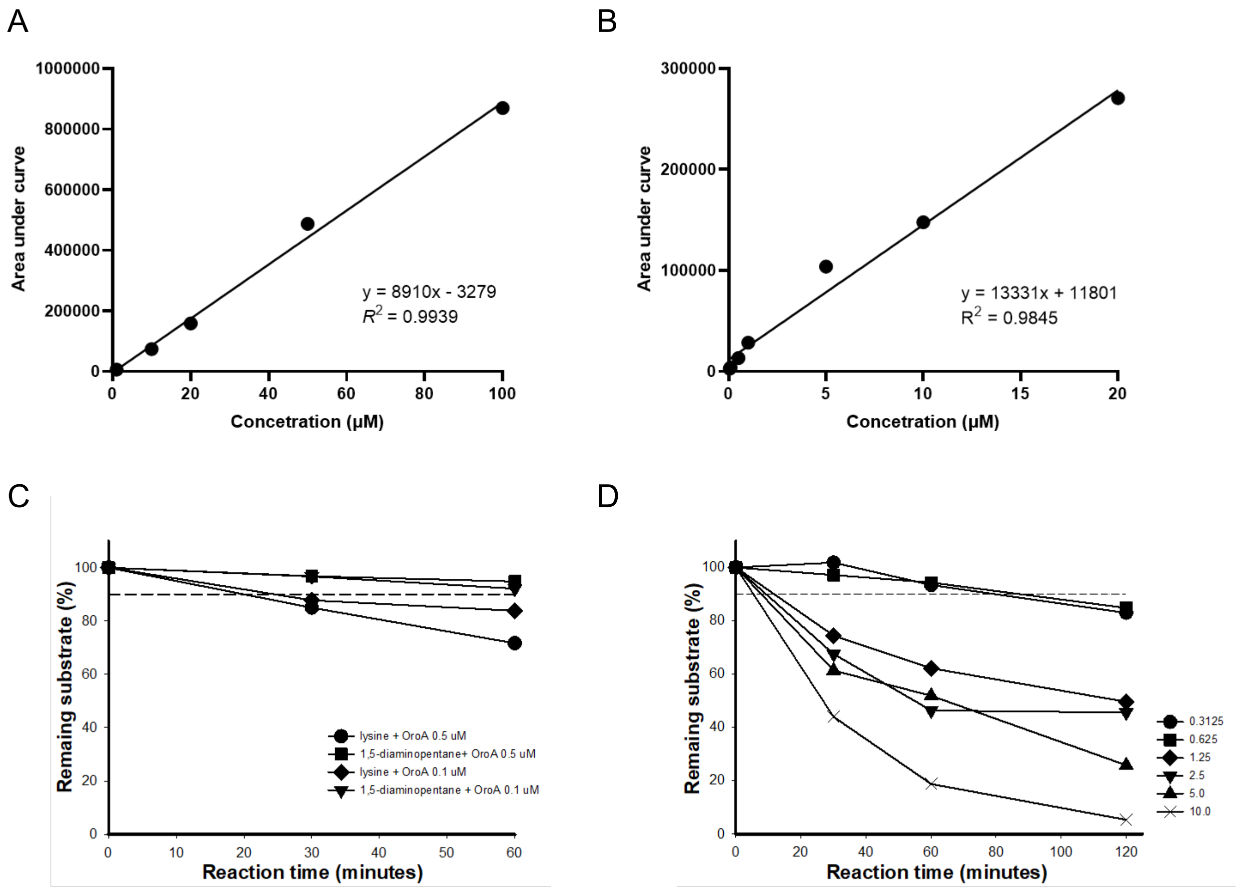

Figure S13. Determination of optimal enzyme concentration of OroA and OroE for kinetic analysis. A. Standard calibration curve for homoarginine. **B.** Standard calibration curve for homoagmatine. **C.** Time-course analysis of substrate consumption of OroA. **D.** Time-course analysis of substrate consumption of OroE with homoarginine. The area under the curve (AUC) for the remaining substrate at each time point was normalized to the AUC at 0.0 min and expressed as a percentage. The enzyme concentration that consumed less than 10% of the substrate over the assay period was selected for kinetic measurements.

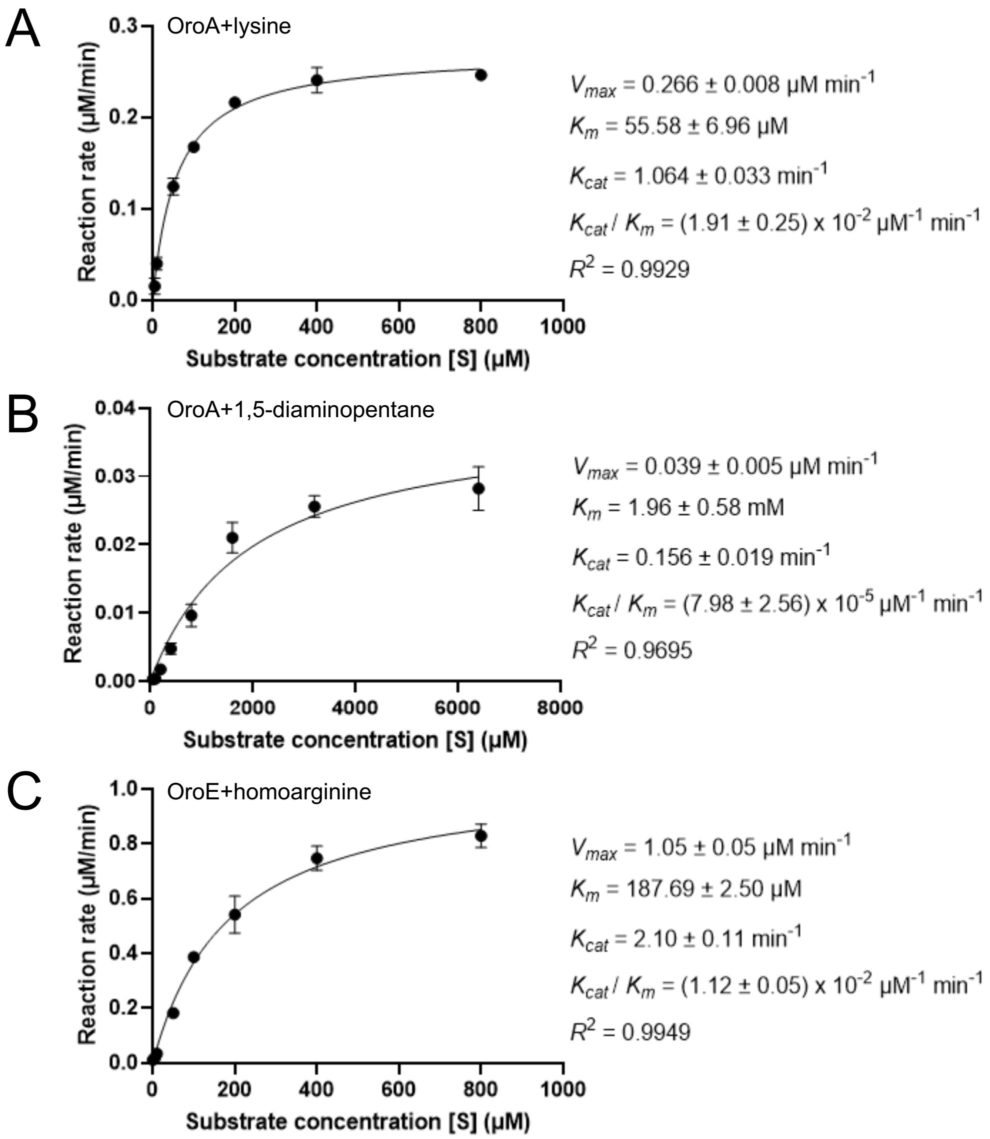

Figure S14. Kinetic analysis for Oro A and OroE reaction. A. Michaelis-Menten plot for OroA reaction with lysine. **B**. OroA reaction with 1,5-diaminopentane. **C.** OroE reaction with homoarginine.

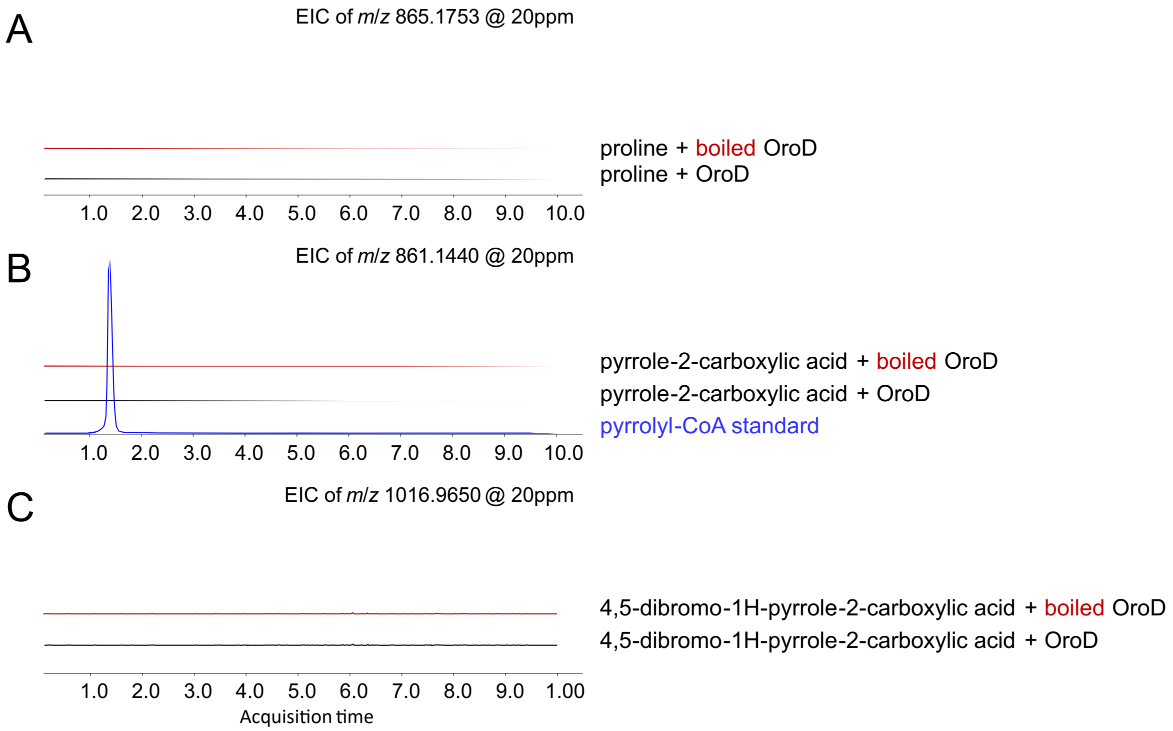

Figure S15. OroD reaction with possible substrates. A. EICs for prolyl-CoA (*m*/*z* = 865.1753) from proline+OroD reaction. **B.** EICs for pyrrolyl-CoA (*m*/*z* = 861.1440) from pyrrole-2-carboxylic acid+OroD reaction and synthetic pyrrolyl-CoA standard. **C.** EICs for 4,5-dibromo-1H-pyrrolyl-CoA (*m*/*z* = 1016.9650) from 4,5-dibromo-1H-pyrrole-2-carboxylic acid+OroD reaction.

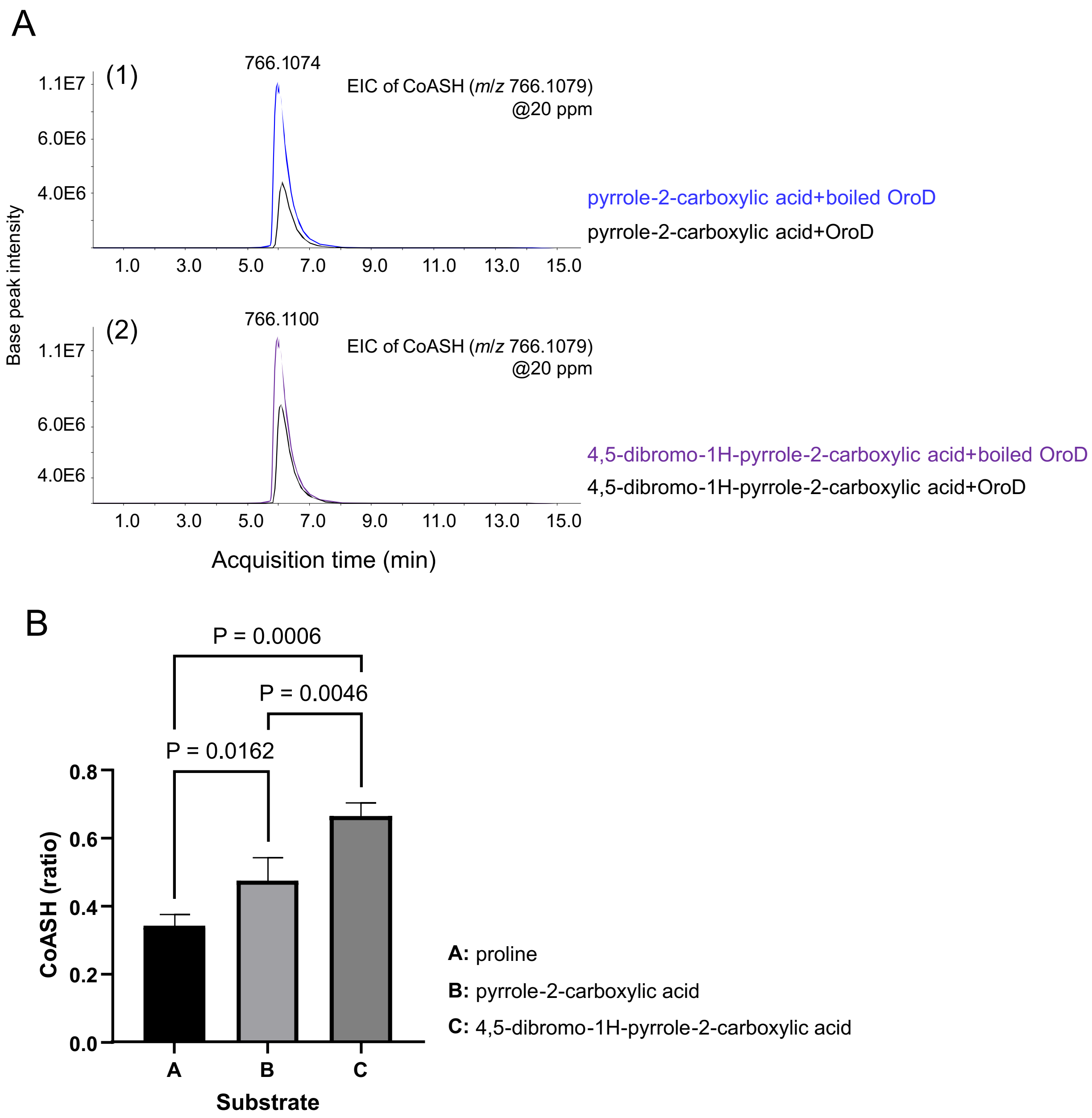

Figure S16. Coenzyme A consumption assay analyzed by LC-MS. A. EICs of remaining coenzyme A (CoASH, $[M-H]^-$, *m*/*z* = 766.1079) obtained using Method 2 in negative ionization mode. (1) pyrrole-2-carboxylic acid + OroD. (2) 4,5-dibromo-1-H-pyrrole-2-carboxylic acid + OroD. **B.** Comparison of AUC ratios of remaining CoASH between the active reaction and negative control (boiled OroD).

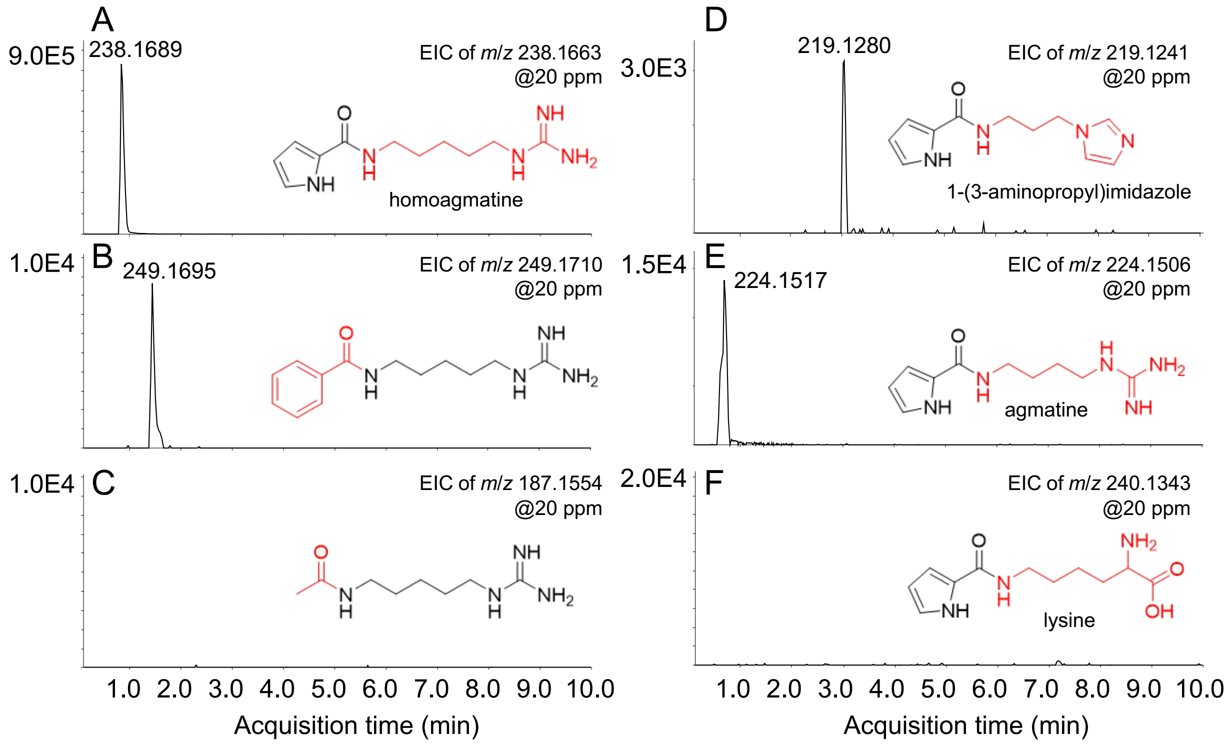

Figure S17. **EICs of acyltransferase assay with various substrate combinations. A.** pyrrolyl-CoA + homoagmatine (*m/z* 238.1663). **B.** benzoyl-CoA + homoagmatine (*m/z* 249.1710). **C.** acetyl-CoA + homoagmatine (*m/z* 187.1554). **D.** pyrrolyl-CoA + 1-(3-aminopropyl)imidazole (*m/z* 219.1241). **E.** pyrrolyl-CoA + agmatine (*m/z* 224.1506). **F.** pyrrolyl-CoA + lysine (*m/z* 240.1343). The product in panel **D** was analyzed using Method 1, while all others were analyzed using Method 2.

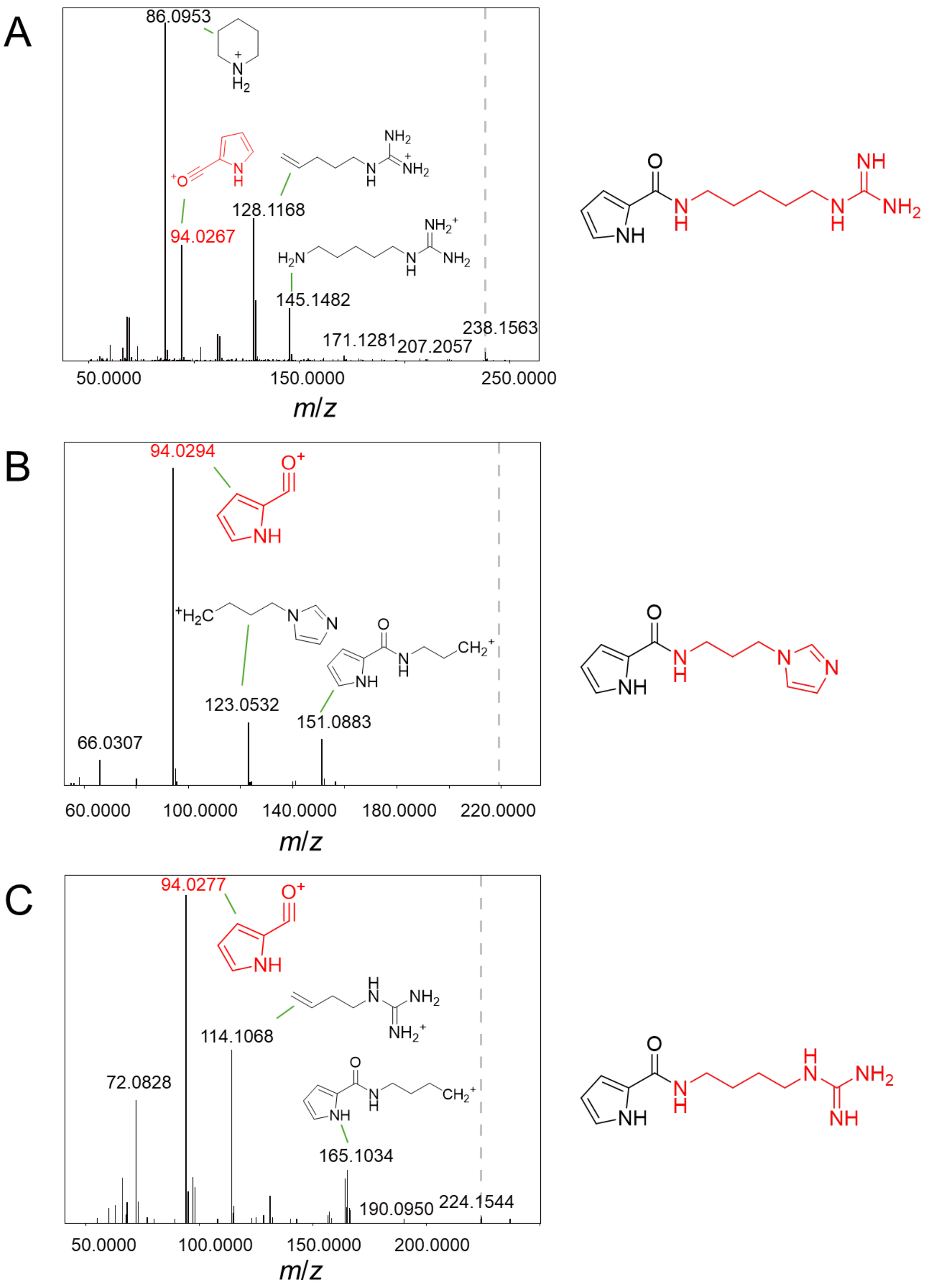

Figure S18. **MS/MS spectra of OroG-catalyzed ligated compounds. A.** pyrrolyl-CoA + homoagmatine (*m*/*z* 238.1663). **B.** pyrrolyl-CoA + 1-(3-aminopropyl)imidazole (*m*/*z* 219.1241). **C.** pyrrolyl-CoA + agmatine (*m*/*z* 224.1506). The gray dash line in the spectra indicates precursor ion.

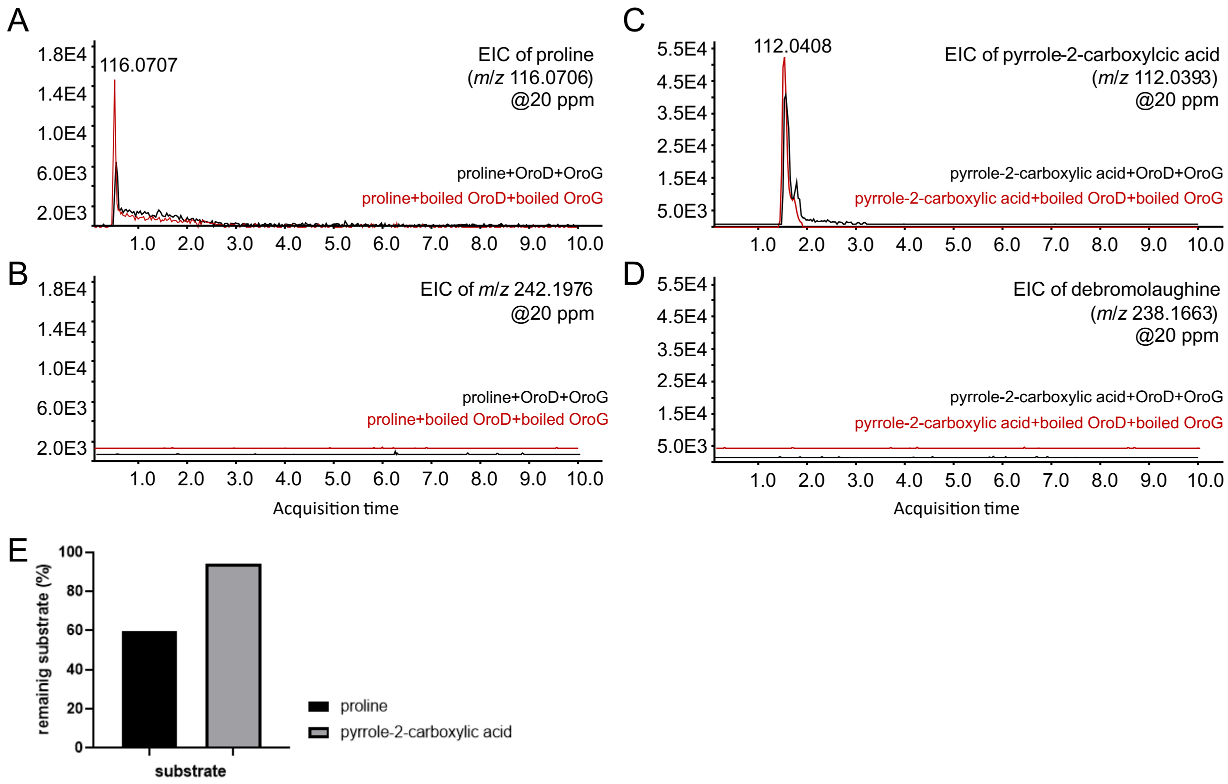

Figure S19. **OroD+OroG assay with proline and pyrrole-2-carboxylic acid. A.** EICs of residual proline (*m*/*z* 116.0706). **B.** EICs of expected ligation product derived from proline (*m*/*z* 242.1976). **C.** EICs of residual pyrrole-2-carboxylic acid (*m*/*z* 112.0390). **D.** EICs of expected ligation product, debromolaughine, derived from pyrrole-2-carboxylic acid (*m*/*z* 238.1663). The red traces in **A**-**D** represent the EIC of negative control (boiled enzyme). **E.** Comparison of substrate consumption between proline and pyrrole-2-carboxylic acid in combined OroD+OroG assay. The remaining substrate was quantified as %AUC relative to negative control.

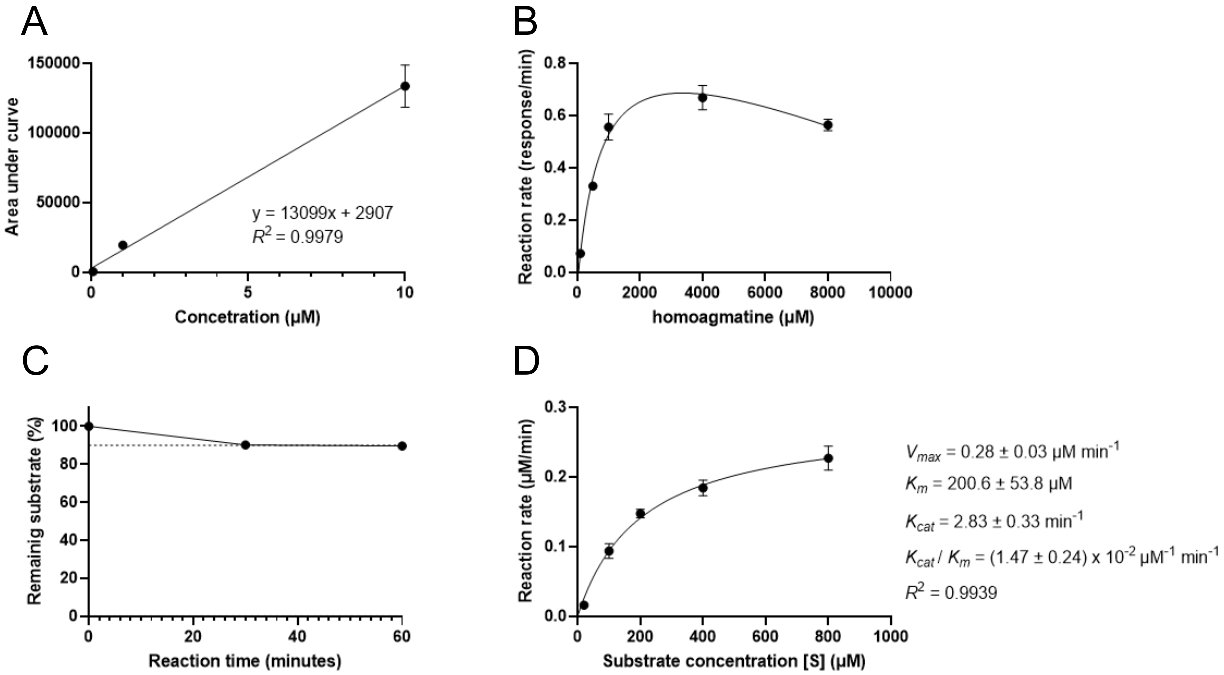

Figure S20. Kinetic analysis for OroG reaction. A. Standard calibration curve for debromolaughine. **B.** Reaction rate versus homoagmatine concentration; inhibition observed at 8 mM, with 4 mM used as saturation. **C.** Time-course analysis of substrate consumption by 0.1 μM OroG with 100 μM pyrrolyl-CoA and 4 mM homoagmatine. Less than 10% of substrate was consumed, consistent with the initial velocity condition. **D.** Michaelis-Menten plot for pyrrolyl-CoA under homoagmatine saturation.

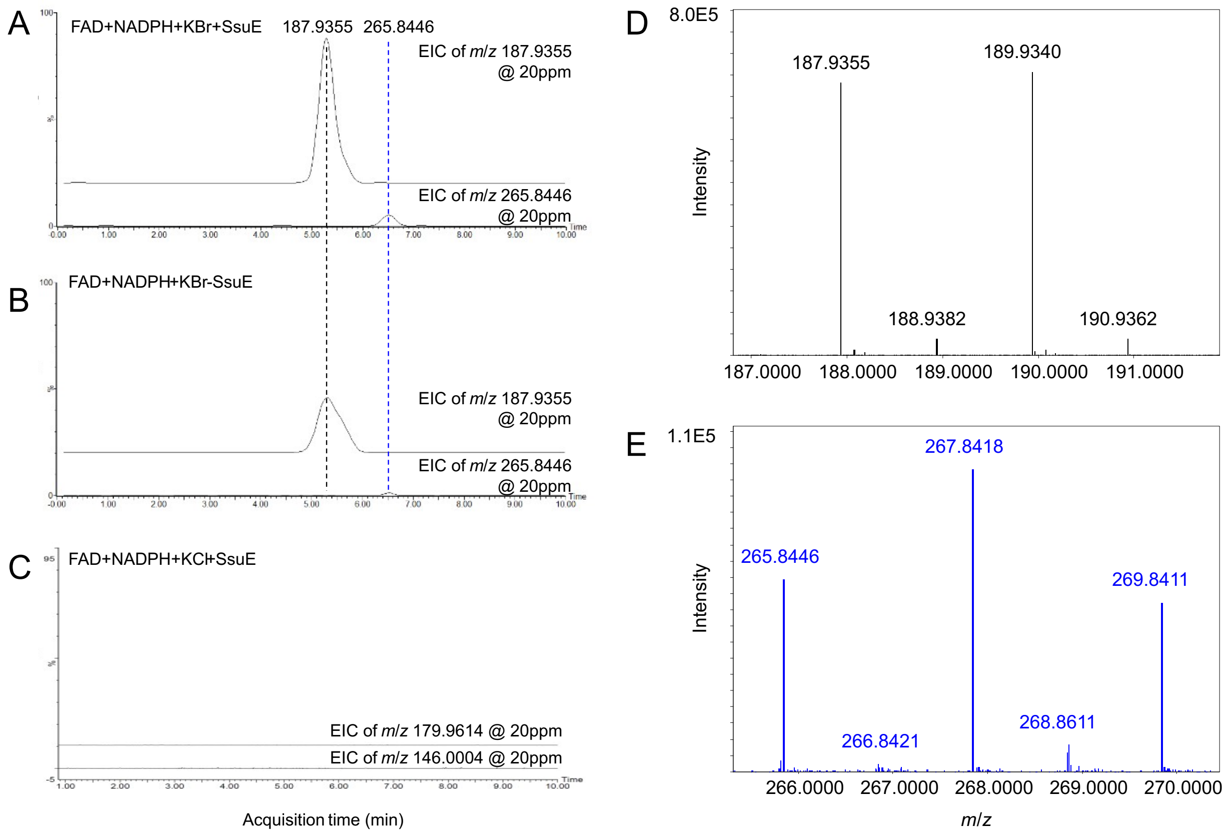

Figure S21. LC-MS analysis of in vitro assay for OroF. A. Reaction carried out with FAD and NADPH in the presence of flavin reductase SsuE. **B.** Reaction performed without SsuE. **C.** Reaction with KCl instead of KBr. EICs of mono- (*m/z* = 146.0004) and dichlorinated pyrrole-2-carboxylic acid (*m/z* 179.9614) indicate that OroF does not catalyze chlorination. **D.** MS spectrum of monobromopyrrole-2-carboxylic acid (*m*/*z* 187.9355) and **E.** dibromopyrrole-2-carboxylic acid (*m*/*z* 265.8446). Reactions were analyzed by Method 2.

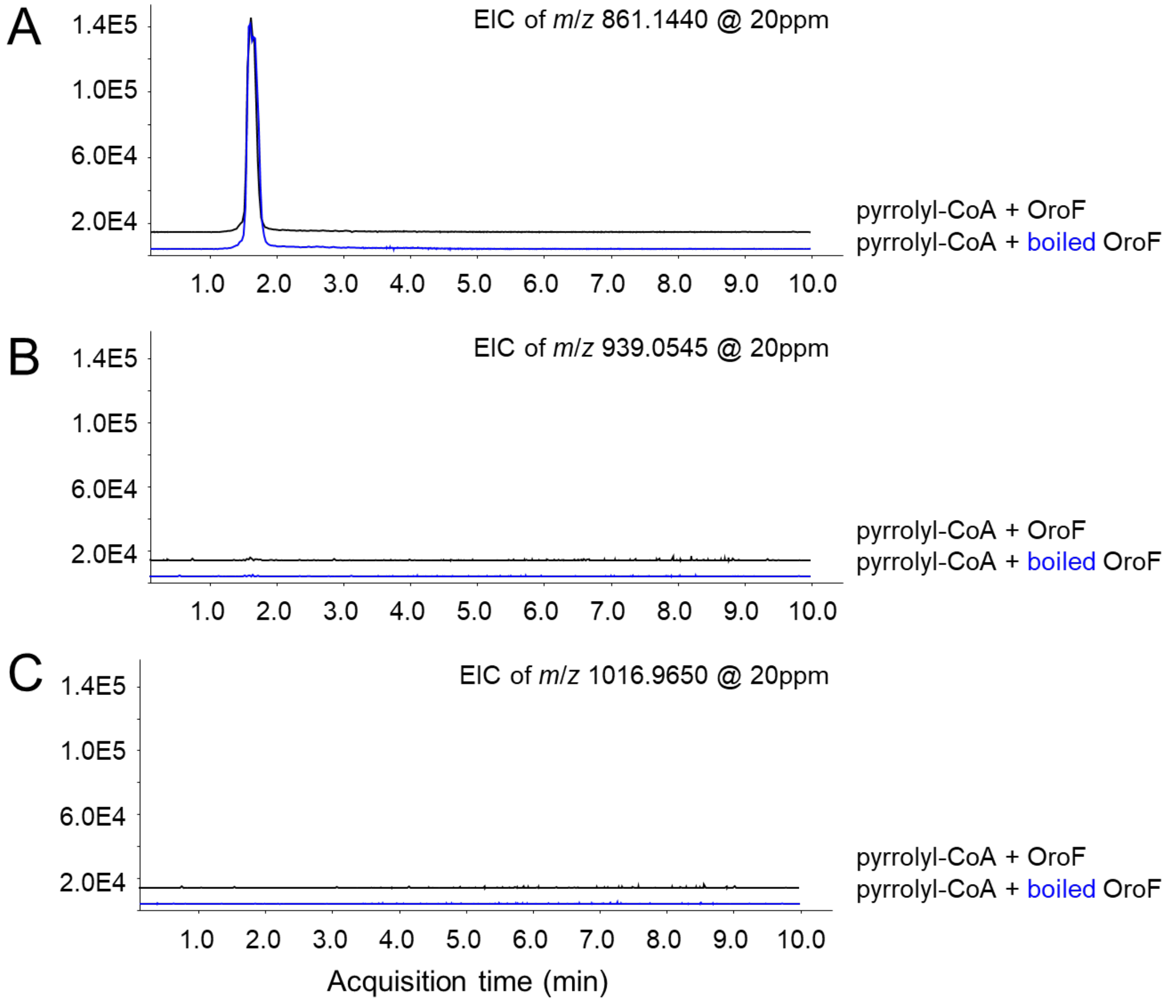

Figure S22. LC-MS analysis of OroF assay with pyrrolyl-CoA. A. EIC of pyrrolyl-CoA. Compared to negative control, a similar amount of substrate remained in both reactions. **B.** EIC of mono-brominated pyrrolyl-CoA (*m/z* = 939.0545) and **C.** EIC of di-brominated pyrrolyl-CoA (*m/z* = 1016.9650) also indicated no-conversion of pyrrolyl-CoA to brominated derivatives. All reactions were analyzed by Method 4.

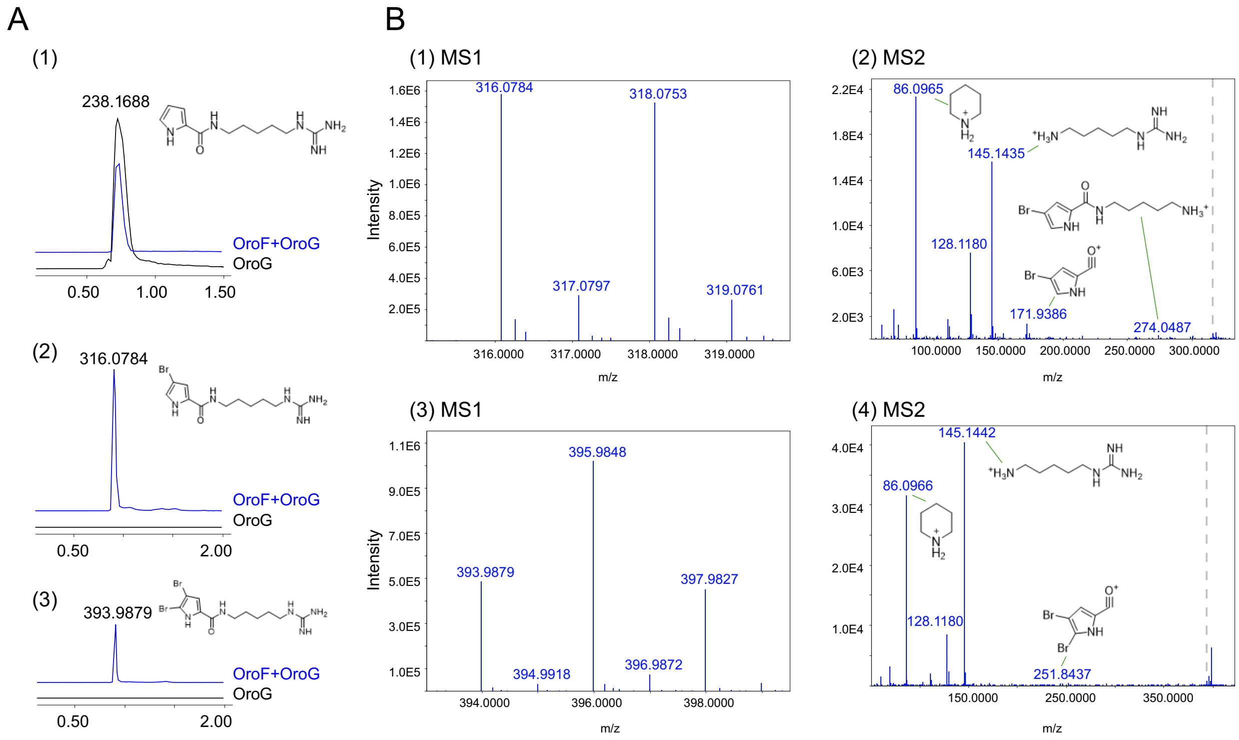

Figure S23. LC-MS analysis of one-pot reaction of OroF and OroG. **A.** EIC of (1) ligation product, (2) mono-brominated, (3) di-brominated ligation product in OroF+OroG assay. **B.** MS and MS/MS spectra of mono- and di-brominated ligation products. The mono- and dibrominated ligation products were analyzed by using Method 1, while non-brominated ligation product was analyzed using Method 2.

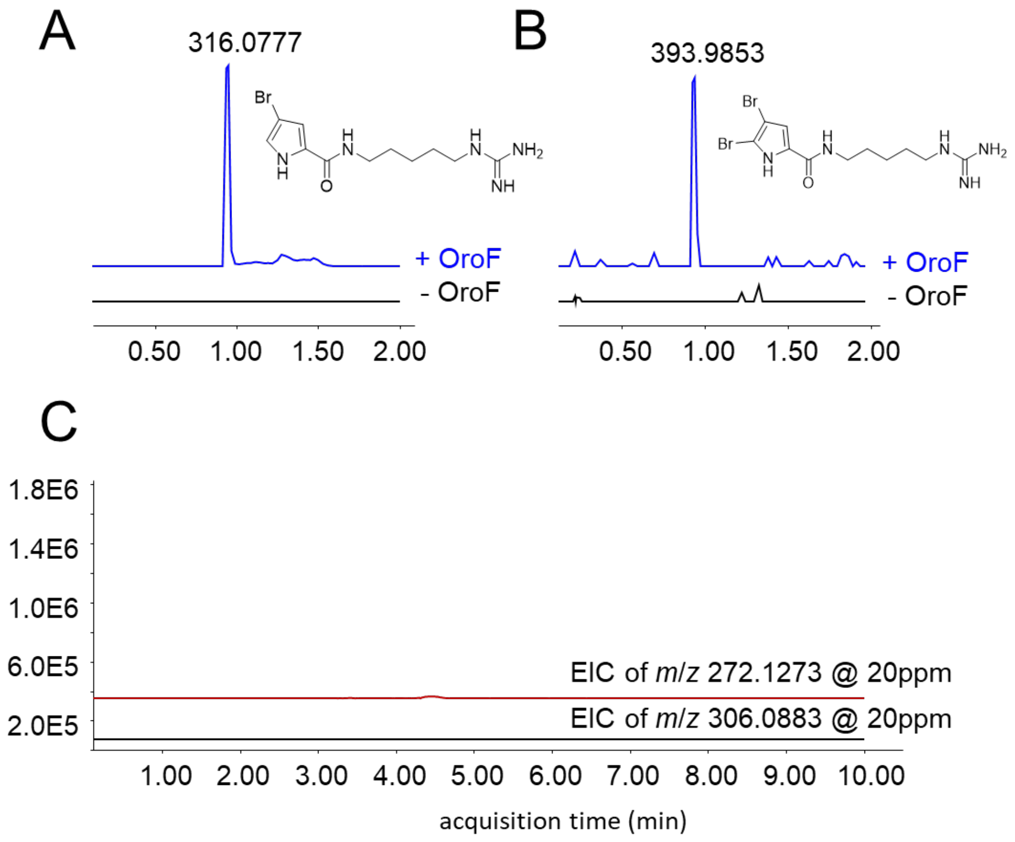

Figure S24. LC-MS analysis of OroF with synthetic debromolaughine. A. EIC of monobrominated product (*m/z* 316.0777; calc. 316.0768). **B.** EIC of dibrominated product (*m/z* 393.9853; calc. 393.9873). **C.** OroF assay performed with KCl, showing no detectable chlorinated products (calc. *m/z* = 272.1273 for mono- and 306.0883 for dichlorinated products).

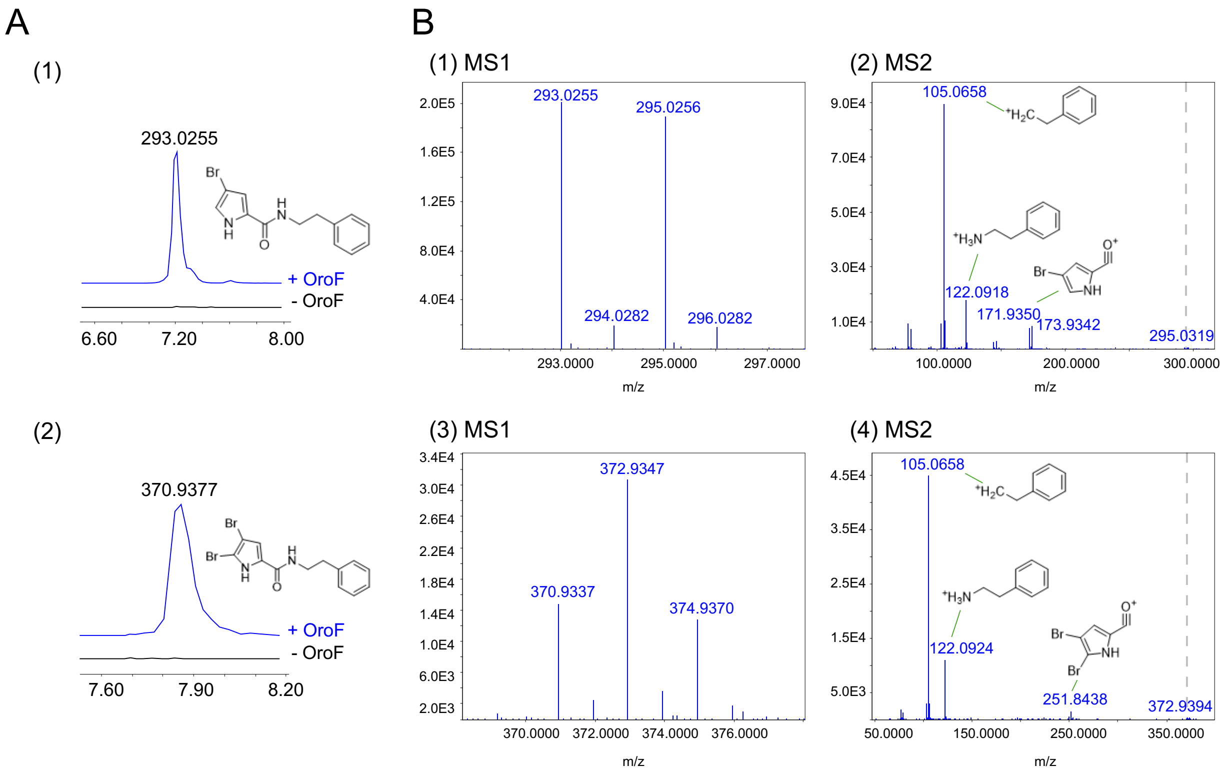

Figure S25. LC-MS analysis of one-pot reaction of OroF and OroG using *N*-(2-phenylethyl)-1*H*-pyrrole-2-carboxamide as substrate. A. EIC of (1) mono- and (2) dibrominated ligation products. Conversion of *N*-(2-phenylethyl)-1*H*-pyrrole-2-carboxamide was more efficient than that of pyrrole-2-carboxylic acid, further demonstrating that OroF catalyzes bromination after amide bond formation by OroG. **B.** MS and MS/MS spectra of brominated ligation products. All reactions were analyzed by Method 2.

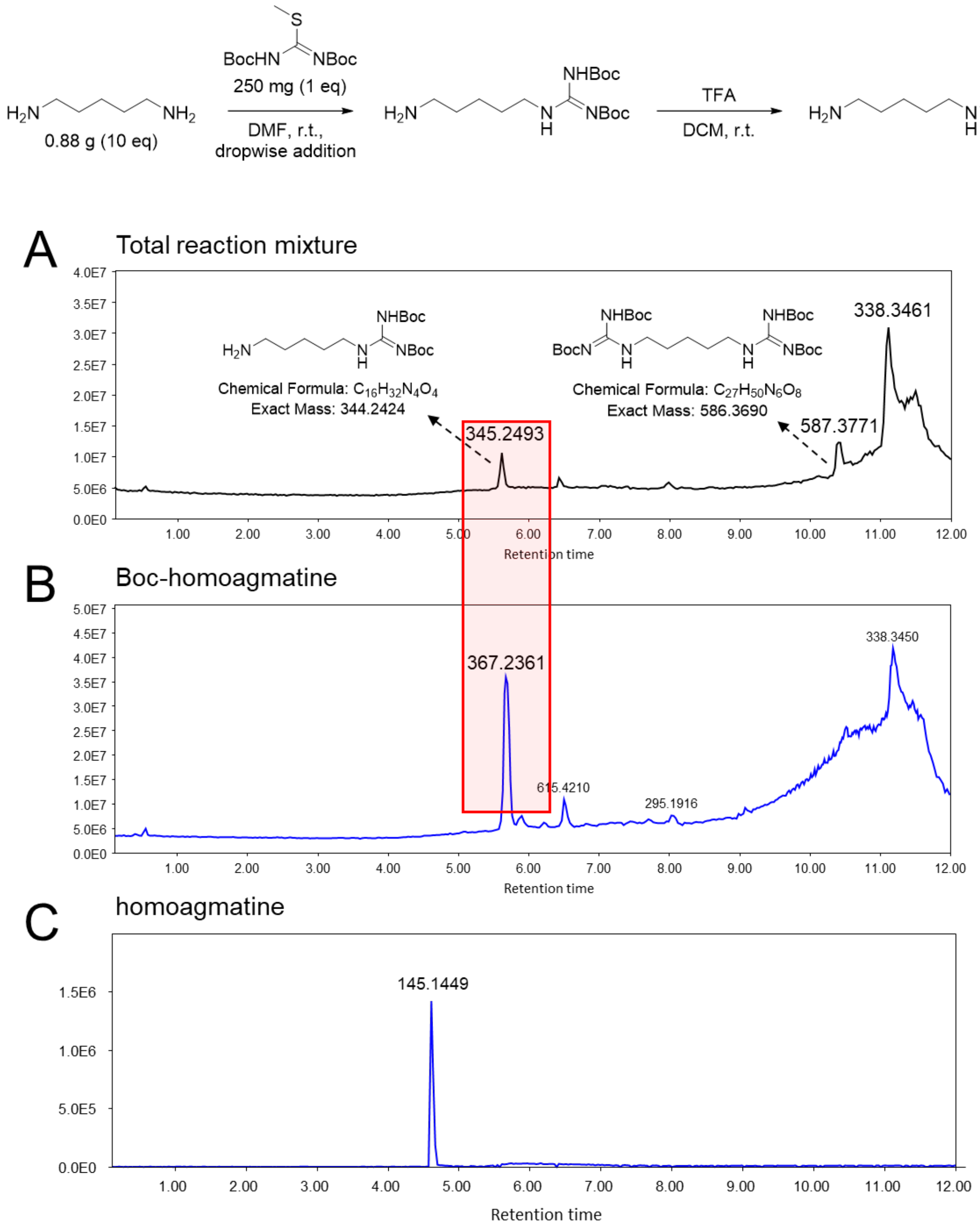

Figure S26. Synthesis of homoagmatine. TICs of the **A.** crude reaction mixture, **B.** Boc-protected intermediate, and **C.** purified homoagmatine. The crude mixture and Boc-protected intermediate were analyzed using Method 2, whereas homoagmatine was analyzed using Method 1.

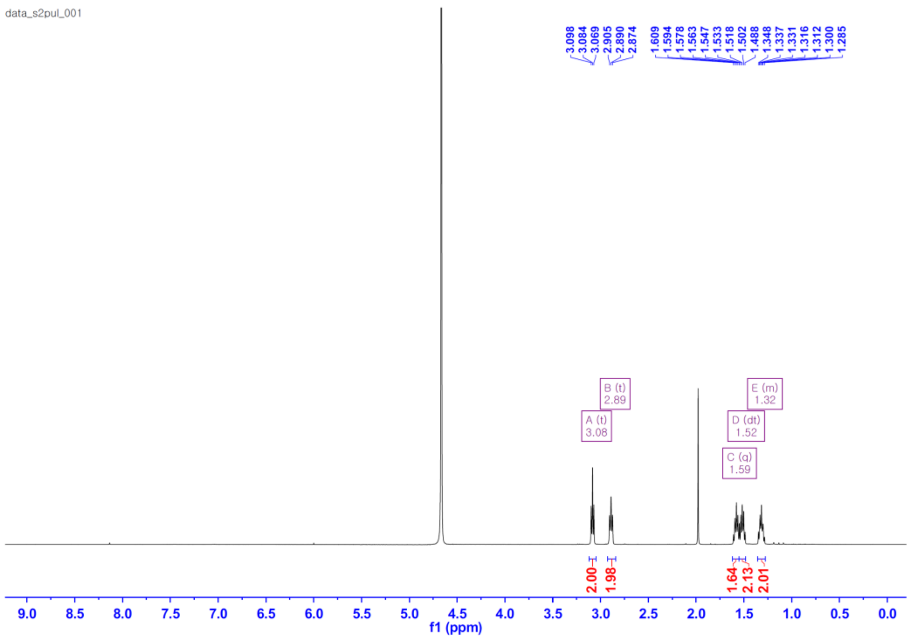

Figure S27. ^{1}H-NMR spectrum of homoagmatine (D_2O, 500 MHz).

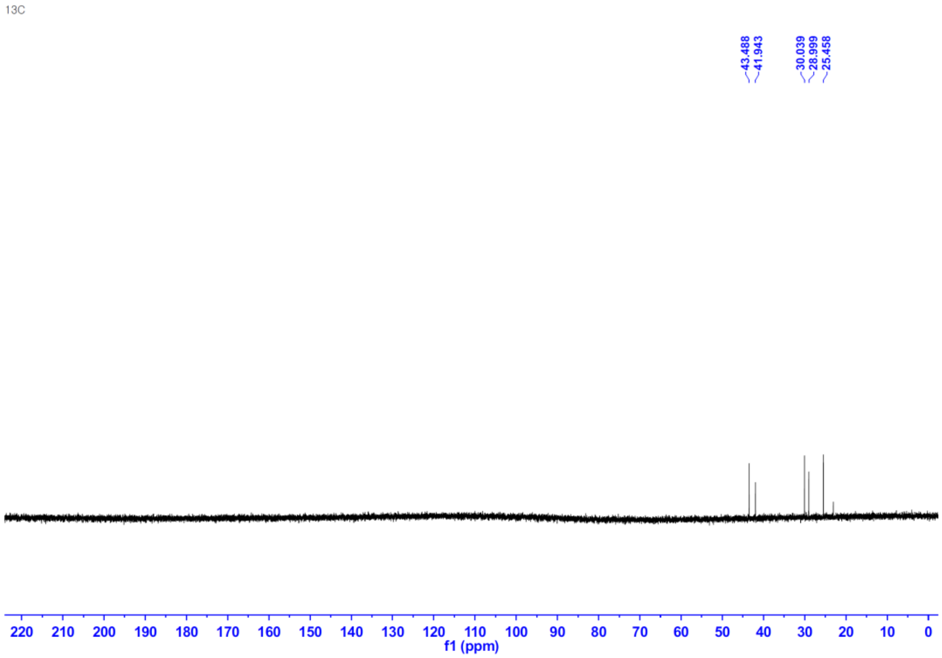

Figure S28. ^{13}C-NMR spectrum of homoagmatine (D_2O, 125 MHz). The peak at δ_C159.35 was not detected in ^{13}C-NMR but assigned by HMBC correlation.

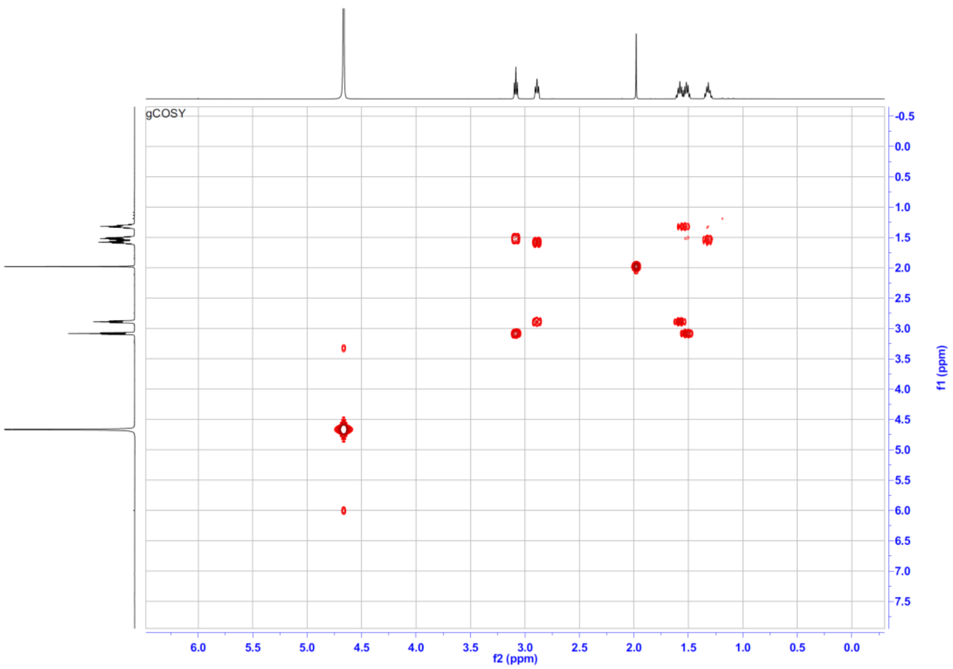

Figure S29. gCOSY spectrum of homoagmatine (D_2O).

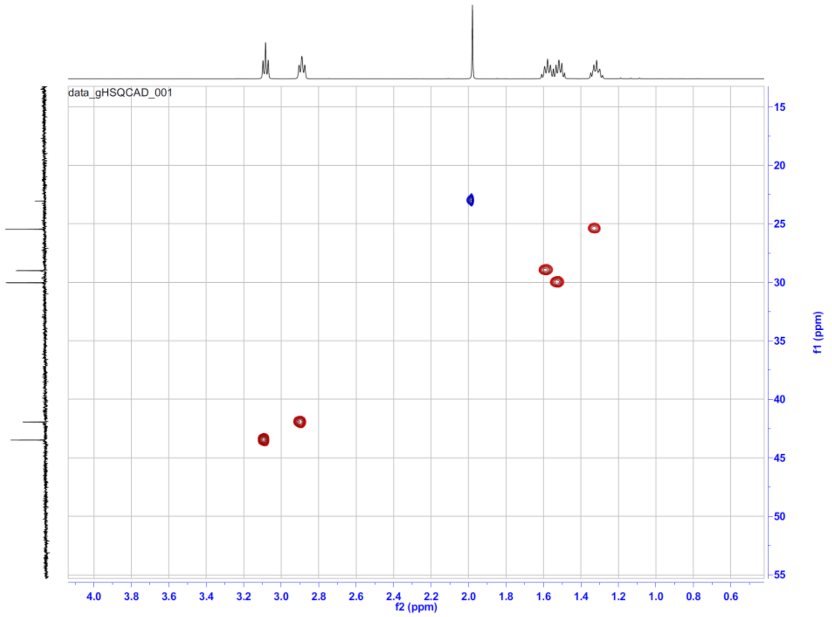

Figure S30. gHSQCAD spectrum of homoagmatine (D_2O).

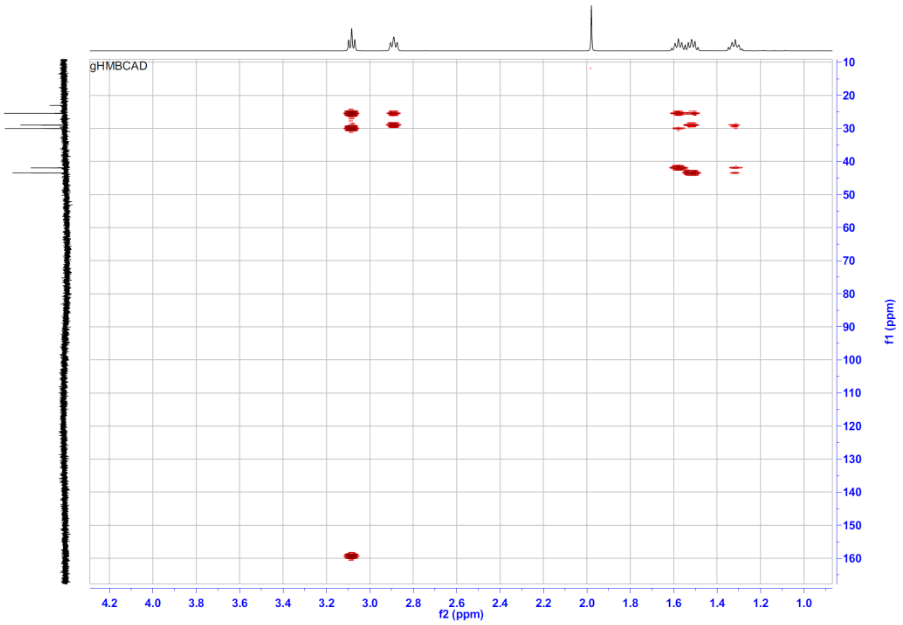

Figure S31. gHMBCAD spectrum of homoagmatine (D_2O).

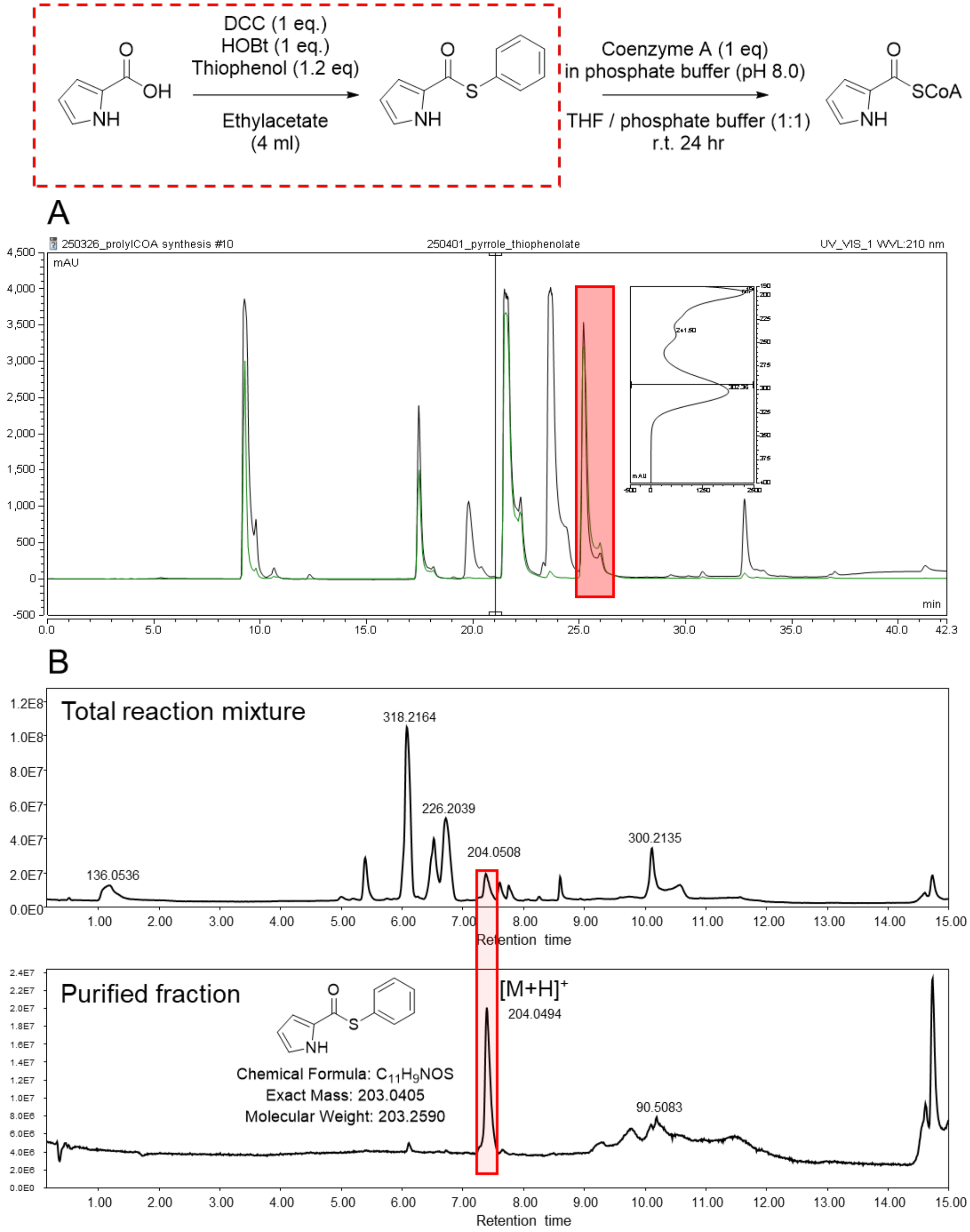

Figure S32. Purification and analysis of *S*-phenyl-pyrrole-2-carbothioate. A. HPLC-DAD chromatogram of reaction mixture monitored at 210 nm. **B.** TICs of crude reaction mixture and purified product analyzed by Method 2.

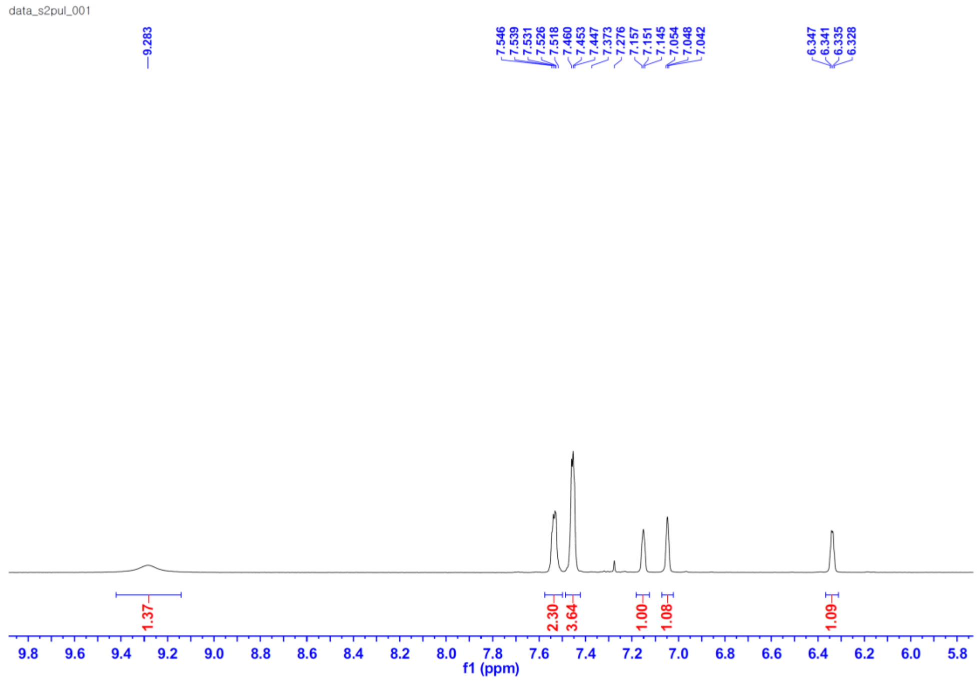

Figure S33. ^{1}H-NMR spectrum of *S*-phenyl-pyrrole-2-carbothioate ($CDCl_3$, 500 MHz).

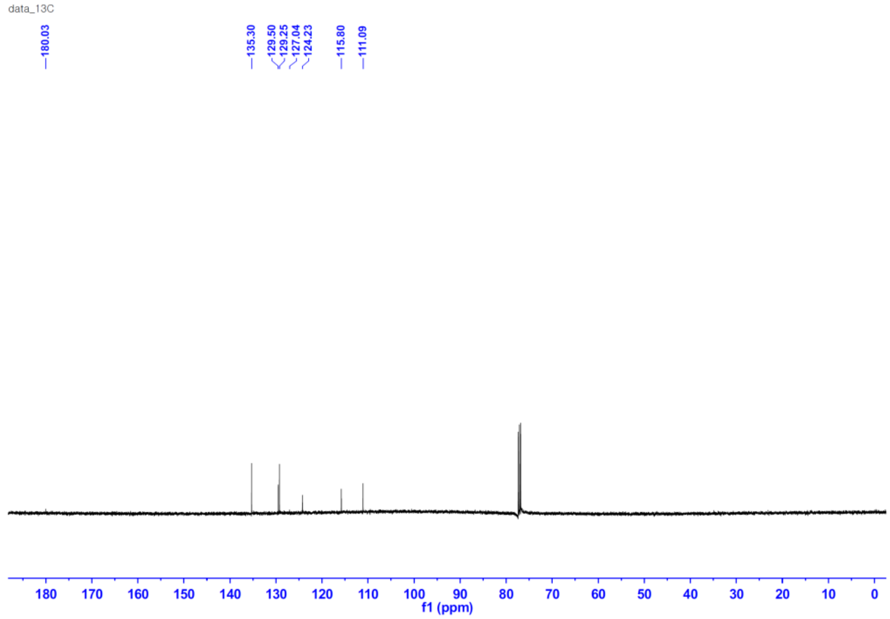

Figure S34. ^{13}C-NMR spectrum of *S*-phenyl-pyrrole-2-carbothioate ($CDCl_3$, 125 MHz).

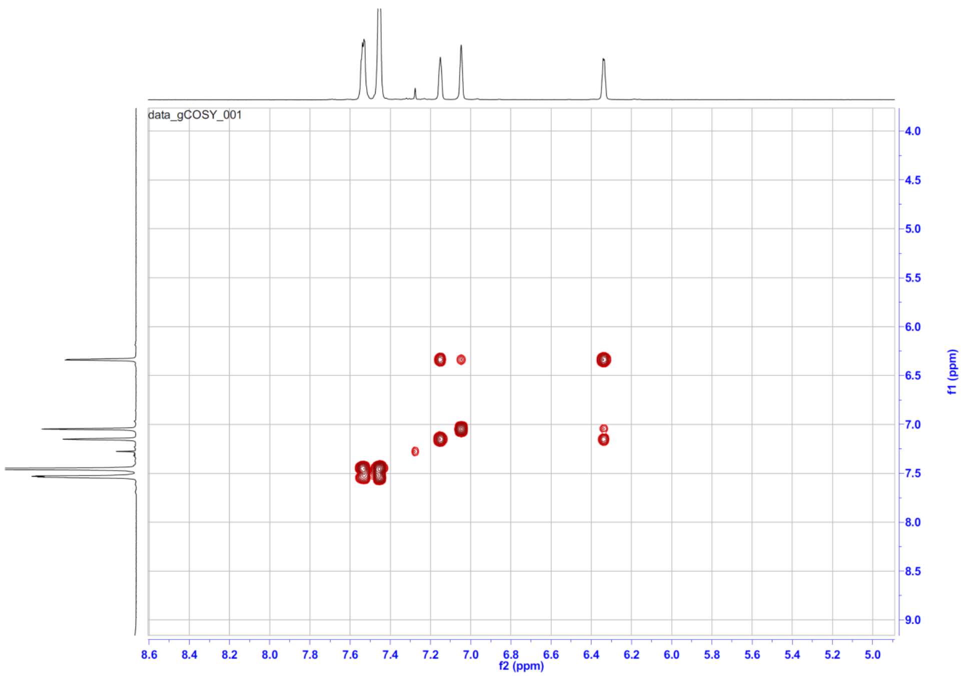

Figure S35. gCOSY spectrum of *S*-phenyl-pyrrole-2-carbothioate ($CDCl_3$).

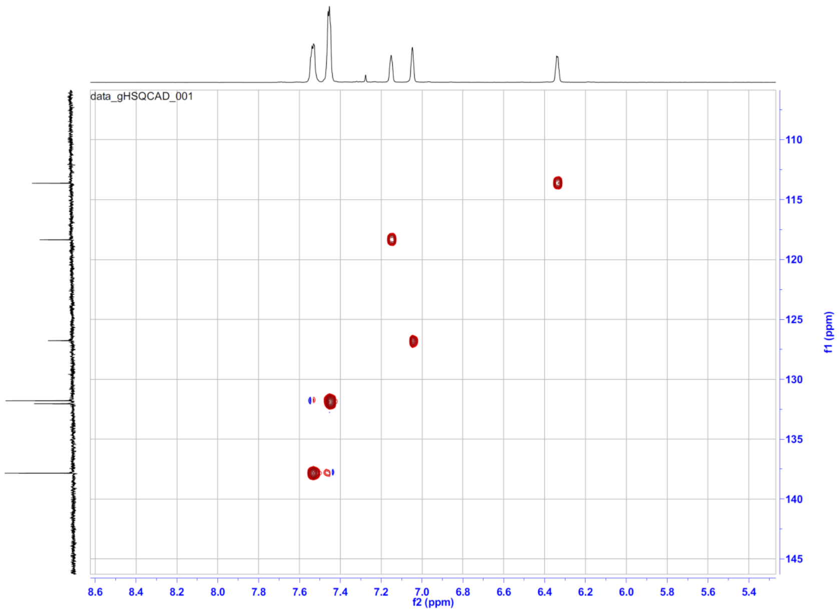

Figure S36. HSQC spectrum of *S*-phenyl-pyrrole-2-carbothioate ($CDCl_3$).

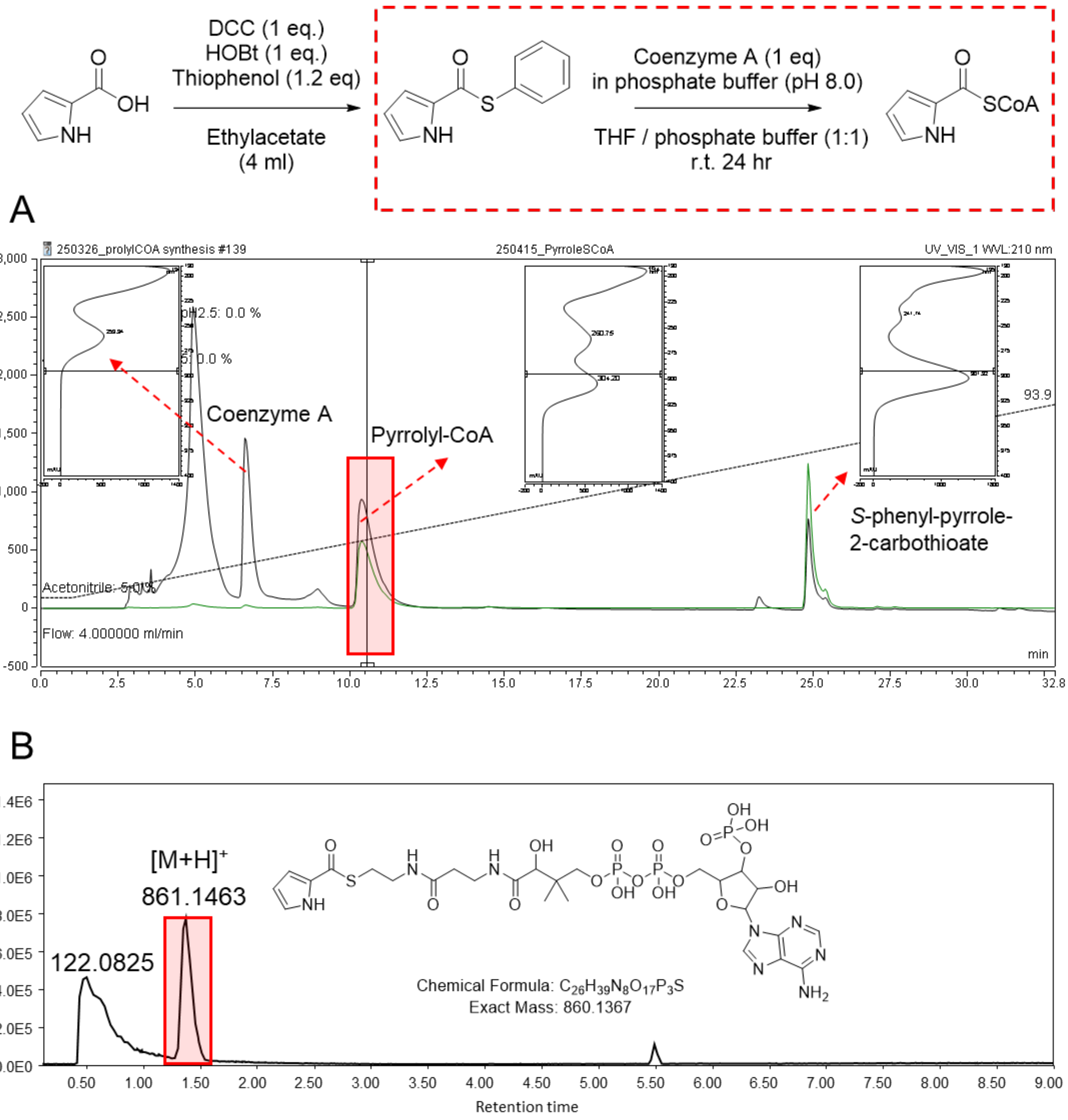

Figure S37. HPLC and MS analysis of CoA exchange reaction for pyrrolyl-CoA synthesis. A. The production of pyrrolyl-CoA was confirmed by its characteristic absorbance at 260.8 nm and 304.2 nm. **B.** TIC of purified pyrrolyl-CoA using Method 4. An overnight reaction with coenzyme A yielded 1.7 mg of pyrrolyl-CoA from 3.0 mg of *S*-phenyl-pyrrole-2-carbothioate.

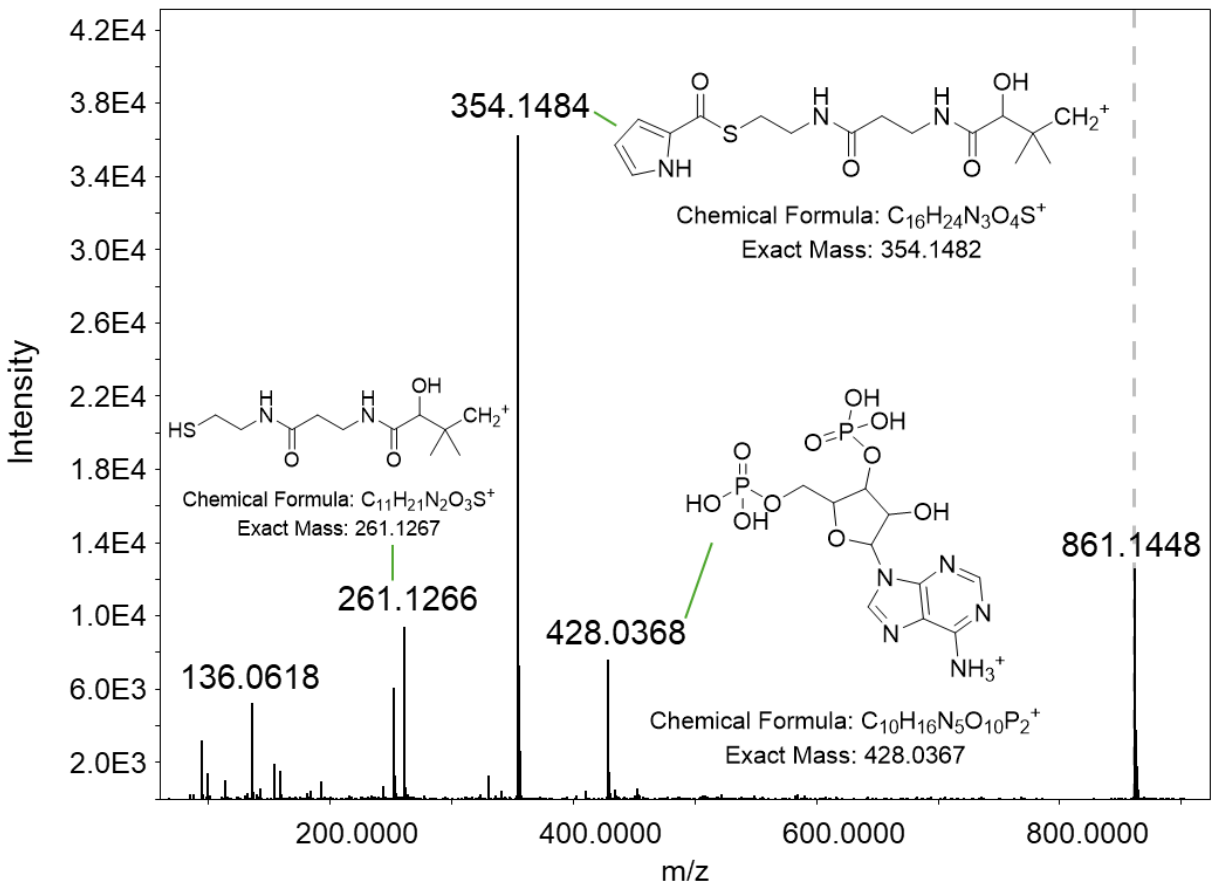

Figure S38. MS/MS analysis of pyrrolyl-CoA.

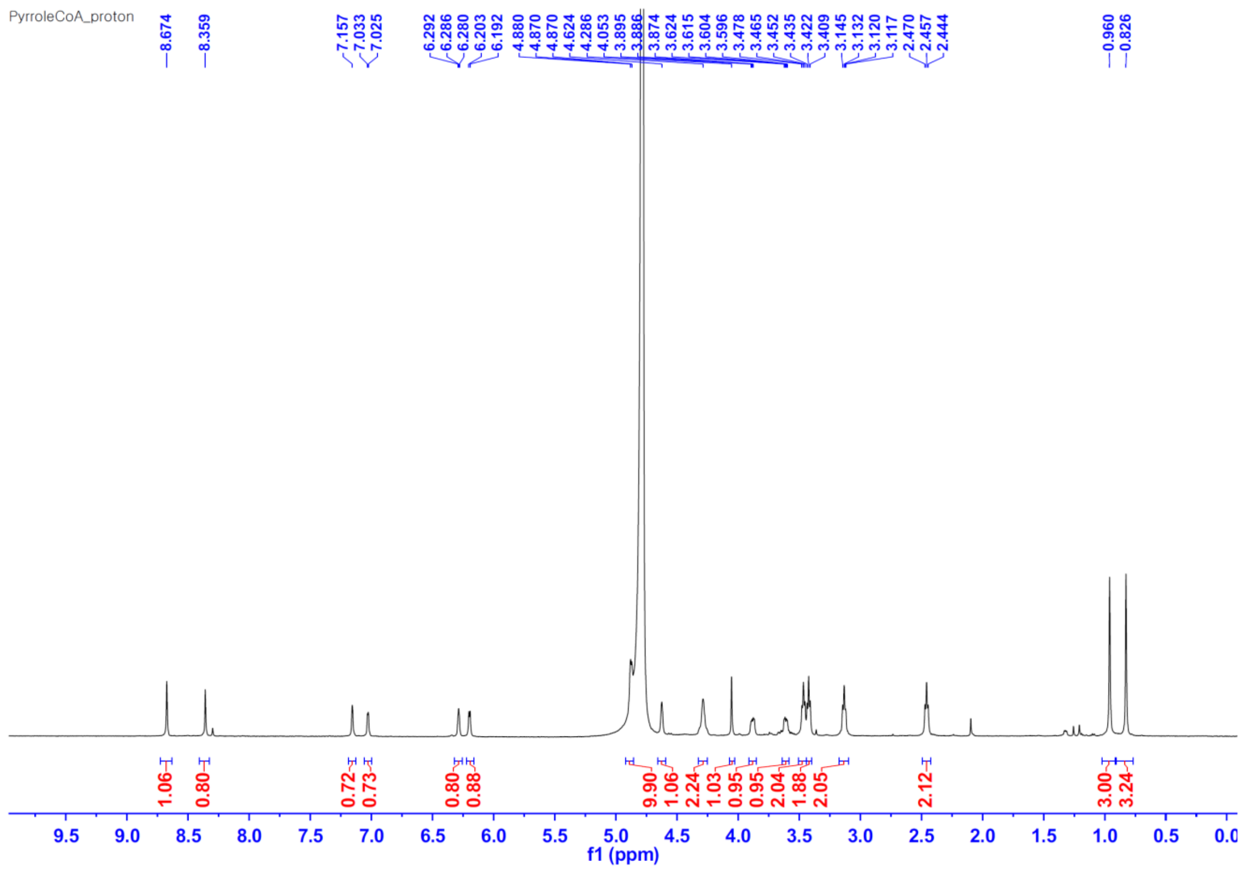

Figure S39. ^{1}H-NMR spectrum of pyrrolyl-CoA (D_2O, 500 MHz).

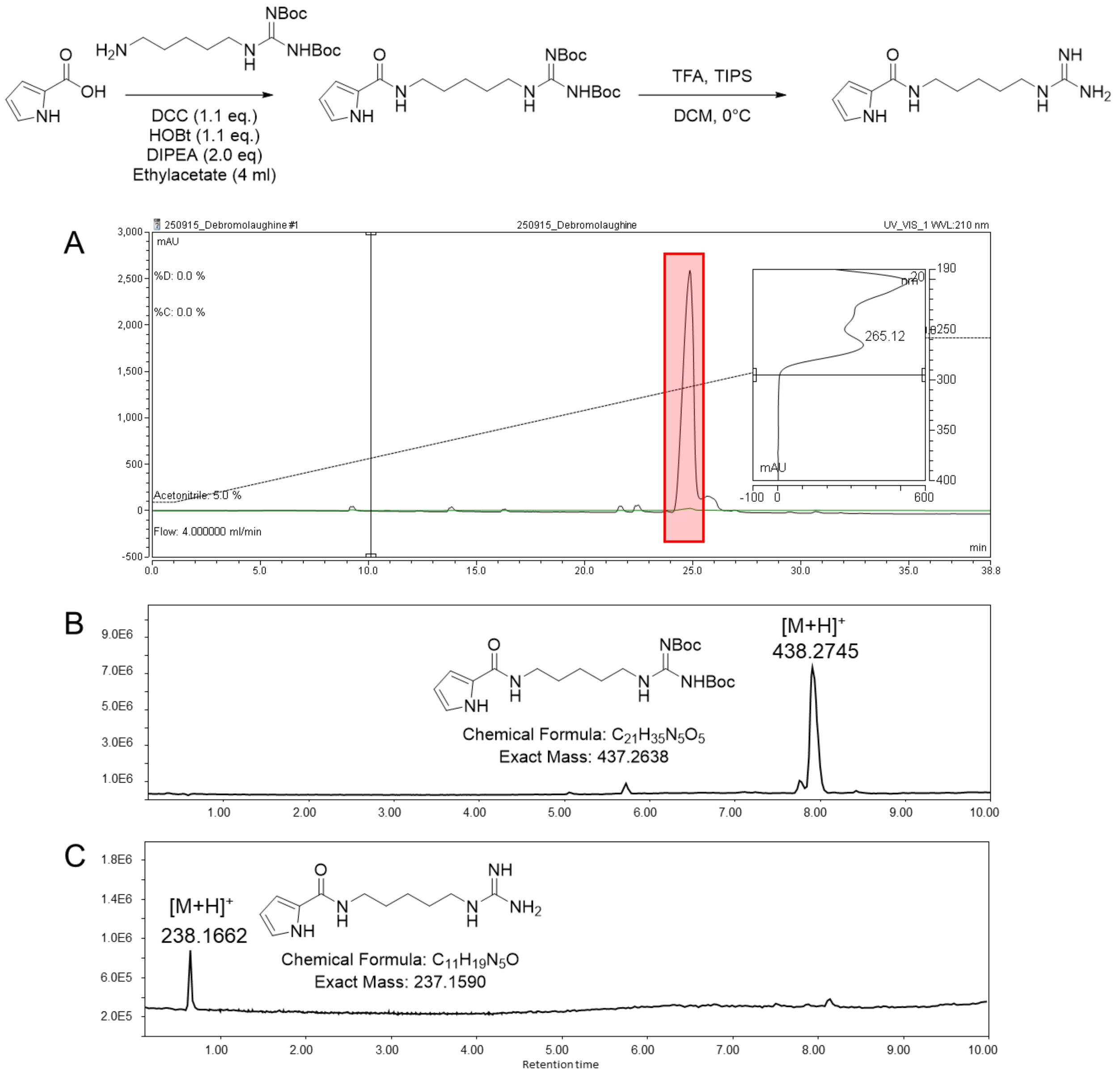

Figure S40. HPLC and MS analysis of debromolaughine synthesis. A. HPLC chromatogram of crude reaction mixture after DCC coupling. The peak in the red box is Boc-protected product. **B.** TIC of purified *N,N*-bis-Boc-debromolaughine. **C.** TIC of debromolaughine after deprotection. TICs were analyzed by Method 2.

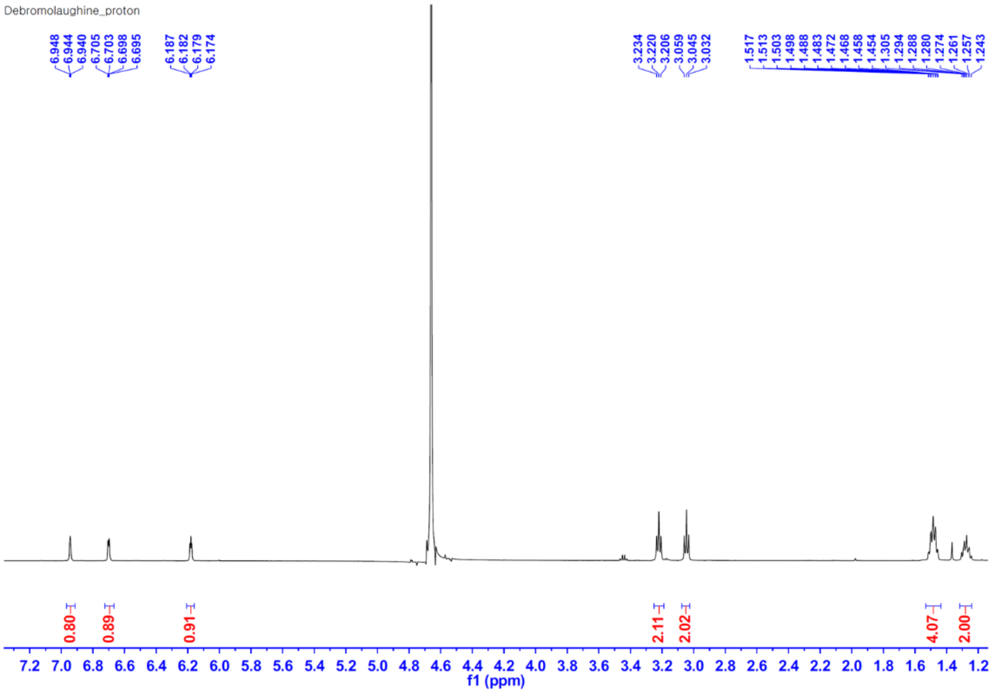

Figure S41. ^{1}H-NMR of debromolaughine (D_2O, 500 MHz).

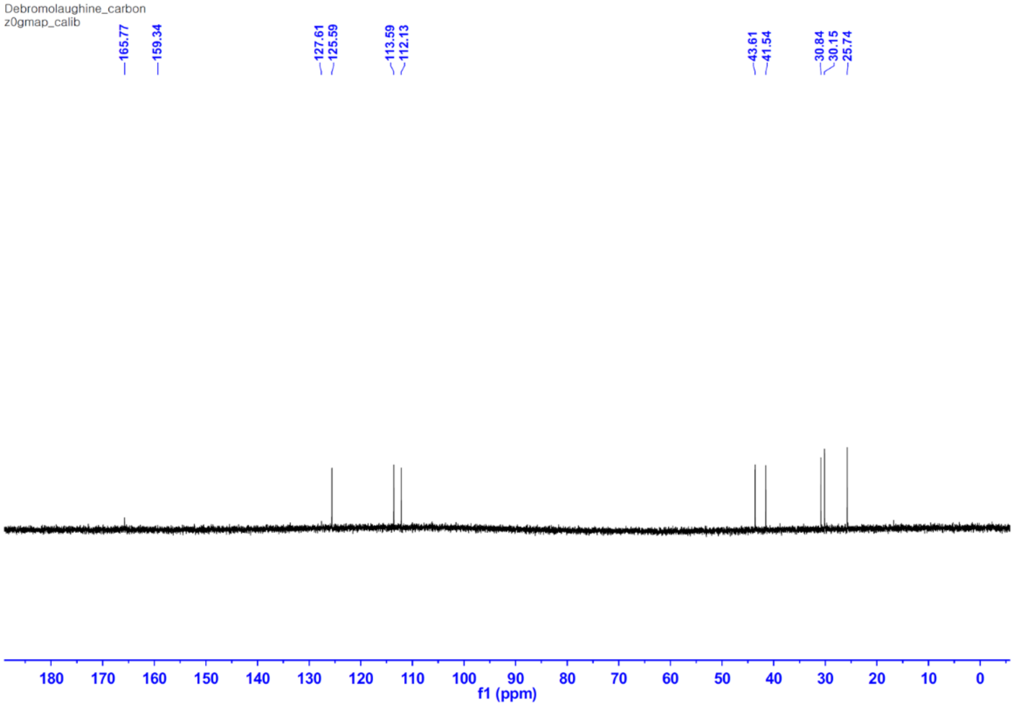

Figure S42. ^{13}C-NMR of debromolaughine (D_2O, 125 MHz).

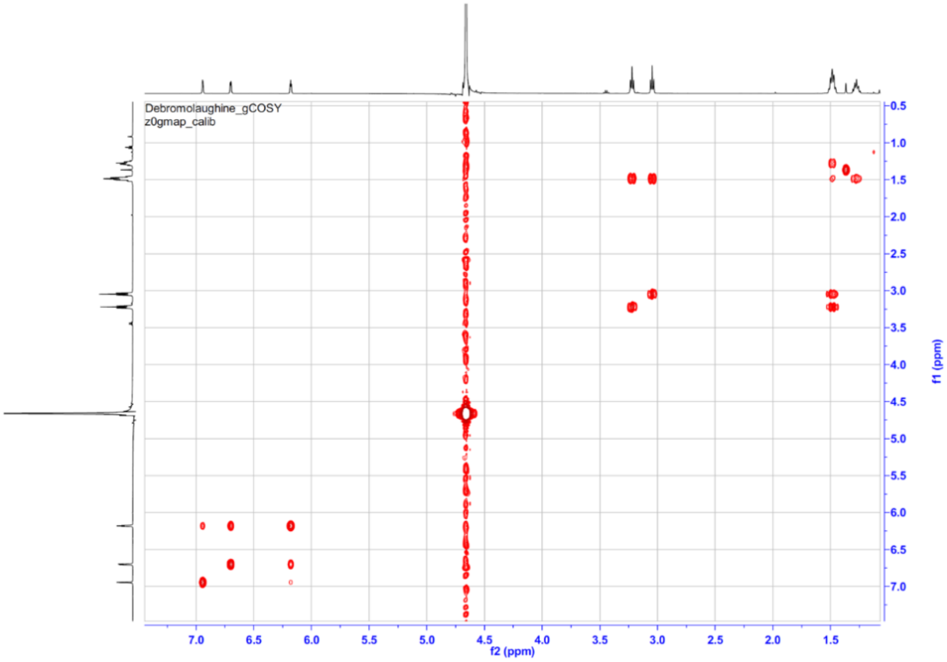

Figure S43. gCOSY of debromolaughine (D_2O).

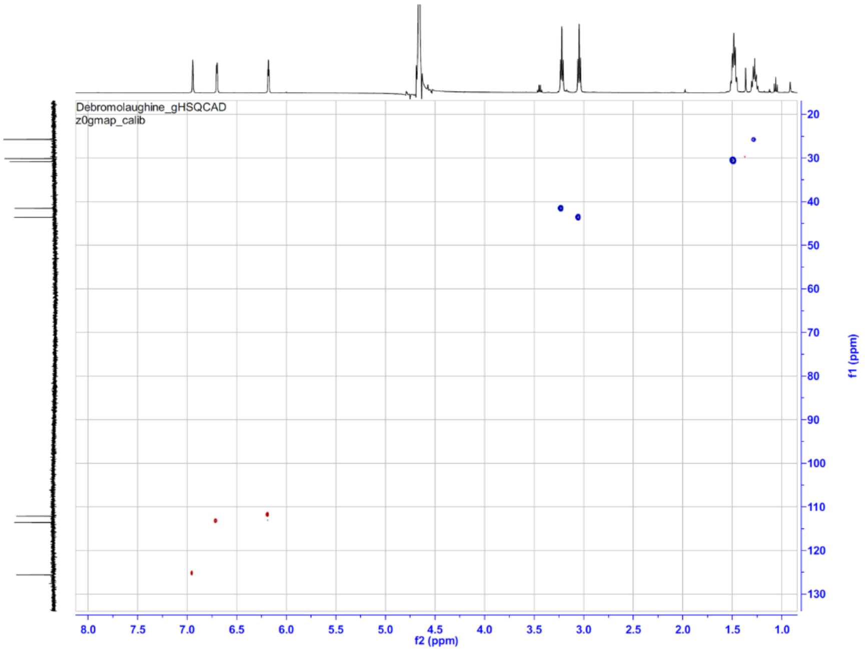

Figure S44. gHSQCAD of debromolaughine (D_2O).

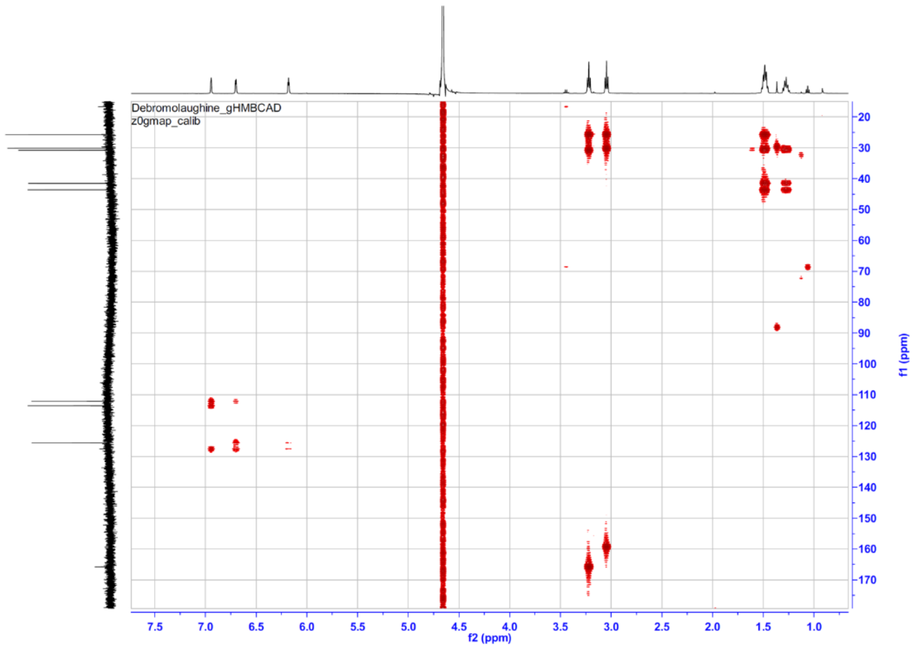

Figure S45. gHMBCAD of debromolaughine (D_2O).

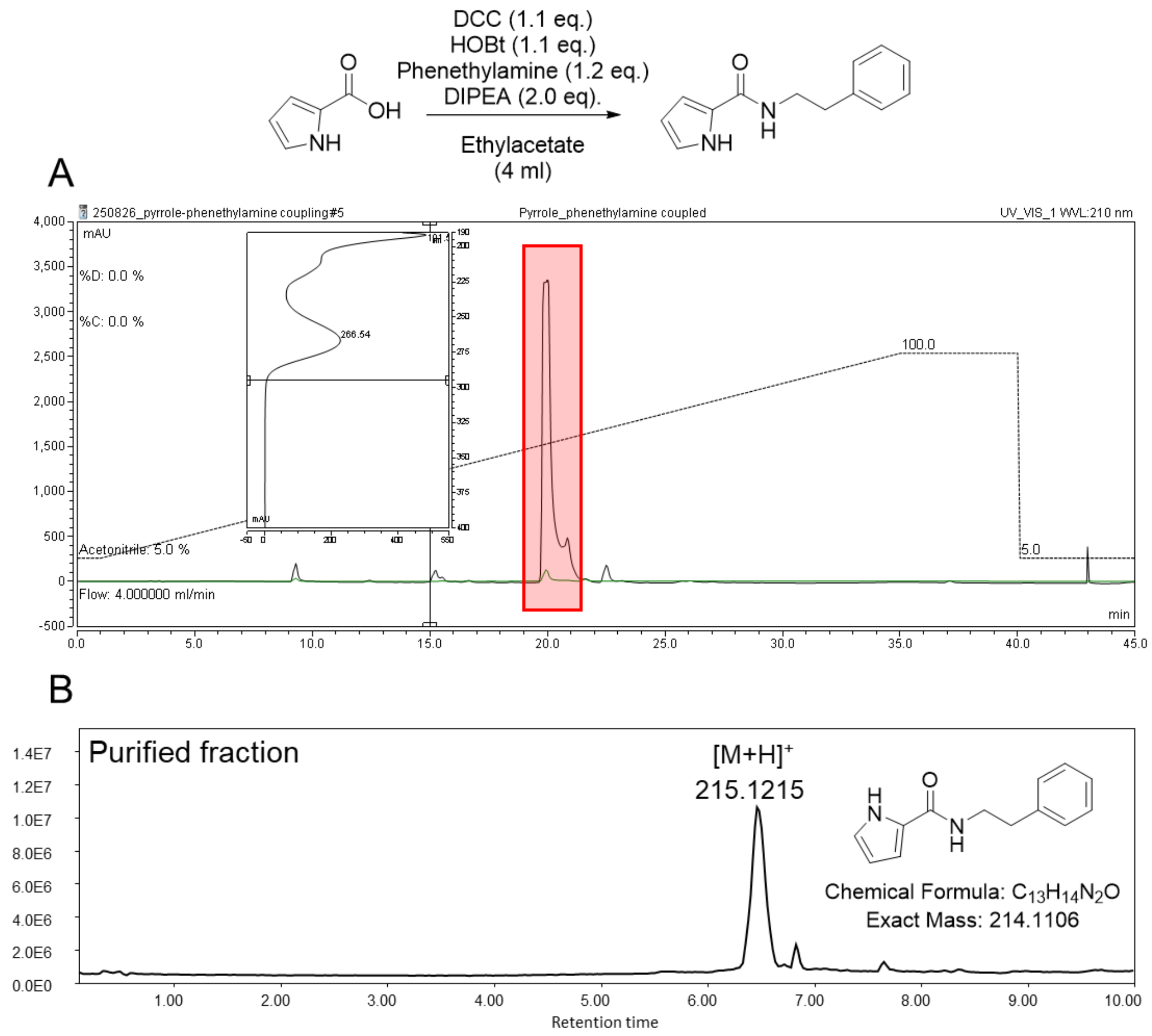

Figure S46. HPLC and MS analysis of *N*-(2-phenylethyl)-1*H*-pyrrole-2-carboxamide synthesis.

A. HPLC chromatogram of crude reaction mixture. **B.** TIC of purified *N*-(2-phenylethyl)-1*H*-pyrrole-2-carboxamide analyzed by Method 2.

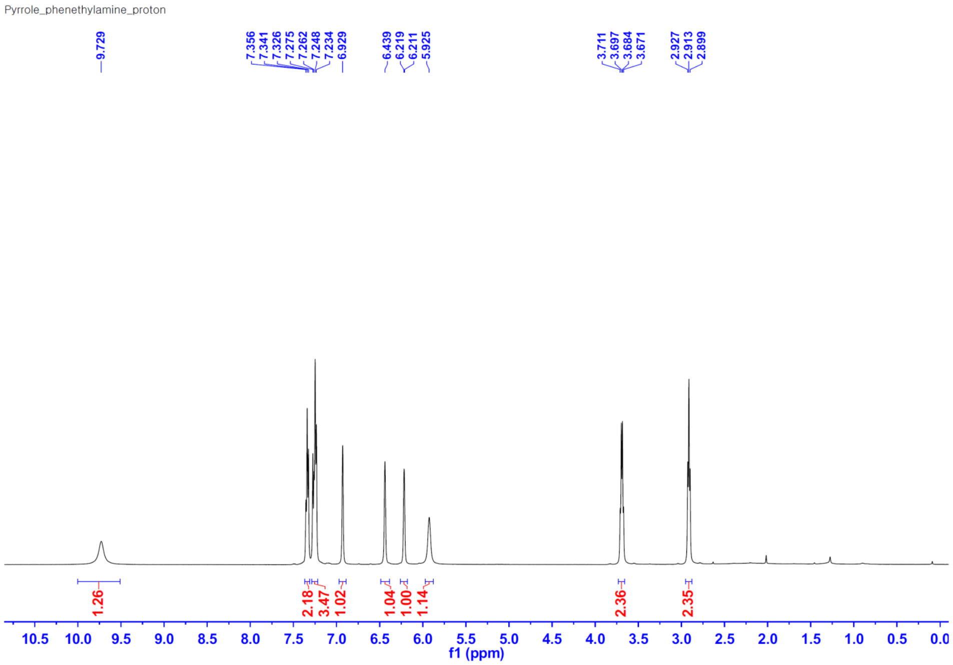

Figure S47. ^{1}H-NMR of *N*-(2-phenylethyl)-1*H*-pyrrole-2-carboxamide ($CDCl_3$, 500 MHz).

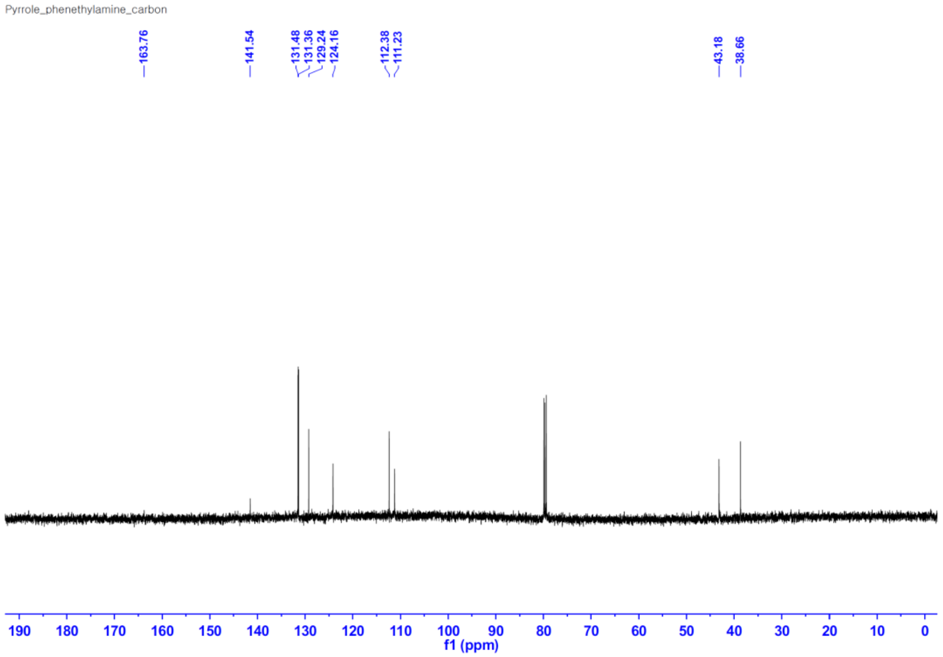

Figure S48. ^{13}C-NMR of *N*-(2-phenylethyl)-1*H*-pyrrole-2-carboxamide ($CDCl_3$, 125 MHz).

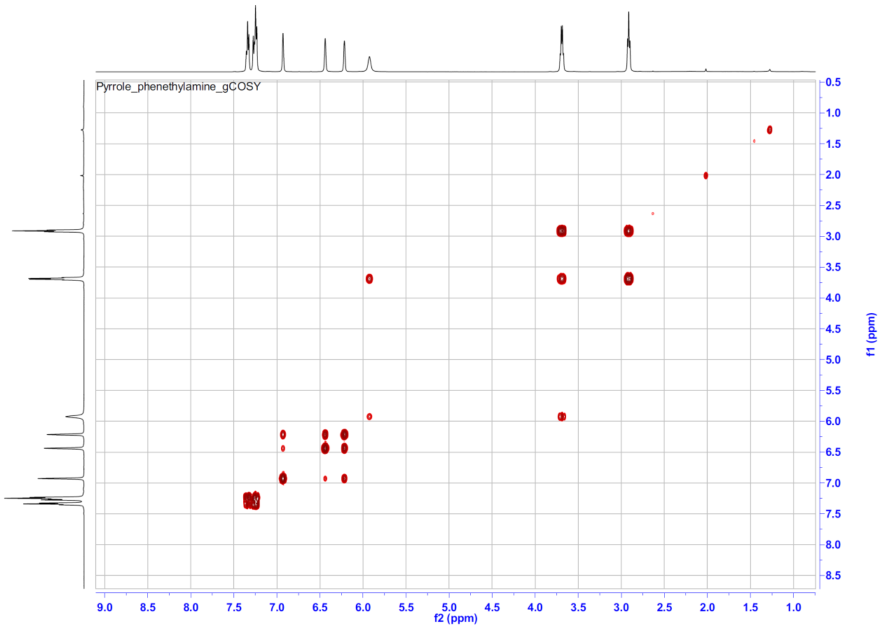

Figure S49. gCOSY of *N*-(2-phenylethyl)-1*H*-pyrrole-2-carboxamide ($CDCl_3$).

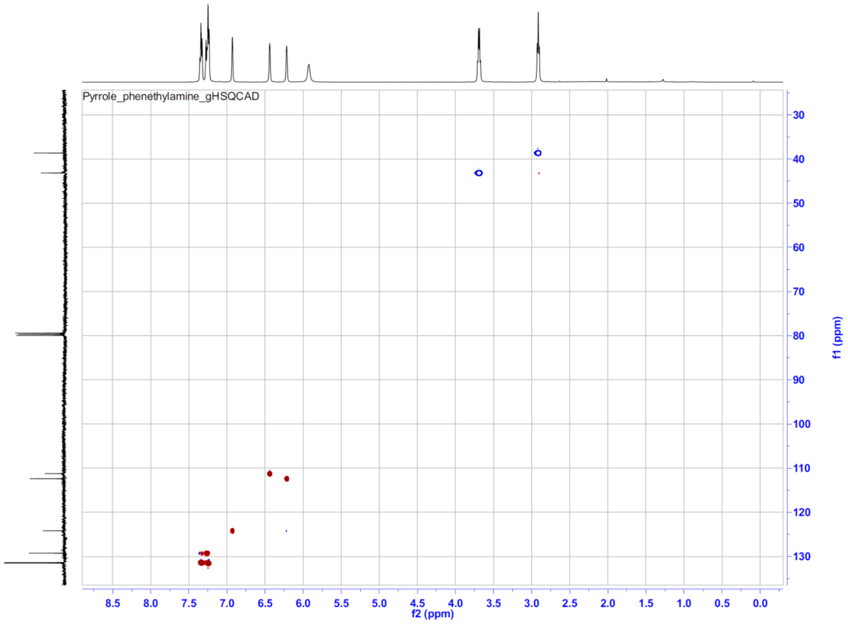

Figure S50. gHSQCAD of *N*-(2-phenylethyl)-1*H*-pyrrole-2-carboxamide ($CDCl_3$).

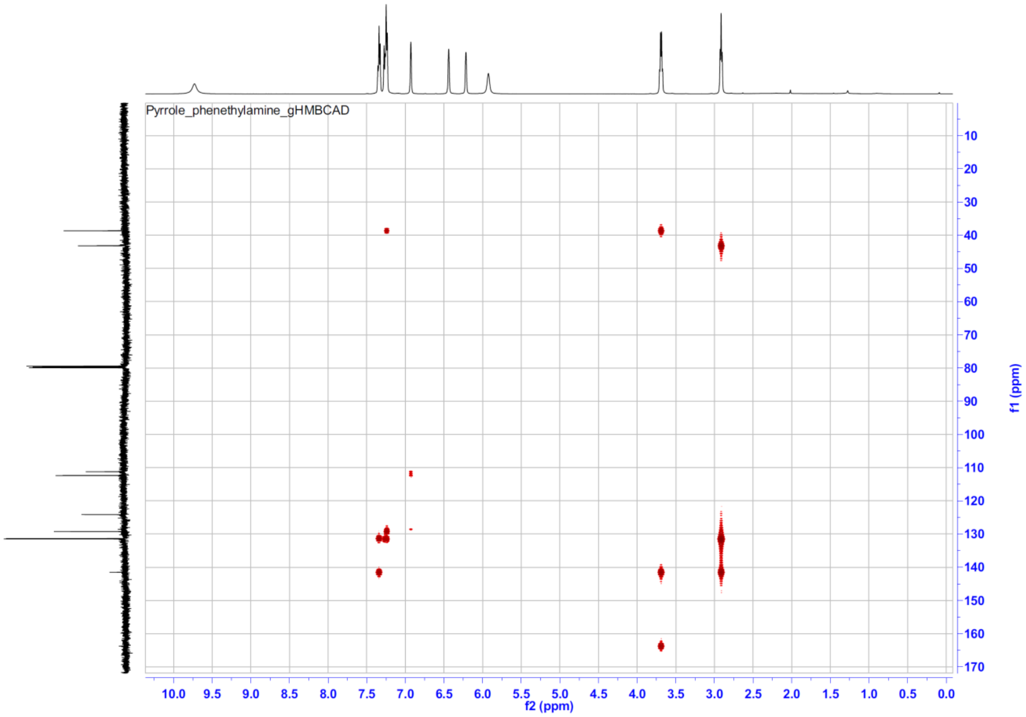

Figure S51. gHMBCAD of *N*-(2-phenylethyl)-1*H*-pyrrole-2-carboxamide ($CDCl_3$).

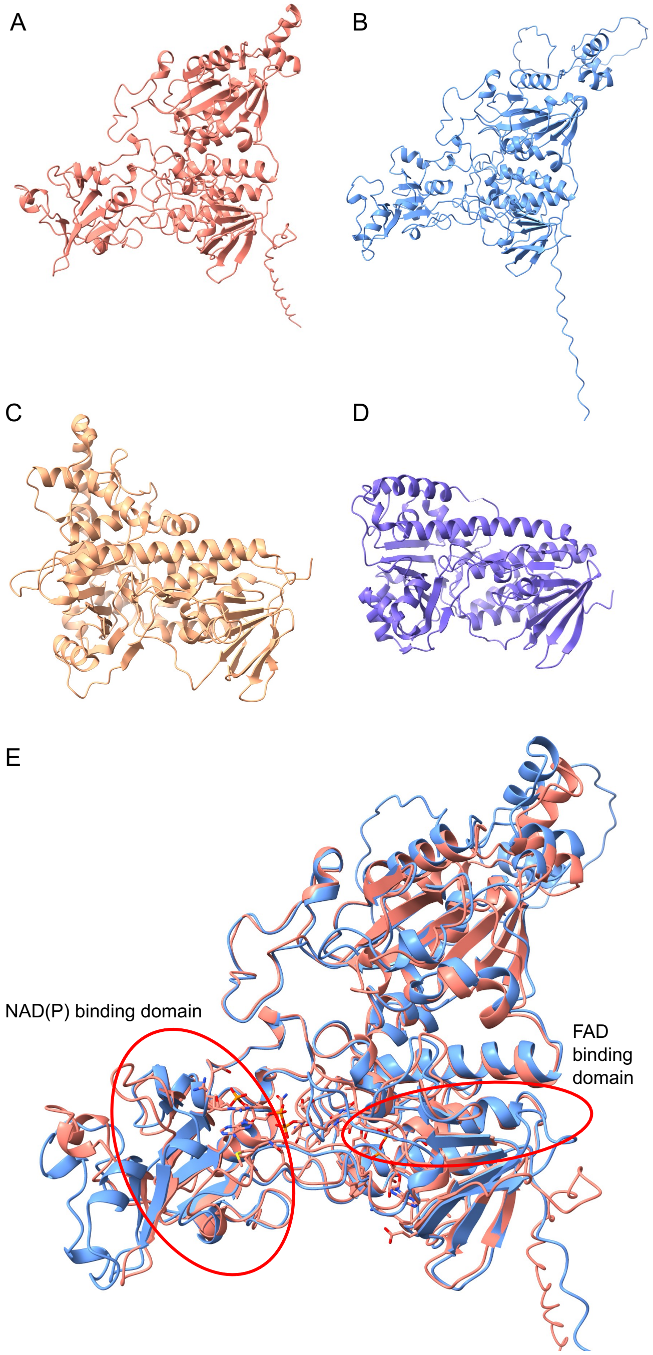

Figure S52. Comparative structural modeling of OroF and related enzymes. A. AlphaFold3-predicted structure of OroF. **B.** AlphaFold3-predicted structure of human ERFAD (GenBank number:NP_001095841.1). **C.** X-ray crystal structure of tryptophan halogenase PrnA (PDB

number: 2APG). **D.** X-ray crystal structure of pyrrole halogenase PltA (PDB number: 5DBJ).

E. Structural overlay of the AlphaFold3 model of OroF and human ERFAD.

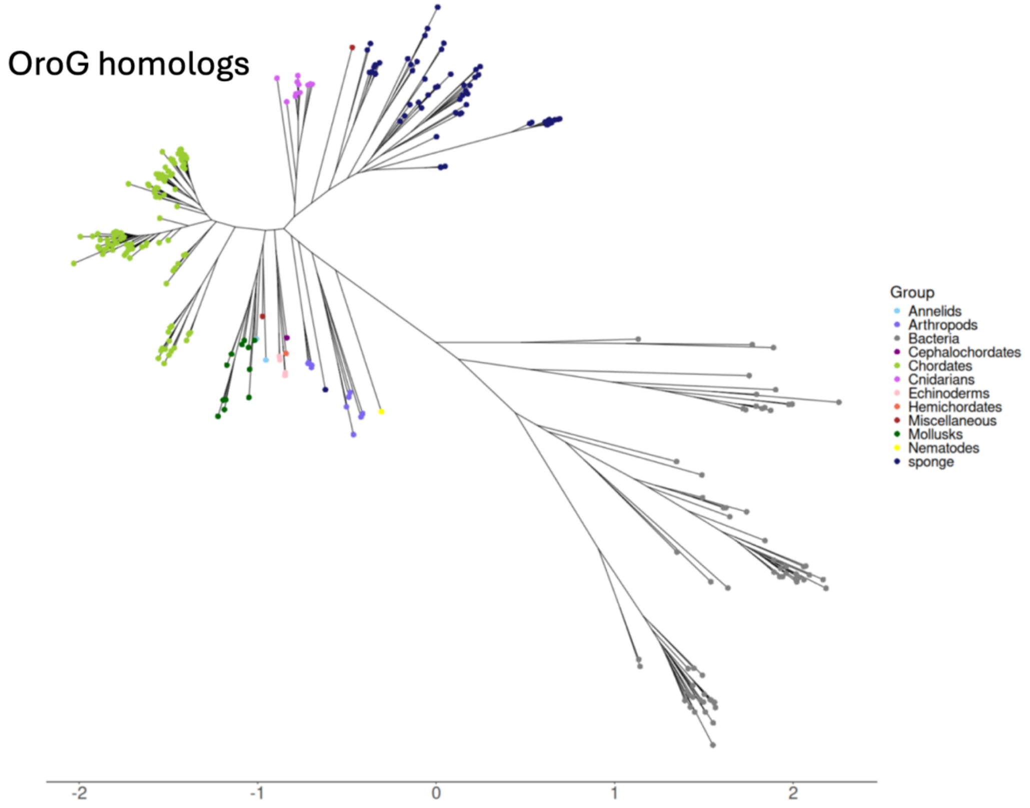
OroG homologs
Group
Annelids
Arthropods
Bacteria
Cephalochordates
Chordates
Cnidarians
Echinoderms
Hemichordates
Miscellaneous
Mollusks
Nematodes
sponge
-2
-1
0
1
2

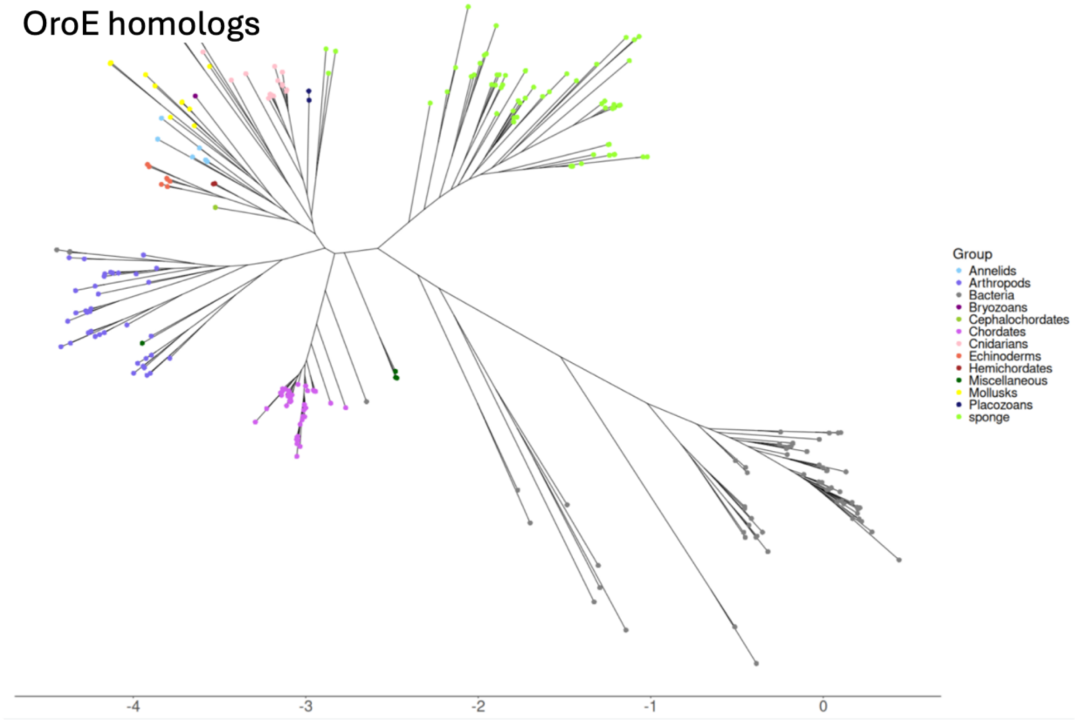
OroE homologs
Group
Annelids
Arthropods
Bacteria
Bryozoans
Cephalochordates
Chordates
Cnidarians
Echinoderms
Hemichordates
Miscellaneous
Mollusks
Placozoans
sponge
-4
-3
-2
-1
0

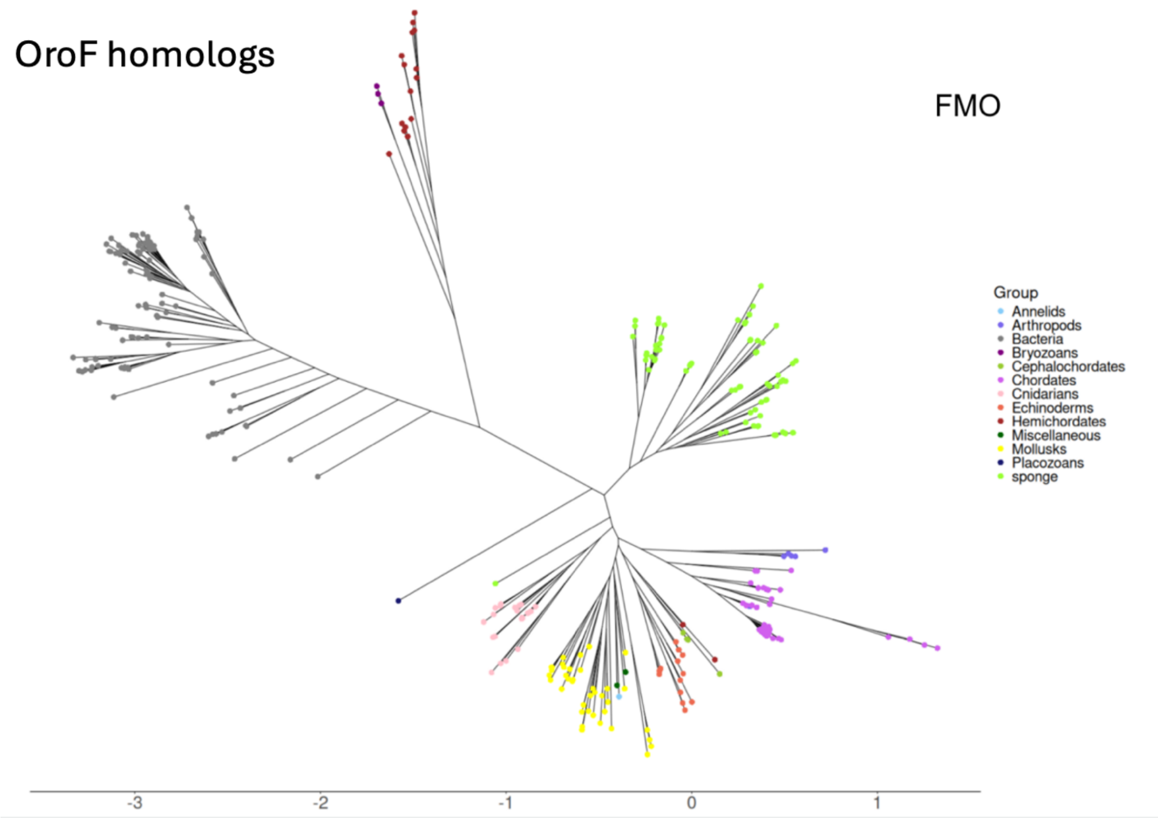
OroF homologs
FMO
Group
Annelids
Arthropods
Bacteria
Bryozoans
Cephalochordates
Chordates
Cnidarians
Echinoderms
Hemichordates
Miscellaneous
Mollusks
Placozoans
sponge
-3
-2
-1
0
1

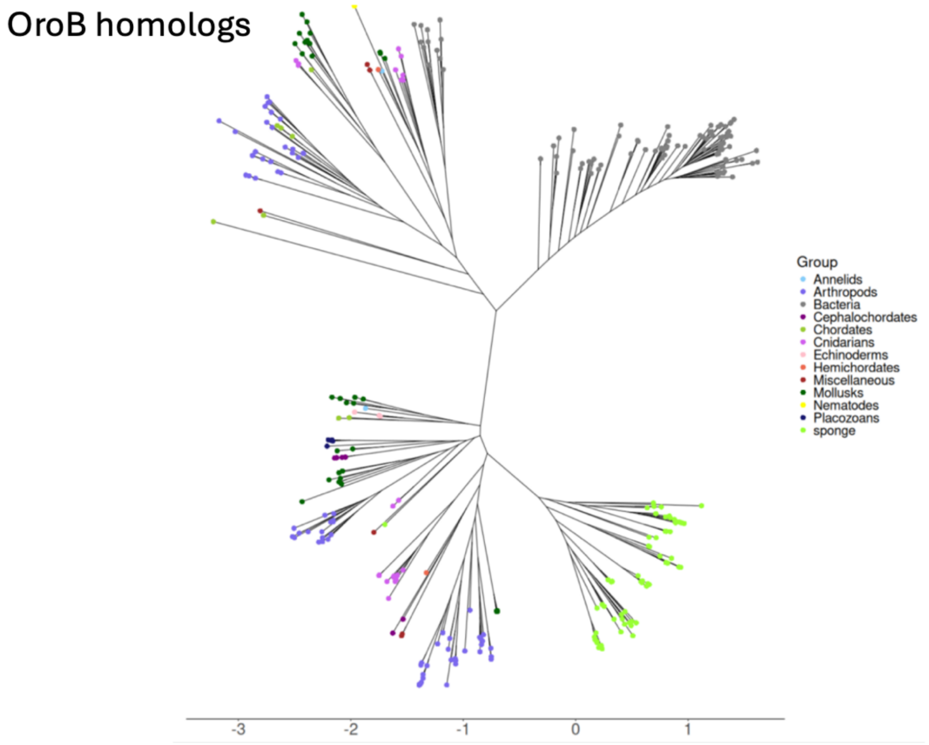
OroB homologs
Group
Annelids
Arthropods
Bacteria
Cephalochordates
Chordates
Cnidarians
Echinoderms
Hemichordates
Miscellaneous
Mollusks
Nematodes
Placozoans
sponge
-3
-2
-1
0
1

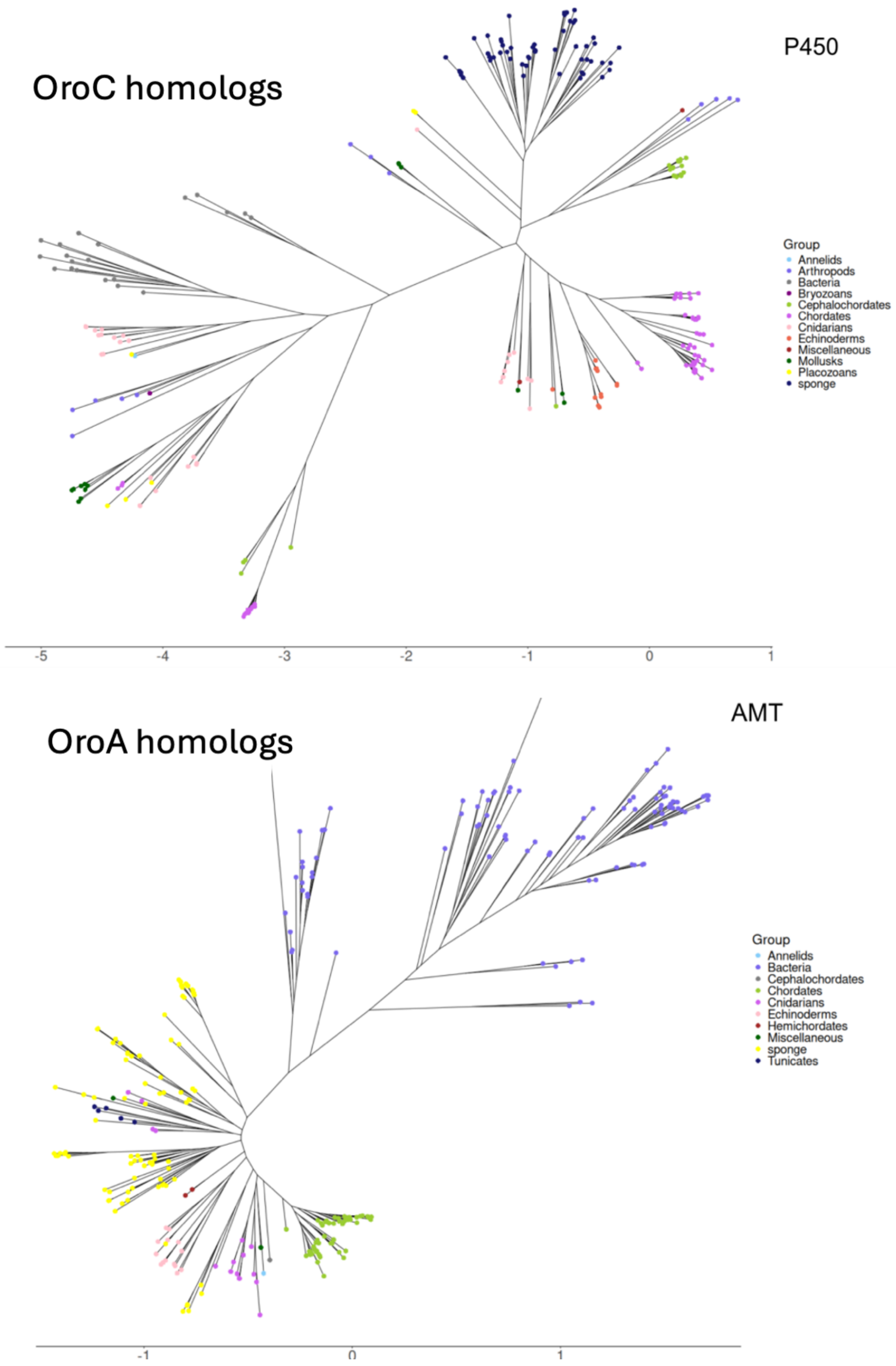

Figure S53. ML trees of Oro proteins and their homologs. These figures demonstrate that *oro* genes are core parts of animal genomes and are therefore derived from duplication and divergence of animal genes, rather than from horizontal gene transfer.

REFERENCES

(1) Zdouc, M. M.; Blin, K.; Louwen, N. L. L.; Navarro, J.; Loureiro, C.; Bader, C. D.; Bailey, C. B.; Barra, L.; Booth, T. J.; Bozhuyuk, K. A. J.; et al. MIBiG 4.0: advancing biosynthetic gene cluster curation through global collaboration. *Nucleic Acids Res* **2025**, *53* (D1), D678–D690.

(2) Stanke, M.; Keller, O.; Gunduz, I.; Hayes, A.; Waack, S.; Morgenstern, B. AUGUSTUS: ab initio prediction of alternative transcripts. *Nucleic Acids Res* **2006**, *34* (Web Server issue), W435–439.

(3) Jones, P.; Binns, D.; Chang, H. Y.; Fraser, M.; Li, W.; McAnulla, C.; McWilliam, H.; Maslen, J.; Mitchell, A.; Nuka, G.; et al. InterProScan 5: genome-scale protein function classification. *Bioinformatics* **2014**, *30* (9), 1236–1240.

(4) Sayers, E. W.; Beck, J.; Bolton, E. E.; Brister, J. R.; Chan, J.; Connor, R.; Feldgarden, M.; Fine, A. M.; Funk, K.; Hoffman, J.; et al. Database resources of the National Center for Biotechnology Information in 2025. *Nucleic Acids Res* **2025**, *53* (D1), D20–D29.

(5) Wang, Y.; Tang, H.; Wang, X.; Sun, Y.; Joseph, P. V.; Paterson, A. H. Detection of colinear blocks and synteny and evolutionary analyses based on utilization of MCScanX. *Nat Protoc* **2024**, *19* (7), 2206–2229.

(6) Navarro-Munoz, J. C.; Selem-Mojica, N.; Mullowney, M. W.; Kautsar, S. A.; Tryon, J. H.; Parkinson, E. I.; De Los Santos, E. L. C.; Yeong, M.; Cruz-Morales, P.; Abubucker, S.; et al. A

computational framework to explore large-scale biosynthetic diversity. *Nat Chem Biol* **2020**, *16* (1), 60–68.

(7) Venugopalan, A.; Schmidt, E. W. Animal-Encoded Nonribosomal Pathway to Bursatellin Analogs. *J Am Chem Soc* **2025**, *147* (8), 6623–6632.

(8) Lin, Z.; Agarwal, V.; Cong, Y.; Pomponi, S. A.; Schmidt, E. W. Short macrocyclic peptides in sponge genomes. *Proc Natl Acad Sci U S A* **2024**, *121* (11), e2314383121.

(9) Teufel, F.; Almagro Armenteros, J. J.; Johansen, A. R.; Gislason, M. H.; Pihl, S. I.; Tsirigos, K. D.; Winther, O.; Brunak, S.; von Heijne, G.; Nielsen, H. SignalP 6.0 predicts all five types of signal peptides using protein language models. *Nat Biotechnol* **2022**, *40* (7), 1023–1025.

(10) Hallgren, J.; Tsirigos, K. D.; Pedersen, M. D.; Almagro Armenteros, J. J.; Marcatili, P.; Nielsen, H.; Krogh, A.; Winther, O. DeepTMHMM predicts alpha and beta transmembrane proteins using deep neural networks. *bioRxiv* **2022**, 2022.2004.2008.487609.

(11) Abramson, J.; Adler, J.; Dunger, J.; Evans, R.; Green, T.; Pritzel, A.; Ronneberger, O.; Willmore, L.; Ballard, A. J.; Bambrick, J.; et al. Addendum: Accurate structure prediction of biomolecular interactions with AlphaFold 3. *Nature* **2024**, *636* (8042), E4.

(12) Miller, B. W.; Lim, A. L.; Lin, Z.; Bailey, J.; Aoyagi, K. L.; Fisher, M. A.; Barrows, L. R.; Manoil, C.; Schmidt, E. W.; Haygood, M. G. Shipworm symbiosis ecology-guided discovery of an antibiotic that kills colistin-resistant Acinetobacter. *Cell Chem Biol* **2021**, *28* (11), 1628–1637 e1624.

(13) El Gamal, A.; Agarwal, V.; Diethelm, S.; Rahman, I.; Schorn, M. A.; Sneed, J. M.; Louie, G. V.; Whalen, K. E.; Mincer, T. J.; Noel, J. P.; et al. Biosynthesis of coral settlement cue tetrabromopyrrole in marine bacteria by a uniquely adapted brominase-thioesterase enzyme pair. *Proc Natl Acad Sci U S A* **2016**, *113* (14), 3797–3802.

(14) Mohanty, I.; Moore, S. G.; Yi, D.; Biggs, J. S.; Gaul, D. A.; Garg, N.; Agarwal, V. Precursor-Guided Mining of Marine Sponge Metabolomes Lends Insight into Biosynthesis of Pyrrole-Imidazole Alkaloids. *ACS Chem Biol* **2020**, *15* (8), 2185–2194.

(15) Agarwal, V.; El Gamal, A. A.; Yamanaka, K.; Poth, D.; Kersten, R. D.; Schorn, M.; Allen, E. E.; Moore, B. S. Biosynthesis of polybrominated aromatic organic compounds by marine bacteria. *Nat Chem Biol* **2014**, *10* (8), 640–647.

(16) Darnowski, M. G.; Lanosky, T. D.; Paquette, A. R.; Boddy, C. N. Synthesis of a Constitutional Isomer of Armeniaspirol A, Pseudoarmeniaspirol A, via Lewis Acid-Mediated Rearrangement. *J Org Chem* **2022**, *87* (22), 15634–15643.
